# Integrating AI/ML and multi-omics approaches to investigate the role of TNFRSF10A/TRAILR1 and its potential targets in pancreatic cancer

**DOI:** 10.1016/j.compbiomed.2025.110432

**Published:** 2025-05-26

**Authors:** Sudhanshu Sharma, Rajesh Singh, Shiva Kant, Manoj K. Mishra

**Affiliations:** a Cancer Research Center, Department of Biological Sciences, Alabama State University, Montgomery, AL, 36104, USA; b Microbiology, Biochemistry, and Immunology, Cancer Health Equity Institute, Morehouse School of Medicine, Atlanta, GA, USA; c Department of Biology and Environmental Sciences, College of Sciences, Auburn University of Montgomery, Montgomery, USA

**Keywords:** Artificial intelligence, Multi-omics, Cancer, Machine learning, MD simulations, TNFRSF10A

## Abstract

Pancreatic ductal adenocarcinoma (PDAC) remains one of the most lethal malignancies, with a five-year survival of under 10 % despite current therapies. Aggressive tumor biology, a desmoplastic stroma that limits drug delivery and immune cell infiltration, and profound resistance to apoptosis make it more complex to treat. Here, we describe a multi-layered system biology and drug discovery pipeline that integrates bulk genomics, single-cell spatial transcriptomics, proteomics, competing endogenous RNA (ceRNA) network analysis, and deep learning-driven quantitative structure-activity relationship (QSAR) modeling. By implementing this pipeline, we predicted that TNFRSF10A encodes for the TRAILR1 death receptor as a potential therapeutic target in PDAC. Mutational and expressional analysis also confirmed TNFRSF10A as a putative target in PDAC. Cancer cells within the PDAC microenvironment exhibit aberrantly elevated TNFRSF10A expression. Immune-excluded tumor niches and pro-survival signaling link this elevated expression. Using an advanced transformer-based deep learning approach, SELFormer, combined with QSAR analysis-based virtual screening, we identified previously unexplored FDA-approved drugs and natural compounds, i.e., Temsirolimus, Ergotamine, and capivasertib, with potential TRAILR1 modulatory effects. During molecular dynamics simulations, these repurposed candidates showed the highest binding affinities against TNFRSF10A for 300 ns. These showed favorable binding energies (MM-PBSA), minimal RMSD drift, PCA, and SASA. We propose TNFRSF10A as a therapeutically important PDAC vulnerability nurtured by spatially resolved expression patterns and dynamic molecular modeling. This study has used a novel integration of AI-implemented chemical modeling, high-throughput screening, and a multi-omics approach to unravel and pharmacologically target a cancer compartment-specific weakness in a notoriously drug-resistant cancer.

## 1. Introduction

Pancreatic ductal adenocarcinoma is an aggressive malignant carcinoma estimated to be the leading cause of cancer death in the upcoming years. Undeterred by advancements in therapeutic options such as surgical removal and chemotherapeutics like gemcitabine, the five-year survival remains 10–12 % [1]. A marked feature of PDAC is its immunosuppressive and fibrotic tumor microenvironment, characterized by dense stromal desmoplasia composed of cancer-associated fibroblasts (CAFs), suppressive immune cells [2], and extracellular matrix [3]. The desmoplastic stroma makes up to ~70 % of the tumor mass, forming a physical and metabolic barrier that limits vascular perfusion, impedes immune cell infiltrations, and reduces or eliminates the efficacy of chemotherapy and immunotherapy [3]. PDAC has shown minimal responses to immune checkpoint inhibitors in clinical trials compared to melanoma or lung cancer, largely attributed to T-cell exclusion and stromal sequestration of tumor antigens [4]. Concurrently, PDAC cells themselves harbor intrinsic resistance mechanisms to apoptosis and therapy-induced cell death, including constitutive activation of pro-survival pathways (KRAS-NF kappa B, PI3K-AKT-mTOR) and the upregulation of anti-apoptotic molecules like the bcl-2, XIAPs, and c-FLIPS [5]. These features underline the need for therapeutic strategies to combat micro-environmental and cell-intrinsic resistance. A promising approach is to target context-specific vulnerabilities at the juncture of stroma and tumor. Recent studies have shown how to decipher the compartmental heterogeneity of the PDAC. One study focused on spatial transcriptomic profiling that revealed distinct transcriptional programs in cancer cells and CAFs at the tumor-invasive front versus the tumor core [6]. Another study showed heightened TNF-α/NF-kappaB signaling, hypoxia, and epithelial-mesenchymal transition (EMT) pathways [7]. Such observations suggest that certain signaling nodes may be important in specific tumor niches differently (e.g., hypoxic invasive fronts enriched in inflammatory cytokines). In this study, we analyzed integrative multi-omics data, including spatial transcriptomics, bulk genomics, and proteomics, to predict molecular targets that exhibit differential expression and are likely to be spatially enriched in key tumor compartments, such as fibroblast-rich zones and immune-excluded zones. The system biology approaches inculcation helped us elucidate the regulatory network controlling these targets, such as the microRNAs that tune the target expression. However, translating such multi-omics insight into concrete therapy requires an equally sophisticated drug discovery approach, given the paucity of “druggable” mutations in PDAC, but historically undruggable until recently. Therefore, we present a multi-layered system biology and drug discovery approach to uncover actionable PDAC vulnerabilities, including bulk transcriptomics (six independent PDAC patient cohorts), to identify robust differentially expressed genes in tumor vs normal tissue samples. Apart from single-cell and spatial micro proteomics to monitor feasible disease-related protein biomarkers [8,9], single-cell and spatial transcriptomics enable to map the localization of candidate genes in the tumor microenvironment and identify which cell types express them. We performed a ceRNA network analysis to map the microRNAs, providing insights into the upstream regulatory mechanisms that can be leveraged to modulate target expression. Our study identified TNFRSF10A as the most suitable and upregulated candidate, specifically enriched in malignant epithelial cells at the leading edge of tumors near stromal regions. Therefore, we hypothesize that targeting the TNFRSF10A in PDAC could bypass some resistance mechanisms and directly induce cancer death even without a robust T-cell response.

We leveraged deep learning and molecular modeling to test this hypothesis to predict the repurposed drug candidates that could engage TRAIL-R1. This AI-driven approach yielded three compelling candidates: temsirolimus, ergotamine, and capivasertib. Temsirolimus, an mTOR inhibitor and rapamycin analog, has already received approval for treating renal cell carcinoma. Notably, researchers have demonstrated that blocking the mTOR pathway can enhance death receptor-mediated apoptosis by downregulating c-FLIP and surviving, which are key inhibitors of the extrinsic apoptosis pathway [10]. After integrating the AI-machine learning approach, we performed extensive MD simulations for a 300ns timescale with each compound, confirming stable binding and favorable energetics, particularly for temsirolimus and ergotamine. These results allowed us to understand the mechanistic importance of therapeutic targeting of TNFRSF10A concerning the complex tumor microenvironment. This study merges high-throughput omics data with advanced AI-assisted drug discovery to predict a novel therapeutic target. i.e., TNFRSF10A and reasonably small molecule antagonists to PDAC. Therefore, in this study, we discuss the implications of these findings in the context of PDAC biology, tumor microenvironment, and the emerging paradigm of AI-augmented precision oncology.

## 2. Materials and methodology

### 2.1. Data retrieval

Data with extensive PDAC information was sourced from six cohorts using Gene Expression Omnibus (GEO): GSE30134, GSE32676, GSE71989, GSE15471, GSE22780, and GSE16515. All six datasets were downloaded with the GSEAPY library using Python 3.0. Machine learning was incorporated with the Bayesian robust inference and random forest algorithms to perform the analysis. We selected the differentially expressed genes (DEGs) based on their posterior probability (PP) and fold change (FC). We also installed Python libraries for data manipulation, statistical analysis, and visualization. Further, we procured spatial transcriptomics data by using the comprehensive multi-omics information from the publicly available single-cell RNA sequencing dataset GSE202051, which contains primary PDAC tumor specimens profiled using the 10X Genomics Visium platform (10X Genomics, Pleasanton, CA). This dataset provides complete transcriptomic profiles with spatial coordinates aligned to histological samples from healthy and affected individuals from The Cancer Genome Atlas (TCGA). Integrating both data types would help detect gene dosage effects due to copy number variations (CNVs) and somatic SNVs (single-nucleotide variations) expressed at transcriptome levels. We obtained.h5ad data files specifically designed for single-cell and spatial transcriptomics, which were loaded using the SCANPY package in Python 3.0. We also queried the CPTAC PDAC proteomic data to understand the relevance of the top predictions in tumor versus normal pancreatic tissues by visualization of the gene-protein correlations.

### 2.2. Data preprocessing, visualization, and differential expression analysis

Once we obtained the datasets, we preprocessed the data by filtering out low-expressed genes, addressing the missing values, normalizing the data, and generating a matrix in a suitable format for analysis. We merged the metadata from all six datasets (GSE30134, GSE32676, GSE71989, GSE15471, GSE22780, and GSE16515) into a single data frame. The datasets with common genes were merged based on gene identifiers such as gene names or probes. Each microarray dataset was normalized using a robust multi-array average (RMA), and to perform differential expression analysis, we used logistic regression to classify samples based on gene expression using scikit-learn. Here, we took the condition (cancer vs normal) as the target variable and the expression levels as predictor variables. We also incorporated a random forest (RF) ML algorithm, a more versatile and robust supervised machine-learning technique for differential analysis. We incorporated the following steps for the analysis:

**Step I**: Transformed individual datasets using ${\text{X}}_{\text{iJ}}={\text{X}}_{\text{iJ}}\_\text{med}(\text{Xi})/\text{Iq}{\text{R}}_{\text{i}}$ to bring them all on similar scales, where ${\text{X}}_{\text{iJ}}$ represents the value of the expression that matches the ${\text{I}}^{\text{th}}$ genes and ${\text{J}}^{\text{th}}$ samples. Med (Xi) is the median expression of the ${\text{I}}^{\text{th}}$ gene; $\text{Iq}{\text{R}}_{\text{i}}$ is the interquartile range of the ${\text{I}}^{\text{th}}$ genes.

**Step II**: Generate a transformation matrix by merging all the healthy and control samples of all the datasets using their adjacent unique gene IDs.

**Step III**: Identify 60 % of the random samples as training sets and 40 % as the test datasets.

**Step IV**: For the ${\text{I}}^{\text{th}}$ generation, train the RF model and then classify for test datasets (I = 1,2, 3, g), where g is the total number of unique genes in the sets.

**Step V**: Extract the upregulated and the downregulated differential genes by putting the condition if (AUCi> 0.90, ACCi≥0.85, and log_2_FC ≥ 2) for upregulated, and (AUCi> 0.90, ACCi≥0.85, and log_2_FC ≤ −2) for the downregulated ones.

All the results were analyzed and visualized with libraries such as Seaborn and Matplotlib.pyplot using Python 3.0 packages.

### 2.3. Single-cell spatial transcriptome analysis and immune correlation analysis

We incorporated the Scanpy package to extract the single-cell 10X transcriptomics data. After filtration and removal of low-expressed genes, we preprocessed the data by removing the mitochondrial genes (having expressions greater than 25 % and genes with expression less than 200). We performed the dimensional reduction and downscaling using PCA and t-SNE to reduce the feature space. To identify the distinct cell population, we used UMAP-learn, matplotlib, and Seaborn using the Python 3.0 package. We annotated the cell clusters into normal and tumor tissues that expressed the same genes. We also used CIBERSORT to analyze immune cells to determine the level of infiltration in cancerous and non-cancerous diseases [11]. We also identified the correlation between the key genes and immunity using the TISIDB database [12].

### 2.4. Enrichment and pathway analysis

The key cDEGs were subjected to enrichment and pathway analysis at biological, cellular, and molecular levels using the GO terms by SHINYGO 0.82 and metascape. The primary idea was to understand the cellular functions and their molecular activity and to predict the locations where these genes perform their functions. The “Kyoto Encyclopedia of Genes and Genomes” (KEGG) pathway analysis tells us about the various metabolic pathways involved in the key genes. Fischer’s exact test was used with a P-value of ≤0.05 and count >3 to identify the significantly enriched terms.

### 2.5. Mutational, expression, and survival profiling

We tracked the top predictions of mutational frequencies using the cbioportal and TCGA databases. The cbioportal is a comprehensive genomic database that provides data for visualization, analysis, and download for large data chunks. To confirm the expression of the individual differential marker genes and understand the survival (disease-free and overall survival we used the gene expression profile interaction analysis (GEPAI2) [12,13], an interactive tool that uses custom statistical methods and puts a threshold on a given database to obtain DEGs and their chromosomal distribution.

### 2.6. Post-translational regulators prediction and non-coding RNA network construction

To explore the post-translational and transcriptional regulators of the predicted key cDEGs, we constructed a regulatory network of the non-coding RNAs with the genes and proteins using DIANA-Tarbase v8 and JASPAR. The significant non-coding miRNAs that leverage the key marker genes were constructed as a network using CytoScape version 3.10.3 [14] and NetworkAnalyst [15].

### 2.7. Compound selection, data filtering, and ADMET profiling

The current study retrieved ~420000 natural compounds from the COCONUT database (https://coconut.naturalproducts.net/) and ~8100 already approved FDA drugs from the database (https://catalog.data.gov/dataset/drugsfda-database). We also leveraged data using multiple high-standard datasets, including BindingDB [16] and ChEMBL [17]. We refined the data by removing duplicate values and entries. The COCONUT data encapsulates different classes of natural compounds, including alkaloids, terpenoids, shikimates, and phenylpropanoids. We procured the combined canonical and isomeric simplified molecular input line entry systems (SMILES) for each class of compounds. The compounds and drugs were filtered and checked for adsorption, distribution, metabolism, excretion, and toxicity using ProTox-II (https://tox.charite.de/protox3/) and DeepChem [18] (Python package for ADMET prediction), and SWISSADME (http://www.swissadme.ch/). We also evaluated the Lipinski rule of five. We screened only those compounds that did not exhibit any violations for further analysis.

### 2.8. Data augmentation, generation of Morgan fingerprints, and SELFIES

The dataset consisted of fingerprint-encoded molecular descriptors extracted using curated libraries of small molecules. The dataset consisted of Morgan fingerprints (FP_0 to FP_n) as molecular descriptors [19]. We used the Log-transformed IC50 values as the target variables. For QSAR predictions, we used the canonical SMILES to extract the molecular features, and we used SELFormer [20]. This novel transformer-based chemical language model utilizes the SELFIES notation as input.

### 2.9. Deep learning and machine learning models for screening

We employed machine learning models to predict inhibitory activities using molecular features. We assumed a binary classification (active vs inactive) or half maximal inhibitory concentration (IC_50_) modeling, depending on the availability of the data. Lacking extensively known activities for TNFRSF10A/TRAIL-1, specifically, we built the proxy training sets from relatable targets and general apoptosis inhibitor data from ChEMBL and BindingDB. Classical ML models [21] trained for QSAR included the XGBoost, Random Forest (ensemble tree-based models), support vector regression (SVR), gradient boosting, and Light GBM. We used the SELFormer, a custom transformer-based model that takes SELFIES token sequences as input for deep learning. The SELFormer model uses a BERT-like transformer encoder to process SELFIES and a regression head to output predicted IC_50._ We evaluated the model performance using regression metrics, including root mean square error (RMSE), Mean absolute error (MAE), and R^2^.

### 2.10. Model optimization, SHAP analysis, and hyperparameter tuning

The models were optimized using OPTUNA (an automated hyperparameter optimization framework) [22]. Optimize parameters such as tree depth in XGBoost, learning rate, and layer sizes in deep networks to maximize predictive accuracy. We performed the Shapley additive explanations (SHAPS) analysis of the tree models to interpret the importance of features, identifying which molecular descriptors most influenced activity predictions. We ran trials of hyperparameter tuning to determine the optimal combinations of the number of trees (N in estimators), learning rate, and maximum tree depth. Post-validation, we used the best-performing model to predict scores for the entire compound library.

### 2.11. Protein preparation, binding pocket predictions, and virtual screening

We retrieved the human 3D crystal structure of TNFRSF10A/TRAIL-1 from the RCSB protein data bank (PDB code: 5CIR). We performed the structure visualization using USCF Chimera and the MGL Tool. Chain D was preprocessed by removing the heteroatoms and used for further analysis per the study requirement. We removed the crystal-bound water molecules and other ligands before building the missing residues using MODELLER 9v15 [23]. We used CASTp 3.0 to identify the empty and favorable binding pockets, and to measure the geometric and topological properties of the protein structure [24]. We conducted the docking analysis using Autodock Vina [25] with GPU 2.0 acceleration using NVIDIA RTX 4090 graphics to calculate the binding energies and the number of interacting residues. Batch SMILES strings were processed using RDKit and AllChem packages using Python 3.0. All the SDF structures were first converted to the PDBQT format required for docking using Python. We visualized the output PDBQT files from post-docking analysis using BIOVIA Discovery Studio [26].

### 2.12. Stability dynamics analysis using MD simulations

We individually evaluated the top 100 predictions with the highest binding affinities using molecular dynamics (MD) simulations for a 100 ns run. Using the GROMACS 2023.1 package (https://manual.gromacs.org/2023/download.html), we applied the CHARMM36 forcefield for the protein and CGenFF (https://cgenff.com/) for ligand parameters (generated using PARACHEM). The only complex that showed stability for 100ns stretched beyond the MD run at a 300ns timescale. Trajectory analysis focused on the stability of the binding interaction. The energy was minimized at a rate of 20 kJ/mol/nm using the long descent steep algorithm for 20,000 steps, followed by 20,000 steps of conjugate gradient to avoid steric clashes. We performed the system equilibration with position restraints (NPT and NVT) at a constant temperature of 310K and a pressure of 1 bar for a 300 ns timescale. We computed the root mean square deviation (RMSD), root mean square fluctuations (RMSF), radius of gyration (Rg), number of H-bonds, and the interaction energy along with the individual plots. During MD simulations, we performed the principal component analysis (PCA) to explore the dominant motions and conformational dynamics. Solvent accessible surface area (SASA) quantifies the extent of protein surface accessible to solvent molecules and is a proxy for conformational exposure, compactness, and folding stability. We executed the molecular mechanism Poisson-Boltzmann Surface Area (MM/PBSA) calculations on snapshots from the 300ns MD trajectories. Binding free energy (Δ G_bind) was computed using the formula:

$$\text{Δ Gbind}={\text{G}}_{\text{complex}}-\left({\text{G}}_{\text{protein}}+{\text{G}}_{\text{ligand}}\right)$$

**Fig. 1.**
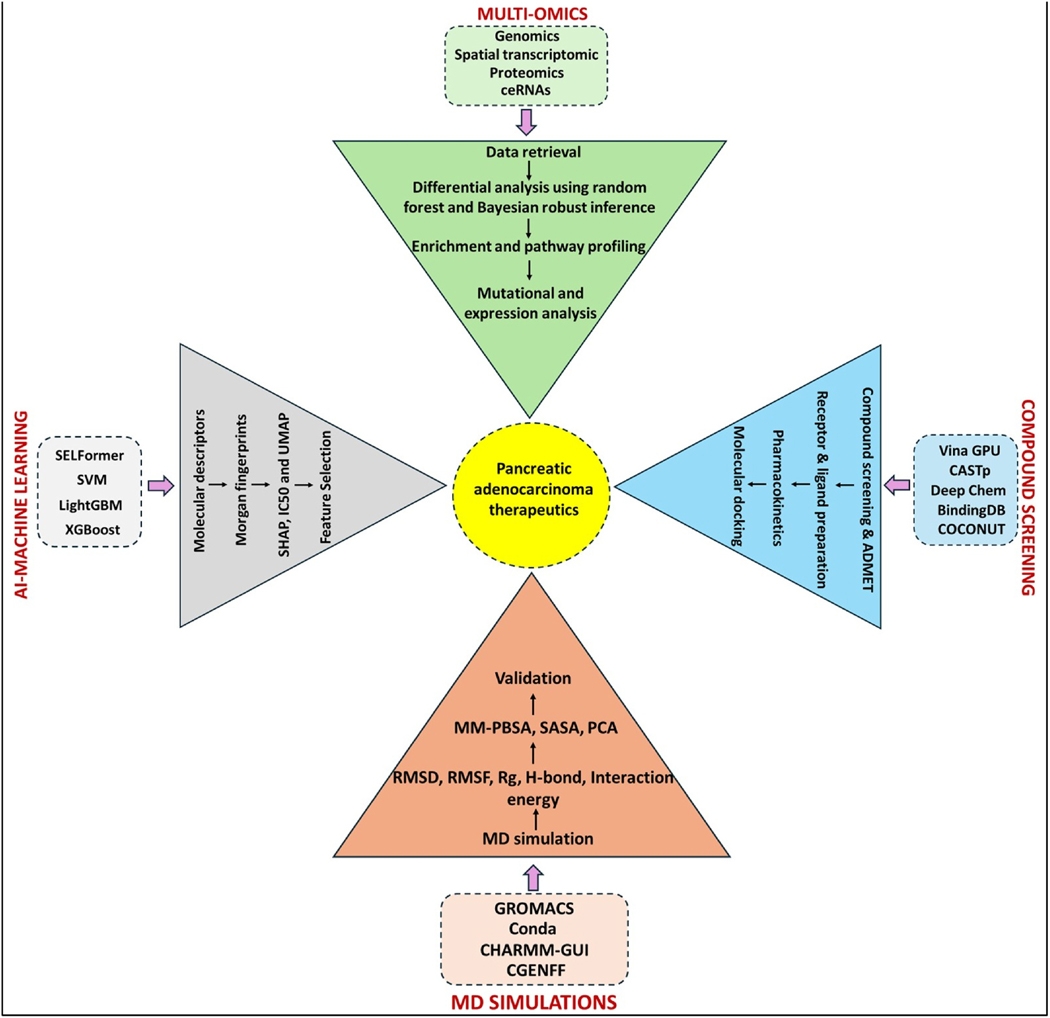
Representation of the research pipeline integrating multi-omics, artificial intelligence, machine learning, and simulation studies for therapeutic targeting in PDAC. The multi-omics arm incorporates datasets from various sources, including genomics, transcriptomics, proteomics, and competing endogenous RNA (ceRNA) networks. Data curation was followed by identifying differentially expressed marker genes using the Bayesian robust inference and random forest approach. Pathway and enrichment analysis revealed key metabolic pathways and disease-relevant biological processes, peaking in expression, mutational, and survival correlations for the predictions of highly relevant targets. We used FDA-approved and natural small-molecule databases like BindingDB, COCONUT, and DeepChem for Compound prediction and ADMET analysis. To predict the binding sites, we used the CASTp tool, and to calculate the binding affinities, we used the AutoDock Vina with GPU-acceleration. Further, artificial intelligence machine learning (AI-ML) transformed molecular representations (like canonical SMILES) into SELFIES, descriptors, and fingerprints. We predicted and evaluated the models using different ML algorithms like random forest, SVM, LightGBM, and XGBoost. Parallel to this, a transformer-based deep learning model (SELFormer) was deployed for quantitative structural activity relationship (QSAR) to predict the IC50 values. Model deployment prioritizes the potent candidates for drug repurposing and screening. Final validation was done by performing molecular dynamic simulations (MD simulations) using GROMACS to assess the stability of the complexes by analyzing the root mean square deviations (RMSD), root mean square fluctuations (RMSF), radius of gyration (Rg), number of the hydrogen bonds (H-bonds), and the minimum distance among the residues. Further, MM-PBSA, SASA, and PCA analyses were conducted to check the thermodynamic potential of the ligand interaction with the receptor.

Fig. 1 represents a detailed methodology pipeline.

## 3. Results

### 3.1. Raw data collection, preprocessing, normalization, and identification of cDEGs

**Fig. 2.**
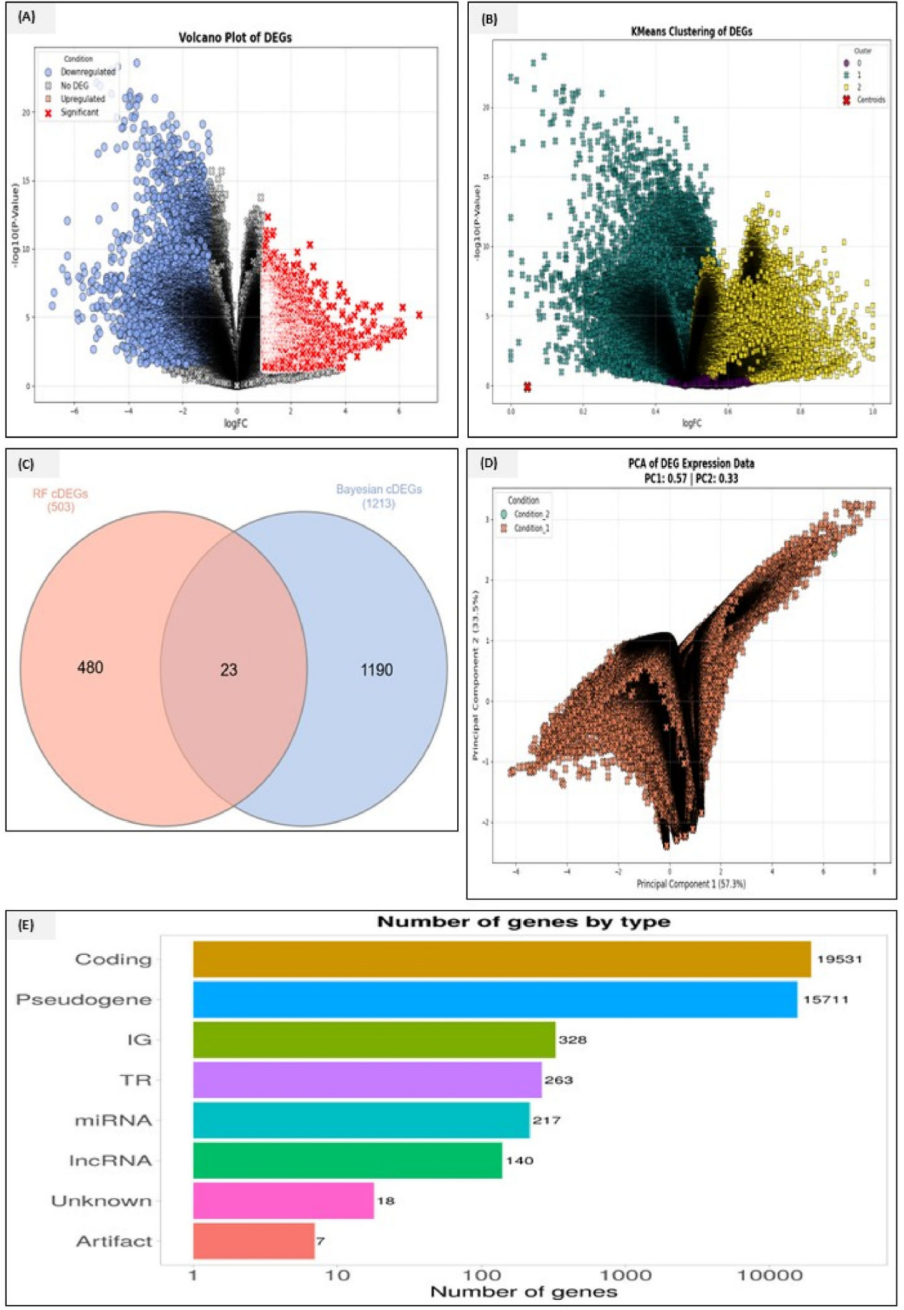
**(A**–**E):** Differential expression analysis and PCA of the gene expression data. (A) A Volcano plot of DEGs that visualizes the significance and the rate of gene expression changes between experimental conditions. The one highlighted in red represents the upregulated, the one highlighted in blue represents downregulations, and the one in grey represents significant changes. (B) K-means clustering of DEGs represents the results of clustering applied to the DEGs, grouping genes into distinct clusters based on the expression patterns. The clusters represent the degree of upregulation or downregulation. Cross1 represents the number of downregulated differential genes in the merged datasets, whereas the yellow square 2 represents upregulated ones, and the yellow centroid represents no DEGs. (C) After applying the random forest and Bayesian robust inference algorithms, the representation of overlapping DEGs across multiple gene expression datasets. 23 cDEGs were identified using the random forest and Bayesian robust inference. (D) PCA plot of the DEGs expression of data showing the variation between conditions based on the first two principal components (PC1 and PC2) in two conditions, upregulation and downregulation. (E) The bar chart categorizes genes based on their functional types (coding genes, pseudogenes, transcriptional regulators, miRNA, lncRNA, and others). The chart shows that the coding genes constitute the majority of DEGs, followed by pseudogenes and non-coding RNA types. (For interpretation of the references to color in this figure legend, the reader is referred to the Web version of this article.)

We retrieved the data from six different PDAC GEO datasets (GSE30134, GSE32676, GSE71989, GSE15471, GSE22780, and GSE16515) using statistically robust logistic regression and random forest machine learning algorithms to predict the differential marker genes between the normal control samples and PDAC samples. The number of gene counts initially present before merging in these six individual data sets was 45019 (GSE16515), 53687 (GSE 22780), 52119 (GSE15471), 54676 (GSE71789), 45119 (GSE32676), and 2947 (GSE30134), The merged data initially consisted of 208448 transcripts which consisted of duplicates and missing values. We obtained a single dataset after removing missing low-expressed transcripts with duplicate values. After data normalization, cleanup, and log transformations, we identified 22775 unique genes from the merged dataset. We set the cutoff values to log_2_Fc > 2 and log_2_Fc < −2 to identify the highly upregulated differential genes and separate the low-expressed downregulated ones (Fig. 2(A)). Using Bayesian robust inference and applying a cutoff of PP > 0.85, we detected 188 upregulated and 1025 downregulated DEGs with an overall count of 1213 DEGs. By applying random forest on the same merged data and then computing the ACC and AUC scores, we identified 501 cDEGs (377 upregulated and 125 downregulated genes. We obtained 23 cDEGs after plotting the Venn analysis, as shown in Fig. 2(C). For other related information, see Supplementary Information Fig. 1. We also analyzed the publicly available single-cell spatial transcriptomics data from GSE2020251, including the primary PDAC tumor samples profiled using a 10x Genomics Visium platform. The dataset provided spot-level gene expression profiles with spatial coordinates. 11 clusters were available during the data acquisition from single cell transcriptomics, out of which five appeared to be enriched and of the study interest. On the otherhand, spatial gene expression matrices were downloaded, log-normalized, and quality-filtered (minimizing the mitochondrial gene percentage and requiring sufficient unique genes per spot). Dimensionality reduction and clustering were applied to identify distinct tumor niches. Principal component analysis (PCA) was used to capture major variations (Fig. 2(D)), and the top principal components were used to compute UMAP embeddings. We also performed unsupervised clustering using the Leiden algorithm (with optimized resolution) and grouped spatial spots into microenvironment niches. Spatial expression maps of top differential markers were generated by plotting violin plots across different niche types. This enabled the identification of fibroblast subpopulations, immune cells within each cluster, spatial spots, and cluster-specific expression levels. To further elevate our study, proteomics data from the CPTAC PDAC proteomic data was extracted to find the importance of top predictions in tumor versus normal pancreatic tissues by mapping the expression at both genomic and proteomic levels, and how other proteins play an important role in the regulation of differential markers. In proteomics, the raw data files were extracted using the WGET package in Python and OpenMS for protein identification and Proteowizard libraries for protein quantification and differential expression analysis. This allowed us to conclude that the top differential markers upregulated at the genomic levels also show higher expression levels, even at the proteomics levels. All these plots can be seen in Fig. 2(A–E).

### 3.2. Functional enrichment and pathway evaluation of top differential picks

**Fig. 3.**
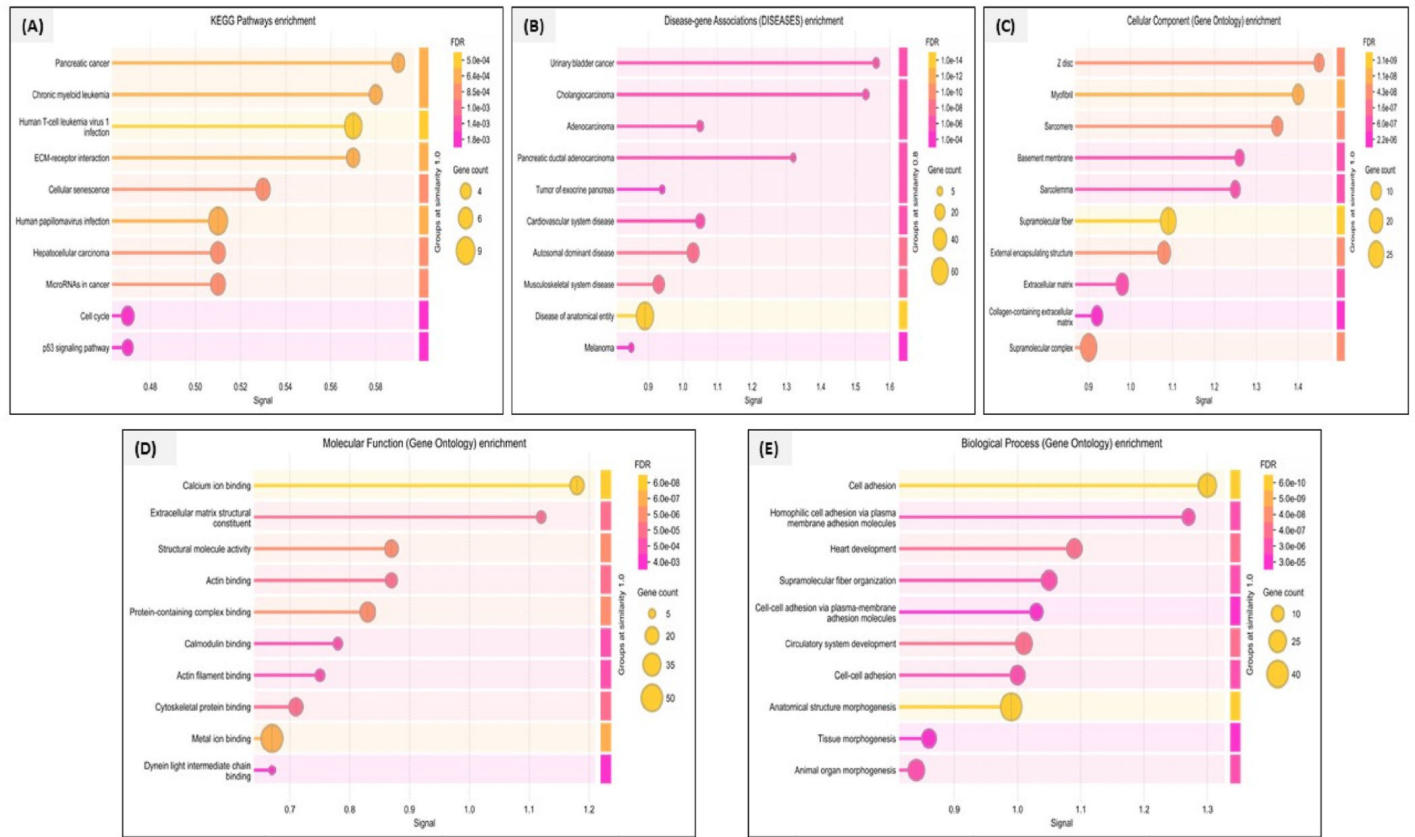
**(A**–**E).** Plots represent functional enrichments and KEGG pathway analysis. (A) Kyoto Encyclopedia of Genes and Genomes (KEGG) pathway plots show the enrichment of genes in various pathways, focusing specifically on pancreatic cancer. The X-axis represents the enrichment signal, while the color intensity represents the false discovery rate (FDR), with darker shades indicating more significant enrichment. Pathways such as pancreatic cancer, cell cycle, and chronic myeloid leukemia show more enrichment, suggesting a potential involvement in pancreatic tumorigenesis. (B) Disease-gene association enrichment (DISEASE). Plot showing the enrichment of genes associated with various diseases. Most of the genes were relatively involved in pancreatic ductal adenocarcinoma (PDAC), cholangiocarcinoma, and urinary bladder cancer. (C) Cellular component enrichment (GO_CC) shows that genes are predominantly enriched in forming structures like Z discs, basement membranes, and sarcomeres. These showed that most genes are involved in maintaining structural integrity and functions. (D) Molecular functions (GO_MF) showing the molecular functions enriched in pancreatic cancer-associated genes. Extracellular matrix structural constituents and calcium ion binding are the most enriched functions. Enriching action binding and cytoskeleton protein binding suggests a role in maintaining the cytoskeleton and promoting cellular motility during metastasis. (E) Biological Process (GO_BP) identifies key processes such as tissue morphogenesis and anatomical structure morphogenesis. Enrichment of circulatory system development further shows the role of blood vessel formation in tumor growth and spread. (For interpretation of the references to color in this figure legend, the reader is referred to the Web version of this article.)

Ontology enrichment analysis was performed after identifying common DEGs using SHINYGO 0.82 and metascape. The functional enrichment was done at three levels, i.e., cellular (GO_CC), biological (GO_BP), and molecular functions (GO_MF). From the enrichment, it was found that the top target predictions were involved in urinary bladder cancer, cholangiocarcinoma, and the diseases of the anatomical entity. These top marker differential targets were H6PD, DHRS3, TNFRSF10A, EXPH5, ARRDC1, FLVCR1, PASK, SH2D3C, COL4A4, EPHA2, JAG2, ZC3H7B and NCOA2. Regarding the enrichment at GO_CC, the top predictions involved forming Z-disc, myeloid formation, and sarcomere formation. On the other hand, when talking about GO_BP, most of the differential predictions were involved in cell adhesion, homophilic cell adhesion, and heart development. All these enrichments can be visualized in Fig. 3. During the KEGG pathway analysis, we identified that the top predictions involved pancreatic cancer, chronic myeloid leukemia, human T-cell leukemia virus, one infection, and ECM receptor interactions. All the enrichments and pathway analyses confirmed the direct or indirect involvement of marker target genes in PDAC. For detailed enrichments, **see** Supplementary Information Table 1.

### 3.3. Mutational analysis, expression, and survival profiling to confirm the target

**Fig. 4.**
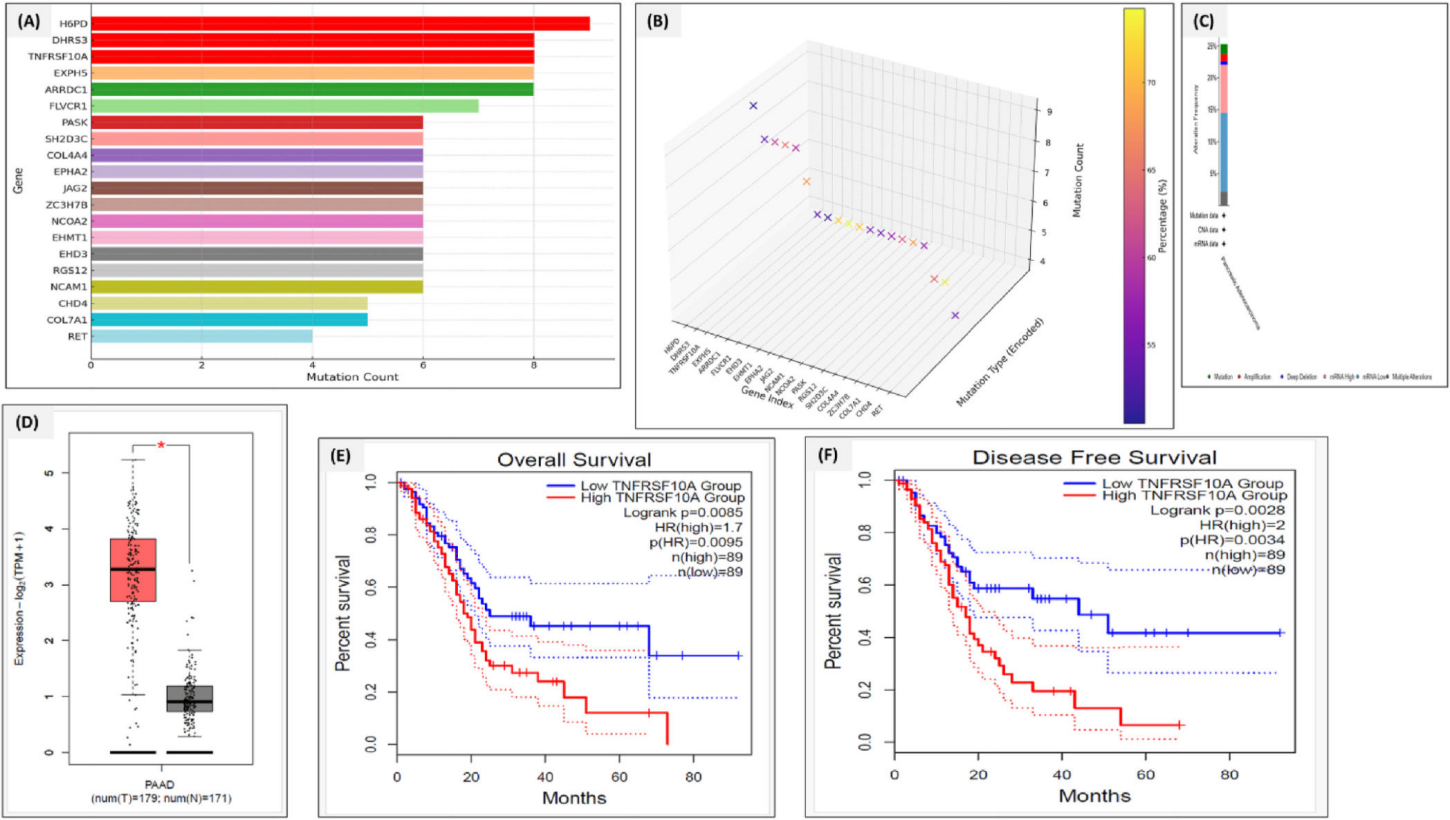
**(A**–**F).** Plot representation of the mutational analysis and (A) Mutational profiling and association of false discovery rate (FDR) in PDAC. The rows represent the individual genes, and the columns show the mutational frequencies. The color intensity shows the false discovery rate (FDR), with the darker colors having low FDR and being more statistically significant. Differential marker genes such as H6PD, DHRS3, and TNFRSF10A were shown to be highly mutated, suggesting their potential role in PDAC. The circle size shows the visual clue of which gene has a higher mutational burden than others. (B) 3D representation of the various mutational frequencies with H6PD showing the highest rate (9 %), DHRS3 (8 %), and TNFRSF10A (8 %). (C) Plot represents the categorization of the mutational counts and types, like point mutation, deletion, and mRNA upregulation. TNFRSF10A and H6PD showed multiple mutation types, such as amplification, missense mutation, and deep deletion. (D) Boxplot showing the expression of TNFRSF10A in tumor vs normal samples. (E & F) Plots representing the overall and disease-free survival of TNFRSF10A show that TNFRSF10A’s higher expression corresponds to low overall survival and disease-free survival. (For interpretation of the references to color in this figure legend, the reader is referred to the Web version of this article.)

We conducted a mutational analysis of the top 23 differential marker targets to strengthen our findings. The mutational frequencies were checked independently using the TCGA and GTEx databases for PDAC. The frequencies obtained during the analysis were H6PD (10 %), DHRS3 (9 %), TNFRSF10A (9 %), EXPH5 (8 %), ARRDC1 (8 %), FLVCR1 (8 %), and PASK (6 %). The observations can be visualized in Fig. 4(A–C). Most of the mutational signatures obtained were of missense and amplification types. From these findings, expression and survival analyses were also performed for these top marker differential genes to confirm a suitable target. Expression analysis was performed using GEPAI2 (a validated expression and survival analysis tool in different cancer types). TNFRSF10A was found to be the ideal candidate for targeting among all the other marker genes based on survival and expression analysis. The expression of TNFRSF10A was significantly higher than the others. All the observations can be visualized in Fig. 4(D–F).

Thus, from the above observation. i.e., high expression, mutational frequency, and significant association with poor survival, we chose TNFRSF10A/TRAILR1 as the most promising and clinically relevant therapeutic target for our further study in PDAC.

See Supplementary Information Figs. 1 and 2 for expressions and survival analysis of the other top differential markers.

### 3.4. Establishment of target-non-coding RNAs-protein regulatory axis in PDAC

We further established the role of different non-coding RNAs and their role in the target marker genes in PDAC. We targeted the suitable gene-ncRNA-protein axis and checked the DIANA-TARBASE v8 and JASPAR databases. TNFRSF10A interacted with several regulatory and oncogenic miRNAs, including has-miR-182, has-miR-155, has-miR-24, and has-miR-124, therefore showing more potential posttranscriptional repression in the PDAC microenvironment. Highly connected genes like COL1A1, EPHA2, and HPGD are shown to be targeted by multiple miRNAs, which suggests their central role in PDAC progression. These involvements suggest a broader inflammatory and apoptotic signaling network. Additionally, protein-protein interactions showed that TNFRSF10A binds with other critical signaling molecules like the POLR2E, EPHA2, and HPGD, suggesting a potential role in oncogenic signaling. The overall integration of the non-coding RNA regulation, transcription factor binding, and proteomic interaction data shows the importance of TNFRSF10A in PDAC. All the interactions are represented in tabular format in Table 1.

**Fig. 5.**
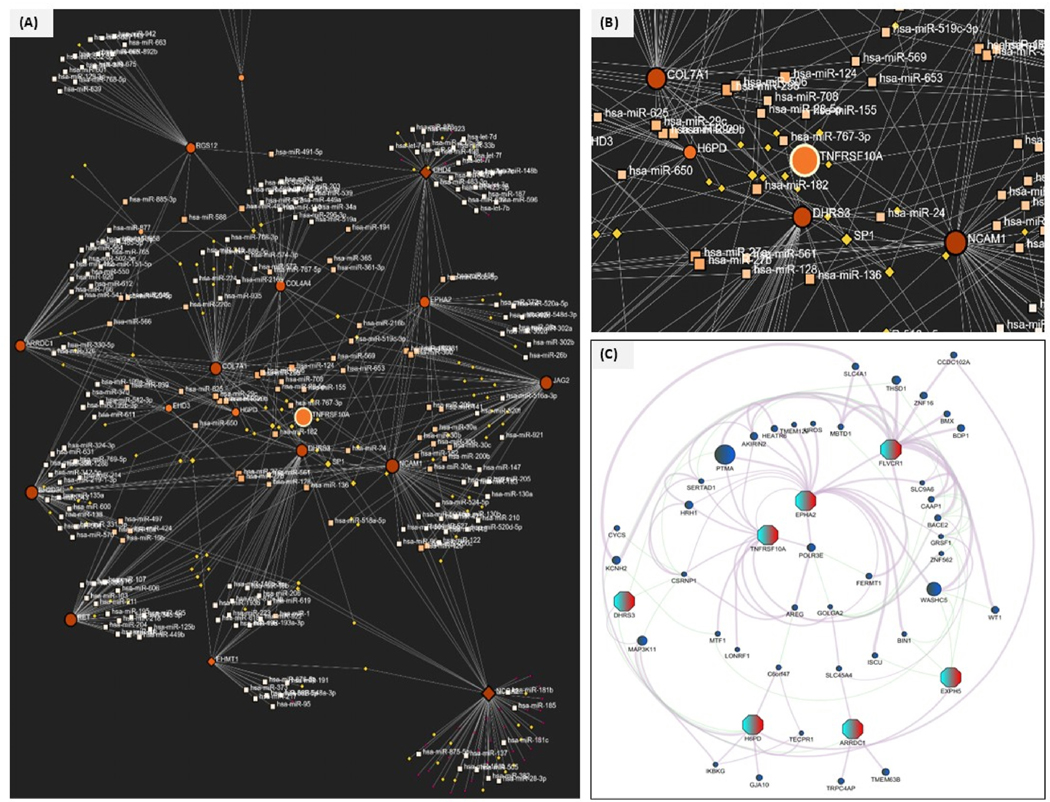
**(A**–**C): Representation of the integrated network of miRNAs, target genes, and proteins: (A)** Global miRNA regulatory network giving a comprehensive system-level map showing how differentially expressed target genes interact with their non-coding RNAs. Large orange circles represent the key target genes, the small white square represents the miRNA with its regulatory nodes, and yellow diamonds represent the transcriptional regulators based on the JASPAR database. Highly connected genes like TNFRSF10A, EPHA2, COL1A1, and HPGD show interactions with miR-182, miR-155, miR-124, and miR-24, suggesting their central regulatory role in PDAC progression. **(B)** Represents the network around TNFRSF10A and other key regulatory elements. has-miR-182, has-miR155, has-miR-24, and has-miR-124 are known to be oncogenic or tumor suppressive, and some of them are associated with chemoresistance, cell cycle progression, and epithelial to mesenchymal transition (EMT). Neighboring genes such as COL7A1, SP1, DJR53, and HPGD also interact with TNFRSF10A via protein-protein interaction or through co-targeting by miRNAs. **(C)** Merges omics data to define the comprehensive gene-ncRNA-protein regulatory axis. It shows the interaction of the key regulatory nodes with proteins, where large polygonal nodes (regulatory hub genes) are present. These are TNFRSF10A, EPHA2, FLVCR1, H6PD, ARRDC1, EXPH5 and DHRS3. Red denotes the upregulated proteins/genes, and the circular blue node represents downregulation. At the center are the TNFRSF10A, EPHA2, and POLR3E, which form a densely connected triangle that indicates a central role in PDAC progression. (For interpretation of the references to color in this figure legend, the reader is referred to the Web version of this article.)

Also, these miRNAs showed relevance and interconnections, and the focus was to identify the potential post-translational regulatory mechanisms and key regulatory hubs with a high degree of connectivity that are potentially crucial in disease mechanisms for PDAC. All these networks can be visualized in Fig. 5(A–C).

All the other relevant interactions can be seen in Supplementary Information Fig. 3.

### 3.5. TNFRSF10A is enriched in CAF-rich, hypoxic, and immunosuppressive niches of PDAC tumors

**Fig. 6.**
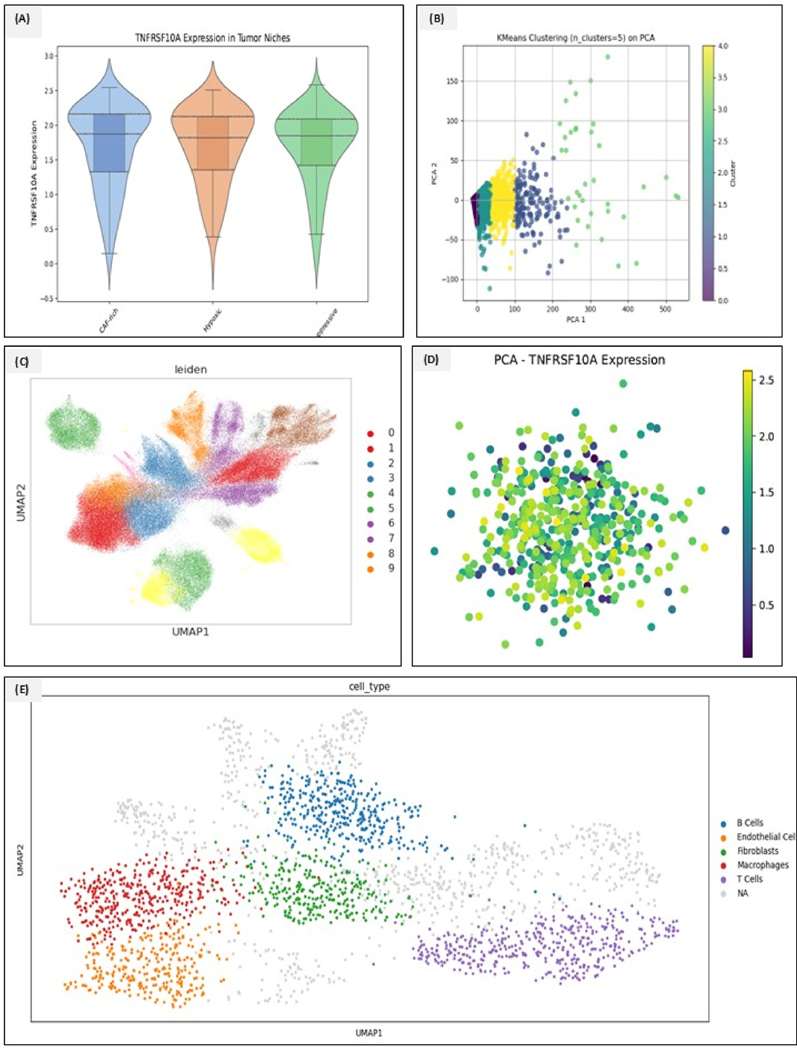
**(A**–**E):** Tumor necrosis factor receptor superfamily member 10A (TNFRSF10A) expression and tumor microenvironment analysis. (A) TNFRSF10A expression across niches shows the violin plots that compare the expression levels of TNFRSF10A in three distinct tumor microenvironments: carcinoma, stromal, and immune niches. The plot reveals that TNFRSF10A is most highly expressed in pancreatic carcinoma tissues, suggesting its potential role in tumor progression or response to therapy. (B) The plot for K-means clustering of PCA identifies subpopulations within the dataset, as represented by different colors. The clear segregation of clusters on the first two principal components, PC1 and PC2 expression patterns, can effectively differentiate tumor subtypes or cellular states. (C) UMAP visualization of the Leiden clustering plot illustrates the clustering of cells based on Leiden clustering, with each cluster color-coded. The distribution of cells across the UMAP space reflects the heterogeneity of TNFRSF10A expression across different cell populations, providing insights into the spatial and molecular organization of the tumor microenvironment. (D) PCA plot of TNFRSF10A expression represents the intensity of TNFRSF10A expression, with higher expression values clustered together, suggesting PCA can capture the main source of variability in the dataset related to TNFRSF10A expression. (For interpretation of the references to color in this figure legend, the reader is referred to the Web version of this article.)

Spatial transcriptomics data from single-cell RNA analysis revealed striking intratumoral heterogeneity in TNFRSF10A expression. Clustering of spatial gene expression data identified prominent niches with PDAC sections; **i)** CAF-rich stroma, located at the tumor-stromal interface with high fibroblast markers (ACTA2, FAP, COL1A1); **ii)** Hypoxic tumor cores, often proximal to necrotic regions and marked by upregulation of hypoxia-inducible genes like CA9, and VEGFA; and **iii)** Immune suppressive margins, characterized by accumulation of macrophages and regulatory T-cell signals (e.g., high CD163, TGFB1, low cytotoxic T-cell presence). TNFRSF10A expression varied significantly across these niches. In CAF-rich regions, TNFRSF10A mRNA levels were two to three times higher than in immune-suppressive regions (p = 0.004), and hypoxic tumor zones also showed elevated TNFRSF10A (1.8-fold above immune-suppressive regions). Contrastingly, regions with abundant lymphocytic infiltrates and less stroma exhibited lower TNFRSF10A. Dimensional reduction plots further illustrated these differences; on the PCA biplot of all the spatial spots, the first two principal components separated spots by microenvironment context and showed TNFRSF10A loading strongly on the element associated with CAF/hypoxia-rich spots. UMAP visualization recapitulates this, clustering TNFRSF10A-high spots distinct from TNFRSF10A-low, immune-rich spots. Leiden clustering partitioned the spatial transcriptomics into seven clusters, of which two clusters corresponded to TNFRSF10A high niches (CAF/hypoxia) and had minimal overlap with the cluster representing immune-suppressive, fibrotic-poor regions. These findings reveal that TNFRSF10A might be a feature of PDAC microenvironment states, probably those with abundant stroma and poor immune penetration. Consistent with the spatial data, TNFRSF10A transcripts were enriched in malignant cell subpopulations colocalized with myofibroblasts and in a subset of stromal myofibroblasts themselves. In contrast, endothelial cells and T-cells showed little TNFRSF10A. Interestingly, one of the fibroblast clusters expressed high alpha-SMA, and interleukin-6 showed moderate TNFRSF10A, raising the possibility that some CAFs in the TME express this death receptor, potentially affecting their survival or signaling. TNFRSF10A was largely absent in the immune clusters, supporting the notion that its high expression marks regions of immune exclusion rather than immune engagement. Our spatial and single-cell analysis maps TNFRSF10A to desmoplastic, hypoxia, and immune-suppressed compartments of PDAC tumors, implicating it in the biology of these aggressive niches. All the results can be visualized in Fig. 6(A–E).

### 3.6. Machine learning model run and evaluation

For the current study, we employed classical machine learning models using Morgan fingerprints, molecular descriptors, and five different ML algorithms (i.e., XGBoost, Random Forest, support vector regression (SVR), gradient boosting, and lightGBM). These models were built with five-fold cross-validation along with external test set validations. The models developed using Morgan’s fingerprints had better sensitivity for every ML algorithm. We, therefore, built a predictive model to evaluate compound activity against TNFRSF10A by extracting the data from the BindingDB, COCONUT library, and FDA-approved compounds. Using a curated training set for 13,201 compounds that included the analogs of known TRAIL pathways modulators and small molecules from the literature that sensitize or inhibit the TNFRSF10A signaling, we trained an ensemble QSAR model. All three metrics, i.e., R^2^ (coefficient of determination), MSE (mean square error), and MAE (mean absolute error), were independently calculated for all five ML algorithms. Random forest gave the highest R^2^ score of 0.65, an RMSE of 1.35, and an MAE of 0.82, followed by XGBoost and other models. SHAP analysis provided insight into these features, like the compounds with moderate lipophilicity (Log P ~ 3–5), and the presence of certain functional groups, such as tertiary amines and aromatic nitriles, tended to be predicted as active. On the other hand, molecules that were too polar or bulky were less favored, which illustrates that several properties like LogP, H-bond donors (HBD), and specific fragment fingerprints contribute most to active predictions. We also performed 20 trials of hyperparameter tuning using Optuna to determine the optimal combination of the number of trees (N estimator) [100,1000], learning rate, [1e-5, 1e-1], and maximum tree depth (max depth), [3,12]. A comparative analysis can be seen in Table 2.

**Fig. 7.**
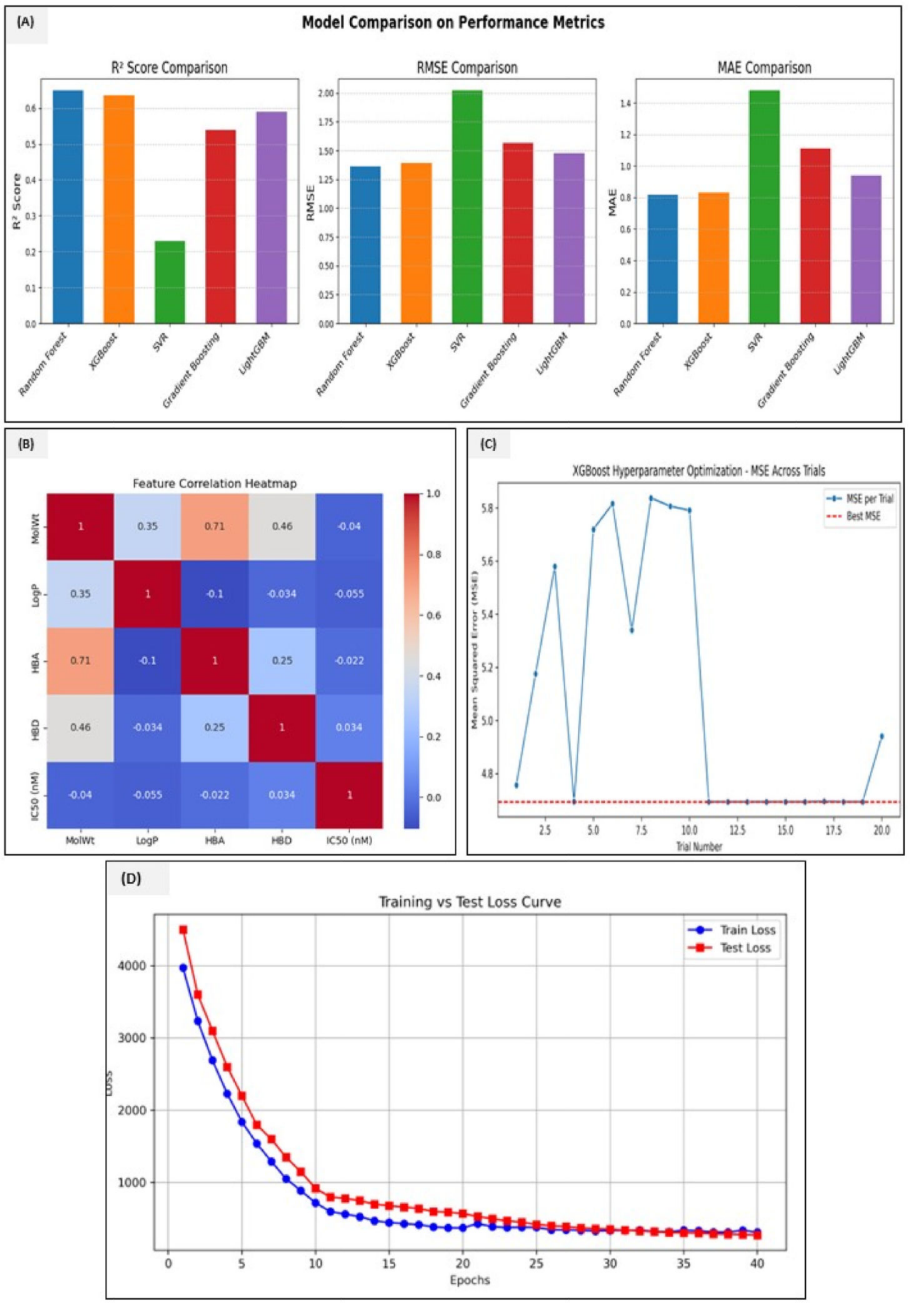
**(A**–**D):** Representation of the model comparisons and the hyperparameter optimization. (A) Plots represent the metrics comparison across models (random forest, gradient boosting, XGBoost, SVM, and lightGBM) based on the key evaluation metrics: R2, MAE, and RMSE. Random forest showed the best performance in terms of R2, highlighting its strong potential to explain variance. On the otherhand, XGBoost and gradient boosting outperform others in minimizing MAE and RMSE, suggesting that these models offer better precision and smaller prediction errors. (B) The heatmap displays the pairwise correlation coefficients between MolWt, HBA, HBD, LogP, and IC50 features. The strong positive correlation between MolWt and HBA and between HBA and HBD suggests these features carry similar output. (C) XGBoost hyperparameter tuning shows the mean squared error (MAE) across multiple hyperparameter optimization trials for the XGBoost model. The variation in MSE values across trials illustrates the sensitivity of XGBoost to hyperparameter settings. The dashed red line marks the best MSE achieved across all trials. (D) This curve compares the training and test loss during model training epochs. The training loss decreased steadily, while the test loss began to plateau and was consistently higher than the training loss. (For interpretation of the references to color in this figure legend, the reader is referred to the Web version of this article.)

Based on the calculations and model performance, we choose the best two models for further comparison with deep learning from SELFormer-transformation-based modeling. A comparative analysis of all the models is represented in Fig. 7(A–D). To compare the scores, see Supplementary Information in Table 2.

### 3.7. SELFormer-transformer-based deep learning and evaluation

**Fig. 8.**
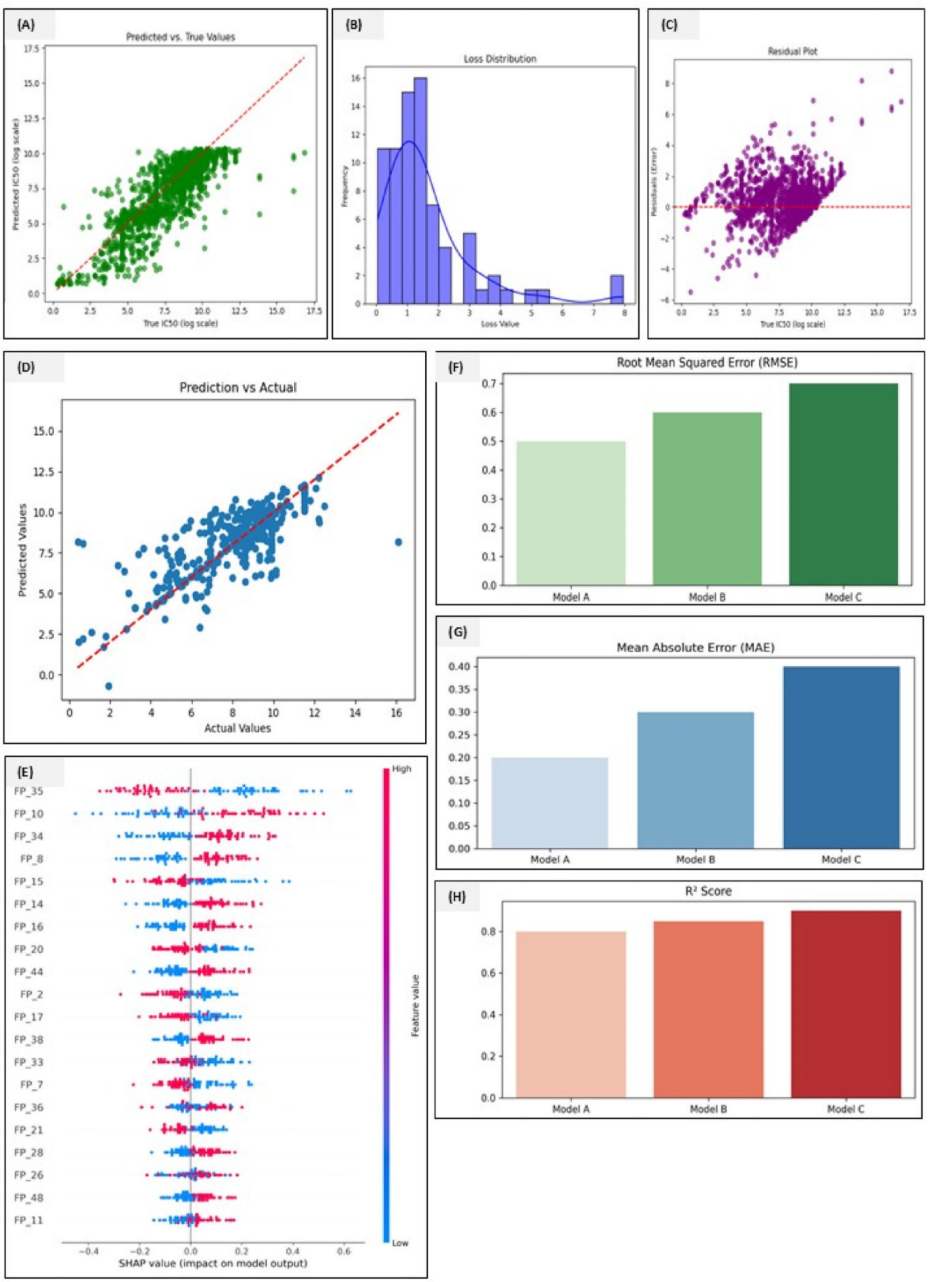
**(A**–**H):** Plots representing the overall performance evaluation of the predictive models for IC50 values using the SELFormer-transformer-based deep learning and other comparative modes, XGBoost, and random forest. (A) Predicted versus true value (Log IC50), where the scatter plot represents the relationship between the expected and actual IC50 values on a logarithmic scale. The predicted and the actual data points closely align with the red line, suggesting a robust linear correlation between predicted and true values, showing high prediction accuracy. (B) Histogram plot showing the distribution of model loss values during the training, with the y-axis representing the frequency and the x-axis showing the magnitude of the loss function. This tells us that the model achieved low prediction error for most observations. (C) The residual plot shows the residuals (the difference between the predicted and actual values) against the true IC50 value. Residues appear to show homogeneity of variance, establishing the accuracy of model assumptions. (D) Prediction versus actual values shows a continuous correlation between the model prediction and exact outcomes. (E) Shapley additive explanations (SHAP) feature importance plot shows the impact of individual features on the model output by calculating the SHAPY values for each feature. This analysis revealed the importance of features FP_10 and FP_35 in driving the model’s predictions of IC50. (F) Represents the root mean square error (RMSE) comparison of model A (SELFormer-based deep learning), model B (random forest), and model C (XGBoost). Model A showed the lowest RMSE and was performing the best of all the other two models chosen the best from the classical machine learning run. (G) Mean absolute error (MAE) comparison where Model A showed the best performance based on the lowest MAE score compared to Model B and Model C. (H) Bar plots compared the R2 scores across Model A, B, and C. All three models showed R2 scores above 0.8, indicating that the models explain a substantial proportion of variance in IC50 values. (For interpretation of the references to color in this figure legend, the reader is referred to the Web version of this article.)

We first evaluated the QSAR models trained to predict TNFRSF10A inhibitory activity. The SELFormer model, fine-tuned for IC50 predictions, was integrated with QSAR methods in which SELFIES-based molecular encoding ensured robust chemical representations. The self-attention layers in the SELFormer allowed efficient feature extraction by learning molecular interactions at different hierarchical levels. The deep learning models outperformed classical methods. The SELFormer model achieved the best results, with MAE = 0.9117, RMSE = 1.2731, and R^2^ = 0.6964, outperforming the random forest and XGBoost models. SHAP analysis revealed IC50, MolWt, and LogP as the key predictors. A comparative study of the scores is given in Fig. 8. In classification terms, the SELFormer attained an AUC>0.90 for capturing complex structure-activity relationships even in the low-data regime. It shows the loss distribution, predicted vs true values, and residual plots. It depicts the loss distribution and shows how the model prediction errors are distributed. The right-skewed distribution means most predictions have low errors, but there are some high-error outliers. Most errors fall between 0 and 2, indicating good predictive performance. Evaluates the agreement between predicted and experimental IC50 values. The points closely aligned with the Y = X reference line indicate strong model agreement, whereas some deviations suggest minor model biases, possibly in underrepresented chemical subspaces. This shows the validity of the SELFormer-based deep learning for predicting the TNFRSF10A inhibitors. Represents the residuals randomly distributed around zero, meaning the model does not have systematic biases. Also, some high error predictions suggest chemical diversity in the datasets. On the other hand, the figure also shows that the model improves over training epochs. From the observation, training and test loss decreased steadily, converging around 35 epochs. The test loss curve follows the training loss closely, indicating minimal overfitting. Feature importance analysis of the tree-based model via SHAP revealed that molecular weight, aromatic ring count, and the presence of certain functional groups, such as the indole or steroid-like scaffolds, were influential features correlated with predicted activity. This aligns with the observation that many known TNF family inhibitors are polyaromatic small molecules.

### 3.8. Structure analysis and docking validations of the predicted compounds

**Fig. 9.**
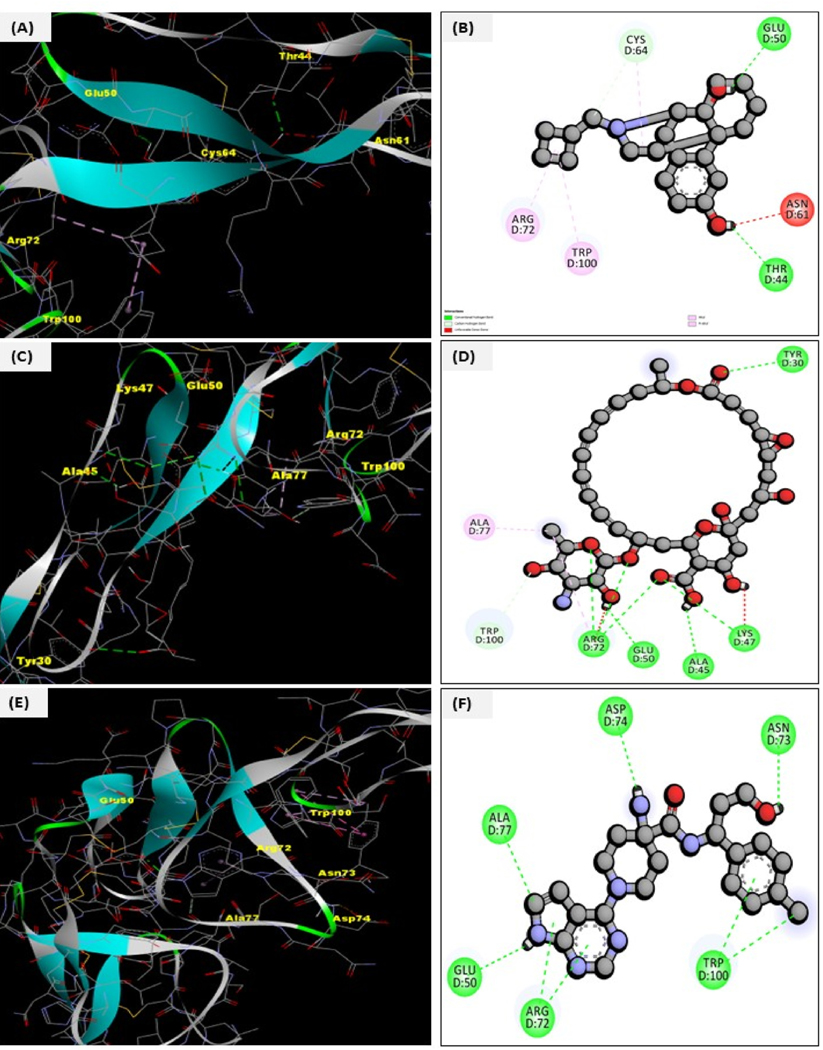
**(A**–**F):** Representation of the 2D and 3D docking poses of different complexes: TNFRSF10A-Temsirolimus (Complex I), TNFRSF10A-Ergotamine (Complex II), TNFRSF10A-Capivasertib (Complex III). Fig. 9(A&B) shows the binding poses of Complex I in 2D and 3D. The interacting residues were Glu50, Asn61, Thr44, Trp100, Arg72, and Cys64 with a binding affinity of −10.1 kcal/mol; Fig. 9(C&D) Complex II showed the interactions with Tyr30, Lys47, Ala45, Glu50, Agr72, Trp100, and Ala77 with an affinity of −9.6 kcal/mol. Fig. 9(E&F) Complex III represents the interactions with the residues Asp74, Asn73, Trp100, Arg72, Glu50, and Ala77 with a binding energy of −9.1 kcal/mol.

In parallel to the ML, we performed the docking analysis of the top predictions using GPU-accelerated AUTODOCK Vina. We calculated the box coordinates, center, and size for docking using the Python libraries, i.e., BioPython and RDKit. From the compound library (natural compounds + FDA approved) that was screened using Lipinski and ADMET analysis and included the top prediction from SELFormer and ML, we performed the docking analysis of the 25000 compounds. The TNFRSF10A receptor crystal structure was initially retrieved from the RSCB PDB using the structure ID: **5CIR**. The structure was initially composed of seven chains (A-G). We used the CASTp tool to predict empty binding pockets and found that ChainD was ideal for our study. The crystal structure was evaluated, and the missing residues were filled by homology modeling using MODELLER. The structure was then assessed by constructing a Ramachandran plot that showed the distribution of backbone dihedral angles (psi and phi), with the dense regions showing the preferred conformational states. For visualization, see Supplementary Information Fig. 3. Heteroatoms and extra water were removed before performing the docking analysis. The top predictions that showed the highest binding affinities were ergotamine, temsirolimus, and capivasertib. Compound ergotamine showed a higher binding energy, i.e., −9.6 kcal/mol, with an RMSD of 1.28 Å, consistent with the docking poses. Temsirolimus showed a higher binding affinity of −10.1 kcal/mol with TNFRSF10A and an RMSD of 1.39 Å, suggestive of higher binding interaction but with slightly higher deviation in structure during binding. On the otherhand, capivasertib showed a binding affinity of −9 kcal/mol with TNFRSF10A with an RMSD of 1.45 Å, showing a secure but less defined binding confirmation. The residue interactions were consistent with the predicted binding sites in all three complexes. The major common residue interactions were Glu50, Arg72, Ala77, and Trp100. This showed the higher binding of these top three predictions with the TNFRSF10A. In summary, docking analysis contributed that ergotamine, capivasertib, and temsirolimus have stronger binding potentials that point towards a higher inhibitory potential among all other natural and FDA-approved compounds. For binding affinities of the top 500 docked compounds, see Supplementary Information Table 3. The complexes’ binding can be visualized in Fig. 9 (A–F).

The interactions concerning temsirolimus, ergotamine, and capivasertib can be visualized in Table 3.

### 3.9. Molecular dynamics simulations to divine the stability of complexes

**Fig. 10.**
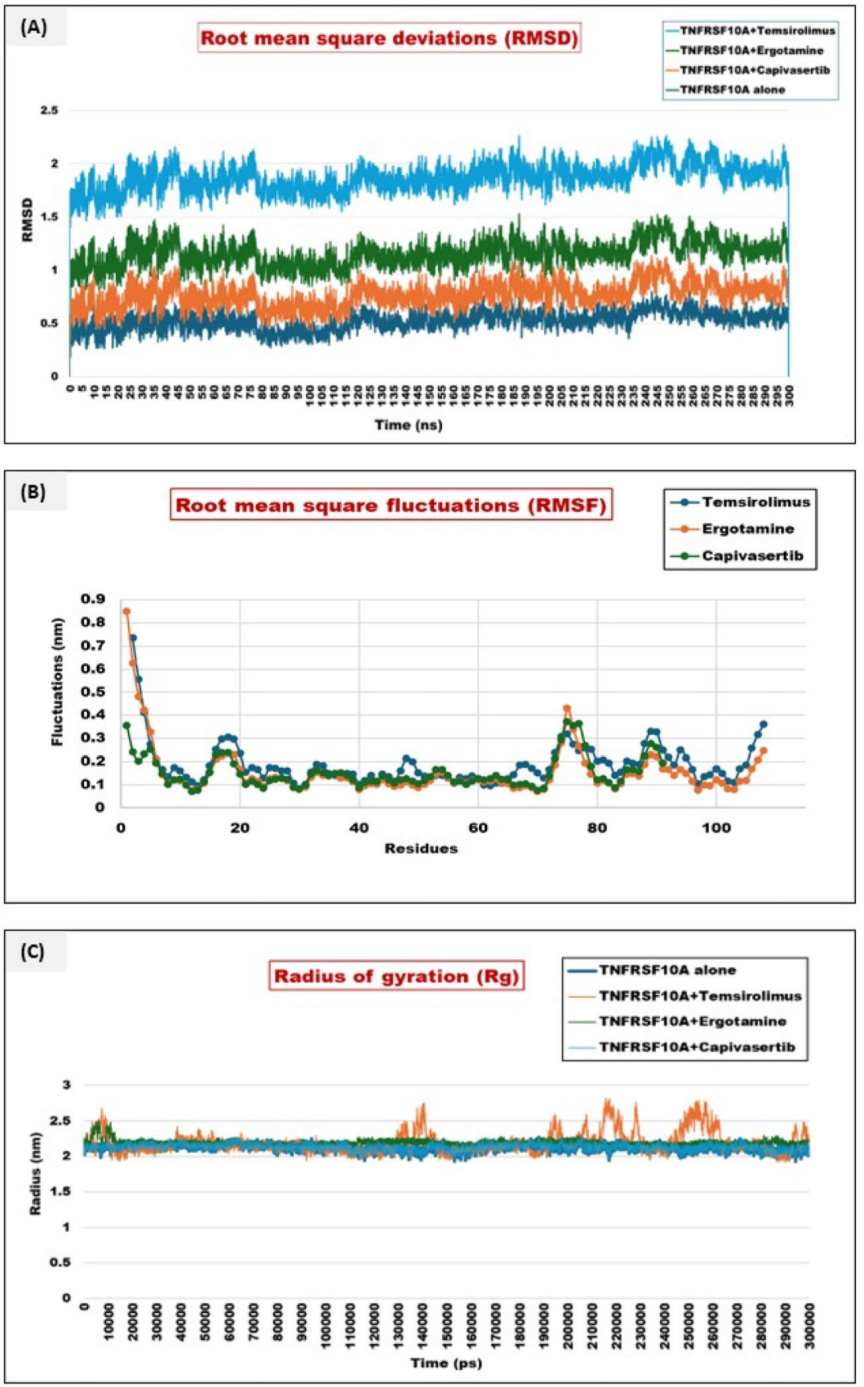
**(A**–**C):** Representation of the root mean square deviation (RMSD), root mean square fluctuation (RMSF), and radius of gyration (Rg). (A) RMSD of TNFRSF10A (backbone) alone, TNFRSF10A-Temsirolimus (Complex I), TNFRSF10A-ergotamine (Complex II), and TNFRSF10A-capivasertib (Complex III) was calculated and plotted for a 300 ns run. The Y-axis represents the root mean square deviation in nm, and the X-axis represents the time in nanoseconds (ns). The backbone alone gave the lowest variance and range in RMSD, suggesting its stability. On the otherhand, complex I showed the highest range, but it was stable throughout the run. Complex II was moderately deviated, and Complex III showed the least deviation. (B) RMSF was calculated to understand the flexible regions and how the binding affects the protein stability. The RMSF of the backbone alone was lower in most secondary structural elements. In contrast, complex I showed elevated RMSF in certain loop regions near the binding sites, and complex II showed lower RMSF than complex I. In contrast, complex III suggested that the binding of capivasertib stabilized certain areas of the backbone with reduced fluctuations. (C) Rg represents the overall compactness of the TNFRSF10A with different complexes. Rg of other complexes was also calculated, and the stability was noted for each complex. Complex I was found to show the least fluctuation among all the other Complexes.

The study implemented MD simulations to understand the atomic changes of a partially or as a whole macromolecule over a projected timescale of 300ns under certain physiological conditions. This analysis allowed us to evaluate the stability and the pattern of interactions between the protein-ligand complexes. It also allowed us to elucidate the extent of conformational changes a macromolecule shows in various hydrophilic conditions. A chunk of properties at structural levels was analyzed that consisted of global dynamic attributes such as root mean square fluctuations (RMSF), the root mean square deviation (RMSD), the radius of gyrations (Rg), interaction energy (Coulombs and Lennard Jones (LJ), number of hydrogen bonds formed, minimum distance between the residues and the MM/PBSA to calculate the binding free energy of the complexes [27]. The unbound TNFRSF10A exhibited the lowest RMSD in the 0.4–0.6 nm range throughout the simulation. This indicates a stable folding of the protein TNFRSF10A without ligands, with minimal global conformational changes once the equilibrium was achieved. The TNFRSF10A-temsirolimus (**Complex I**) displayed the highest RMSD range, fluctuating between 1.5 and 2.2nm. This elevation in RMSD shows that this complex induces more significant structural rearrangements. Still, despite higher fluctuations, the RMSD trend reached a plateau after the initial ~50 ns, indicating eventual stabilization with time. The RMSD in the case of TNFRSF10A-Ergotamine (**Complex II**) remained in a moderate range (1.0–1.5 nm). There were fewer fluctuations compared to temsirolimus, suggesting a more moderate yet stable rearrangement of the protein structures. In the case of TNFRSF10A-capivasertib (**Complex III**), the complex showed moderate RMSD values, slightly higher than ergotamine on average but lower than temsirolimus [28]. For the analysis of the flexible regions of TNFRSF10A and how the ligand binding modulates local dynamics, we calculated the RMSF for each residue across all simulation frames. TNFRSF10A alone exhibited relatively lower RMSF values (<0.15 nm) in most secondary structural elements, indicating a stable confirmation. Complex I showed elevated RMSF in certain loop regions near the binding sites, suggesting temsirolimus binding induced additional local rearrangements. Complex II showed lower RMSF values than Complex I, indicating that the binding pocket region showed modest fluctuations, indicating a more stable but dynamic binding mode. Complex III RMSF suggested that the binding of capivasertib stabilized certain areas of the receptors with reduced local volatility. The Rg showing the overall compactness of TNFRSF10A with different complexes was also calculated. Unbound TNFRSF10A maintained a relatively constant Rg, showing a stable fold in the absence of the ligand. Both complexes II and III displayed a slight increase in Rg (~2.1–2.2 nm) early in the simulation, suggesting a transient expansion of the protein as it accommodated the ligand. On the other hand, Complex I displayed an increase in Rg early in the run, suggesting a transient expansion of the protein in proximity to the ligand, but it stabilized eventually. This can be visualized in Fig. 10 (A–C).

The H-bond analysis revealed that temsirolimus exhibited the highest number of H-bonds throughout the simulation, reaching up to 4 or 5 concurrent H-bonds. The lower number of consistent H-bonds may suggest higher RMSD fluctuations. The ergotamine hydrogen bonding pattern was intermediate, with 1–5 H-bonds at various time points. The H-bond occupancy was less compared to capivasertib but more frequent than temsirolimus. This indicates a balanced combination of polar and non-polar interactions. The interaction energy of each complex was calculated, and it was found that temsirolimus showed the most favorable interaction energy in the range −1075000 kJ/mol. Regarding coulombic energy, ergotamine (−1071000 kJ/mol) showed moderate binding energy. Capivasertib (−1070000 kJ/mol) exhibited intermediate to less favorable binding energy. The energies are hereby represented in Table 4.

**Fig. 11.**
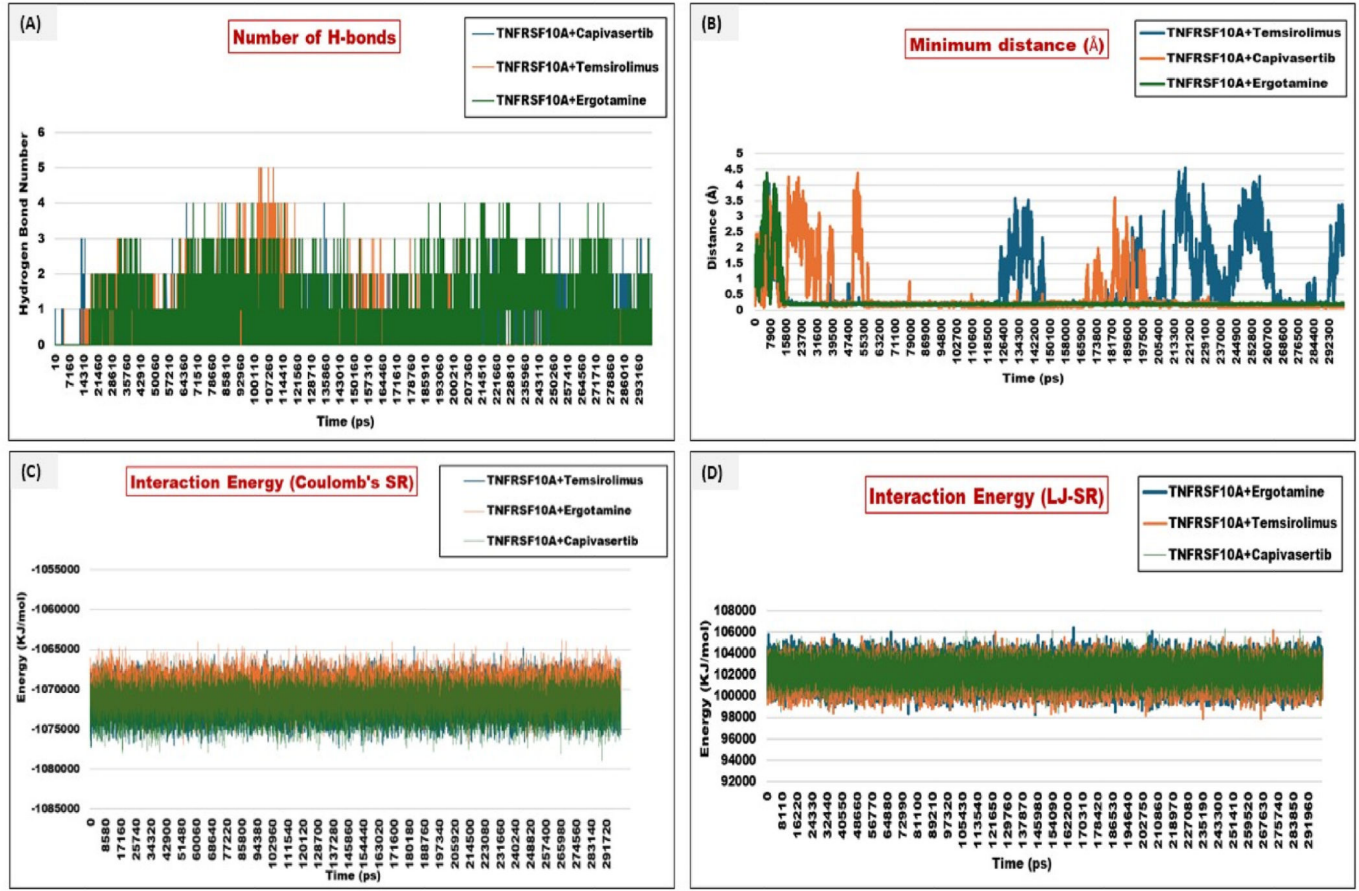
**(A**–**D):** Plot representing the number of hydrogen bonds formed (H-bonds), the minimum distance between the interacting residues (angstrom), interaction energy in Coulomb’s–single residue (Coulombs-SR) and Lennard-Jones (LJ-SR). (A) H-bond plots revealed that Complex I (TNFRSF10A-Temsirolimus) formed the highest number of hydrogen bonds. In contrast, Complex II (TNFRSF10A-Ergotamine) formed an intermediate number of hydrogen bonds, and Complex III (TNFRSF10A-Capivasertib) formed an intermediate number of hydrogen bonds. (B) Minimum distance analysis of temsirolimus, ergotamine, and capivasertib with various segments of TNFRSF10A in angstrom. (C) Interaction energy represents the overall binding ability of individual ligands with receptors. Complex I showed the highest energy at −1075000 kJ/mol, Complex II represented a value of −1071000 kJ/mol, and Complex III showed a range of −107000 kJ/mol. The LJ-SR represented energy in positive forms. Complex I showed more positive energy, indicating its structural integrity compared to Complex II, which had a moderate value, and Complex III, which had an intermediate value compared to Complex I and II.

The greater the values are in negative terms in the case of Coulomb’s energy and the more positive in the case of Lennard Jones, the more favorable binding and stable complexes are suggested. This can be visualized in plots in Fig. 11(A–D).

### 3.10. PCA, SASA, and MM/PBSA to predict the binding free energy of TNFRSF10A complexes

**Fig. 12.**
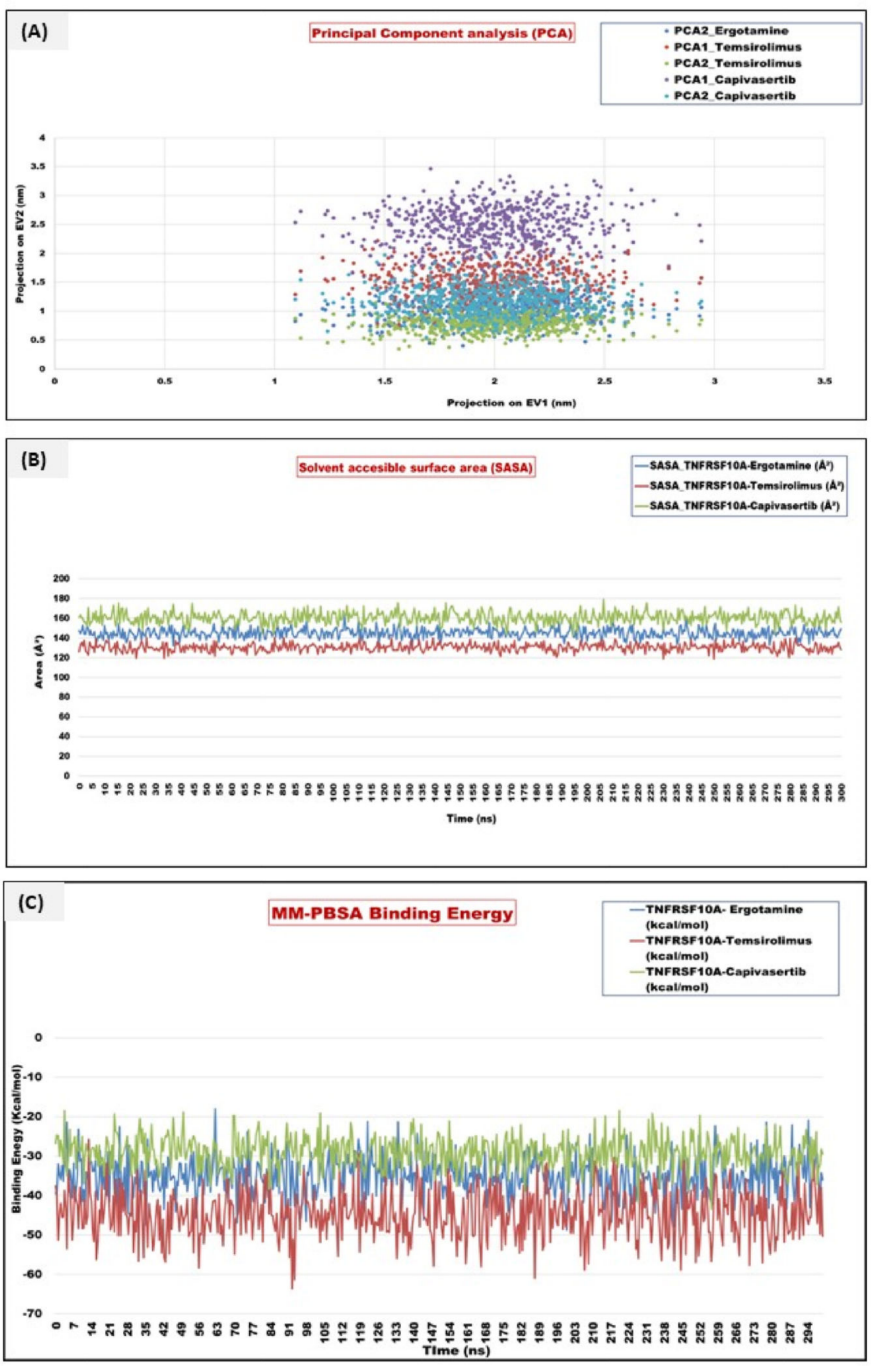
**(A**–**C):** Representation of the Principal component analysis (PCA), Solvent accessible surface area (SASA), and MM-PBSA. (A) PCA analysis for the top bindings with TNFRSF10A: The principal components, PC1 and PC2, show the conformational fluctuations during the binding of TNFRSF10A with temsirolimus, ergotamine, and capivasertib. TNFRSF10A-Temsirolimus (Complex I) showed the least conformational transitions during the 300 ns simulation run. TNFRSF10A-Ergotamine (Complex II) showed moderate variation, and TNFRSF10A-Capivasertib (Complex III) demonstrated the widest conformational sampling and showed highly flexible complexes. (B) SASA analysis revealed the binding free energy results, showing the structural thermodynamics and structural superiority. Complex I (~130 Å2) showed the lowest SASA, with higher integrity than Complex II and III. (C) MM-PBSA was done to check the binding free energies of the individual complexes; Complex I showed a binding free energy of −53.92 kcal/mol, Complex II gave a binding free energy of −48 kcal/mol, and Complex III showed an energy of −51.34 kcal/mol. This showed that the stability of complexes during the 300 ns run was Complex I > Complex II > Complex III.

The PCA analysis evaluated the essential collective motions of protein-ligand complexes during the MD simulations. The first two principal components (PC1 and PC2) captured the dominant conformational fluctuations contributing to the system dynamics. TNFRSF10A-Temsirolimus displayed the smallest spread in PC1 and PC2 trajectories (PC1: mean 1.5 ± 0.25; PC2: 0.8 ± 1.15) indicating limited conformational transitions during the simulation, TNFRSF10A-ergotamine showed moderate variance (PC1: mean 2.0 ± 0.3; PC2: 1.0 ± 0.2) implying moderate structural stability, and TNFRSF10A-capivasertib exhibited the widest conformational sampling (PC1: mean 2.5 ± 0.35; PC2: 1.2 ± 0.25) that was indicative of a highly flexible complex. Coming to the solvent-accessible surface area (SASA) analysis, the TNFRSF10A-Temsirolimus complex demonstrated the lowest average SASA (~130 Å^2^) across 300 ns simulations, with minimal fluctuations. TNFRSF10A-ergotamine complex maintained a moderate SASA (~145 Å^2^) with periodic volatility. TNFRSF10A-capivasertib complex exhibited the highest average SASA (~160 Å^2^) with the greatest variability, indicating that this complex is less conformationally stable. These SASA trends corroborate the binding free energy results, reinforcing Temsirolimus thermodynamic and structural superiority as a potential ligand for TNFRSF10A. The thermodynamic stability and the binding affinities of candidate compounds targeting TNFRSF10A were performed using MM/PBSA analysis on a timescale of 300ns MD simulation trajectories. Computed binding free energies (delta G_bind) quantitatively measure the interaction strength between the TNFRSF10A and each compound. In the case of TNFRSF10A, the average amount of binding free energy was −35.92 kcal/mol. Ergotamine showed moderate fluctuations during the simulation, with transient dips reaching up to −48 kcal/mol, indicating favorable but flexible binding. Its early binding stability (0–50ns) was consistent, gradually stabilizing. The TNFRSF10A-temsirolimus complex showed the strongest binding affinities, with average delta G_bind consistently below −40 kcal/mol, peaking at −51.34 kcal/mol. The minimal fluctuation and sustained negative energy profile suggest tight and stable binding, likely mediated by strong van der Waals and electrostatic interactions. Capivasertib displayed the weakest interactions, with an average delta G_bind near −25 to −30 kcal/mol. Here, the greater fluctuations and a less negative energy profile imply transient or unstable binding, making it a less promising candidate in comparison. These results position temsirolimus and ergotamine as the most energetically favorable binders to TNFRSF10A, aligning with its known mTOR inhibitory role, which may synergize with TNFRSF10A-mediated apoptosis pathways in PDAC. These results and plots can be seen in Fig. 12(A–C).

A comparative tabular representation of all the parameters can be seen in Table 5.

## 4. Discussion

This multi-faceted study proves that TNFRSF10A/TRAIL1 or death receptor-4 is an important key culprit in PDAC. It gives us an idea of the pharmacological targeting rather than the flagrant resistance to the current therapeutic regime. This concurrence is obtained from the molecular, spatial, and computational arena of inquiry. Firstly, the transcriptomic analyses, including both spatial and bulk, suggested TNFRSF10A to be highly elevated in PDAC cells compared to normal tissue. The integration of the single-cell angle showed us that TNFRSF10A is principally expressed in malignant epithelial cells, along with a set of stromal cells (supposedly activated fibroblasts) on the stromal-tumor interface. This sort of relevant distribution gives an important advantage for therapeutic targeting. Studies have shown that CAFs are an important source of various immunosuppressive signals (such as CXCL12) that expel the cytotoxic T-cells from the lesions generated in PDAC [29]. We hypothesized that triggering the death receptor signaling in the cancer cells could omit the need for p53 or other defective intrinsic apoptotic pathways, thereby crooking an important path that normal cells overcome (TRAIL-induced apoptosis is relatively selective for transformed cells) [30]. This dual compartmental channeling of the stromal and tumor components shows a concept of resolving tumor compartment modalities. TNFRSF10A is traditionally known as pro-apoptotic, meaning most studies in other cancers focused on an agonist approach [31,32]. However, recent studies showed that under certain conditions, TNFRSF10A signaling can activate pro-survival and pro-inflammatory pathways (e.g., NF-kappa B activation, metastasis, and invasion) rather than inducing apoptosis [33]. In the case of PDAC specifically, high expression of TNFRSF10A might enhance tumor survival, migration, immune suppression, or resistance to chemotherapy [34]. As TNFRSF10A/TRAIL-1 activation conventionally induces apoptosis, recent studies suggest complex, context-dependent roles. In PDAC, higher TNFRSF10A expressions in the CAFs and tumor cells are associated with apoptotic resistance and enhanced tumor aggressiveness, immunosuppression, and metastatic capability [35]. Therefore, TNFRSF10A signaling can paradoxically stimulate the pro-survival NF-ΚB pathway and inflammation-driven tumor progressions [36]. These findings suggest that antagonizing TNFRSF10A is a compelling therapeutic strategy to selectively halt pro-tumorigenic signaling, thereby reversing apoptosis resistance and modulating the hostile TME [37].

The most novel aspect of this work is the AI-driven drug discovery pipeline that helped us to predict three repurposed drug candidates, i.e., temsirolimus, Ergotamine, and capivasertib, as TRAILR1 modulators. We performed the integrated molecular docking and MD simulations and found that temsirolimus showed higher stability and stronger binding to the modeled TNFRSF10A receptor. TNFRSF10A-temsirolimus maintained a low RMSD after an initial settling period. In contrast, the PCA analysis of the receptor’s motion showed a limited conformational drift (a tight cluster in phase space), effectively showing that temsirolimus effectively “locks” in a defined conformation. On the other hand, the SASA of the TNFRSF10A potential ligand binding sites was lower when it showed binding to temsirolimus (nearly 130 Å2 compared to 145 Å2 when bound to Ergotamine), which is consistent with temsirolimus showing deep engagement of the TNFRSF10A pockets and blocking up the solvent. When talking about binding thermodynamics, temsirolimus showed the most favorable free energy of MM-PBSA (−45 kcal/mol on average, 10 kcal/mol comparatively stronger than Ergotamine. More H-bonds were seen to be present when bound to TNFRSF10A, nearly 6–7 simultaneously during the trajectory, showing more polar contacts. These properties reinforce temsirolimus as a potential lead compound targeting TNFRSF10A/TRAILR1. Therefore, temsirolimus shows dual effects-binds to the death receptor externally while dominating the downstream apoptosis machine internally, showing a synergistic approach highly required in aggressive, resistant cancer.

On the other hand, Ergotamine [38] Exhibited moderately strong binding interactions, and its complex with TNFRSF10A was stable, along with a steady RMSD and RMSF. A higher conformational flexibility was observed than with temsirolimus (showing a higher spread of PC1/PC2 motions). The MM-PBSA binding energy for Ergotamine was ~−35 kcal/mol, suggesting a more stable interaction. Researchers have shown that Ergotamine, a small and more rigid polycyclic molecule, could act as an allosteric modulator of TNFRSF10A. This means that it can stabilize the receptor conformations permissive to downstream signaling or perhaps cause the formation of receptor clusters necessary for death-inducing signaling complex assembly (DISC). Capivasertib (AKT inhibitor) also showed a compatible binding affinity of −9 kcal/mol and a good MD stability index. Capivasertib showed intermediate binding energetics and stability (MM/PBSA around ~ −29 kcal/mol). The overall RMSD, RMSF, and Rg showed fluctuation in the beginning but somewhat stabilized as it reached the end of 300 ns Many studies claim that targeting the TNFRSF10A using an agonist is a more promising approach; however, we wanted to use an antagonist approach of selective targeting. The goal was to induce cell apoptosis simultaneously, turn off the stromal defense system, and stimulate immune attack-a combination that facilitated the therapeutic resistance of PDAC. Integrating spatial multi-omics and AI-leveraged drug discovery in our study has enlightened a promising therapeutic approach in PDAC. We identified TNFRSF10A/TRAILR1 as a promising target at the intersection of cancer cell apoptosis and the immunosuppressive microenvironment. Temsirolimus, a known mTOR inhibitor [39], showed a stable receptor binding, suggestive of the dual mechanistic potential by blocking the TNFRSF10A pro-survival signaling while concurrently inhibiting the mTOR-mediated pathways. On the other hand, ergotamine [38] Stable binding suggests a novel role in modulating the tumor stromal signaling axis.

Along with that, we also uncovered readily available compounds that can target this TNFRSF10A. This approach exemplifies how combining cutting-edge genomics, systems biology, and artificial intelligence could give us a reasonable insight into most treatment-resistant cancers. Multiprolonged strategies are crucial as a single treatment approach fails to induce a better response. Our study, therefore, not only provides better therapeutic avenues but also serves as an optimal paradigm for next-generation drug discovery in the field of oncology, where the combination of omics data and AI could unlock new ways for long-standing challenges in the field of cancer.

## 5. Conclusion

Our research revealed that targeting TNFRSF10A/TRAILR1 in the PDAC microenvironment could provide a novel therapeutic strategy, as TNFRSF10A activation influences the pro-survival signaling. Our antagonist-based approach fundamentally differs from traditional TNFRSF10A/TRAILR1 agonist studies. Moreover, this study presents an AI-driven drug discovery framework integrating deep learning, QSAR-based clustering, docking, MD simulation analysis, and predicting temsirolimus, ergotamine, and capivasertib as promising candidates for PDAC therapy. Our study also highlighted the ability of SELFormer-based deep learning to precisely identify potential therapeutic avenues in PDAC. Our work could be used by researchers in the field of cancer biology and clinicians targeting TNFRSF10A/TRAILR1 in pancreatic cancer. We envisage that these findings can inform clinical investigations. The identified targeting compounds could be prioritized for preclinical testing and early-phase clinical trials in PDAC, potentially guiding biomarker-driven patient stratification. Future studies will dissect additional layers of TNFSF10A regulation (such as post-translational modifications or interacting partners) and experimentally validate the predicted drug candidates in relevant PDAC models. We also acknowledge limitations in our study. Since this approach relies on publicly available omics datasets and extensive artificial intelligence and machine learning predictions, it further requires more validation. Despite these limitations, our integrated pipeline provides a foundation for further research to translate these insights into effective PDAC therapies.

## Supplementary Material

MMC1

Appendix A. Supplementary data

Supplementary data to this article can be found online at https://doi.org/10.1016/j.compbiomed.2025.110432.

## Figures and Tables

**Table 1:** The degree and the betweenness of interacting differential genes with associated miRNAs/proteins are shown in tabular form.

Id	Label	Degree	Betweenness	Interacting miRNAs/Proteins

10499	NCOA2	61	18916.67	miR-4524a-3p; H6PD, SH2D3C
1108	H6PD	51	16607.79	miR-1, miR-133b, miR-133a, miR-2016; ALDOPB, G6PD
4684	NCAM1	48	13230.15	miR-141–3p; FGFR1, GDNF
3714	**TNFRSF10A**	42	11842.46	miR-182, miR-155, miR-24, miR-124; EPHA2, HPGD, SP1
10044	SH2D3C	41	13336.6	miR-545, miR-546, miR-181a-5p
5979	RET	39	9664.22	miR-4297; COL4A4, EHMT1
1294	COL7A1	35	9873.82	miR-1262, miR-4524a-3p; COL4A4, DHRS3
9249	DHRS3	33	8579.4	miR-210; RDH10
1286	COL4A4	26	6435.08	miR-29a, COL4A5
1969	EPHA2	25	6343.78	miR-4701–3p, miR-3297; TNFRSF10A, PLR2E
6002	RGS12	22	6248.02	TEAD1, YAP
79813	EHMT1	18	4771.27	EHMT2,
9563	CHD4	13	3174.62	miR-767–3p
30845	EHD3	11	2515.82	RAB8A, RAB22A
8797	JAG2	10	1512.91	miR-34a, NOTCH 1
6667	SP1	7	8852.7	miR-24; TNFRSF10A. JUN
23086	EXPH5	7	1969.48	RAB27A
3725	JUN	5	3508.32	miR1–55; SP1, TNFRSF10A

**Table 2:** Comparative analysis of the individual models based on different scores.

ML models	R^2^ Score	Root mean square error (RMSE)	Mean absolute error (MAE)

Random forest (RF)	0.65	1.35	0.82
Support vector regression (SVR)	0.28	2	1.25
LIghtGBM	0.58	1.55	0.9
Gradient Boosting	0.6	1.5	0.9
XGBoost	0.65	1.35	0.82

**Table 3:** Tabular representation of all the binding interactions and the key residue involved during MD docking analysis.

Compound	Binding Energy (kcal/mol)	Number of interactions	Key residues involved	Interaction type

Temsirolimus	−10.1	6	Glu50, Arg72, Trp100, Thr44, Cys64, Asn61	Hydrogen bonds, Hydrophobic (Pi-Pi, Pi-Alkyl), Electrostatic

Ergotamine	−9.6	7	Glu50, Arg72, Trp100, Ala45, Ala77, Lys47, Tyr30	Hydrogen bonds, Hydrophobic (Pi-Pi, Pi-Alkyl), Electrostatic

Capivasertib	−9	7	Glu50, Arg72, Trp100, Ala77, Asp74, Asn73	Hydrogen bonds, Hydrophobic (Pi-Pi, Pi-Alkyl), Electrostatic

**Table 4:** Tabular representation of the interaction energies in Coulombs (SR) and Lennard Jones (LJ-SR).

Ergotamine				

Energy	Average	Err. Estimate	RMSD	Total Drift

Coulomb (SR)	−1.07E+06	10	1628.72	−77.951
LJ (SR)	102124	11	988.2	−36.0373

Temsirolimus				

Energy	Average	Err. Estimate	RMSD	Total Drift

Coulomb (SR)	−1.02E+05	8.2	1648.72	−37.87
LJ (SR)	101997	13	989.044	−36.0373

**Table 5:** Tabular representation of the comparative scores SASA, PCA and MM_PBSA for temsirolimus, ergotamine and capivasertib.

Parameter	Temsirolimus	Ergotamine	Capivasertib
Average. SASA (Å^2^)	~130	~145	~160
PCA Conformational Spread	Low	Moderate	High
Binding affinities (kcal/mol)	Most Negative	Moderate	Least Negative

## Data Availability

Data will be made available on request.
