# Supplementary material for: Integrating AI/ML and multi-omics approaches to investigate the role of TNFRSF10A/TRAILR1 and its potential targets in pancreatic cancer
Source: Comput Biol Med. Author manuscript; Available in PMC 2025 Jul 1. (PMC12204372; doi:10.1016/j.compbiomed.2025.110432)
Supplement: MMC1 [file NIHMS2086068-supplement-MMC1.docx]

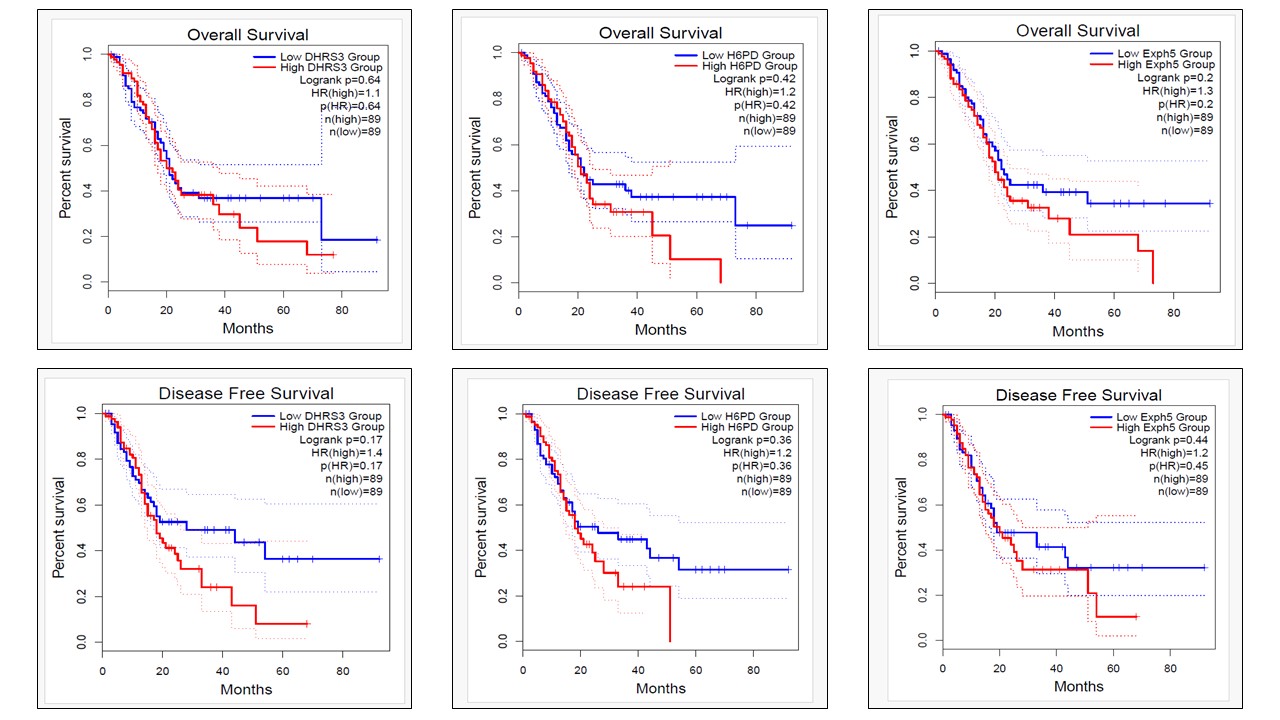

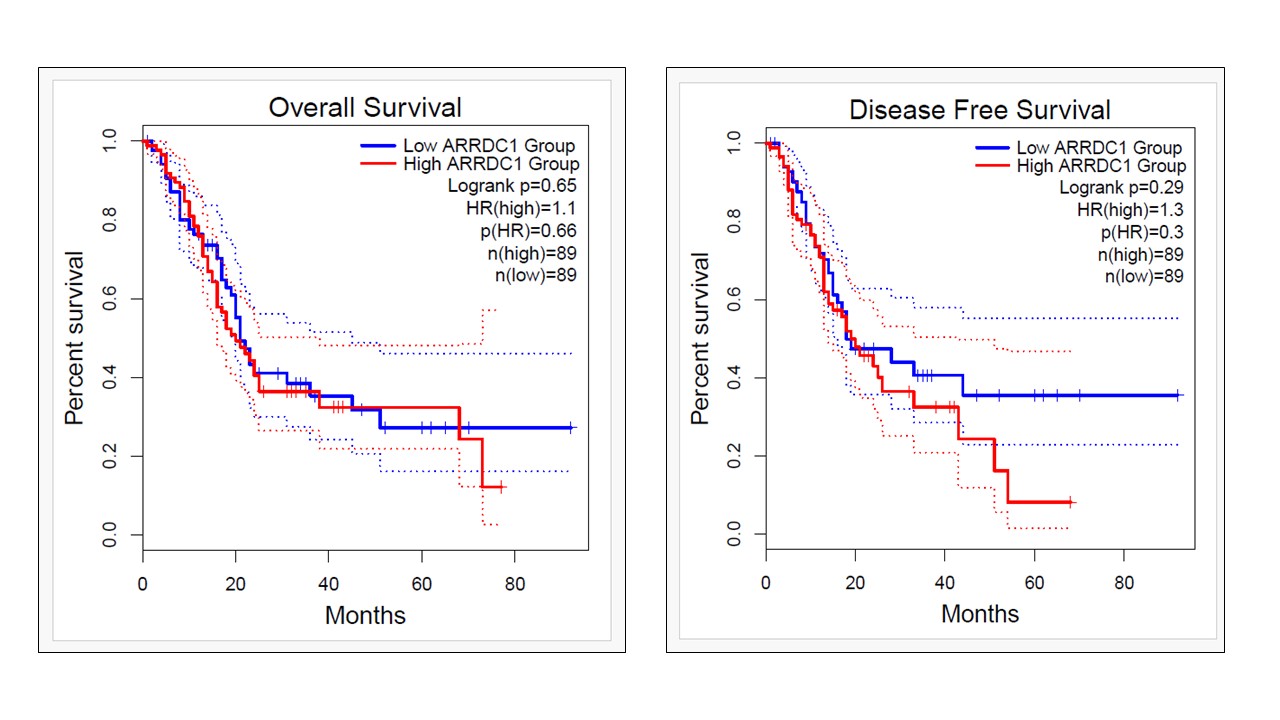
**Supplementary Figure 1:** Representation of the overall survival and the disease-free survival of DHRS3, H6PD, EXPH5 and ARRD1

**
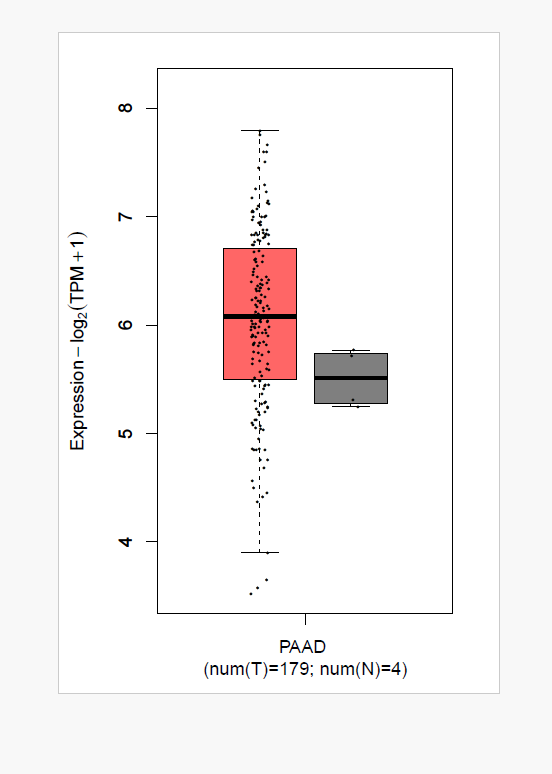

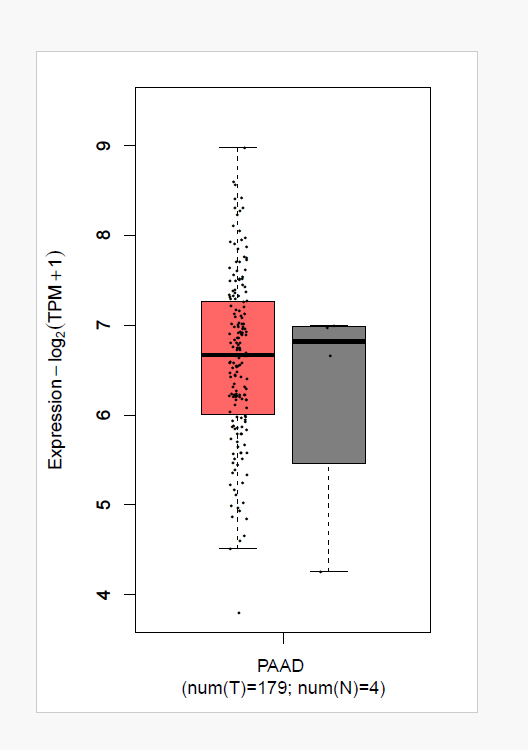
**

**
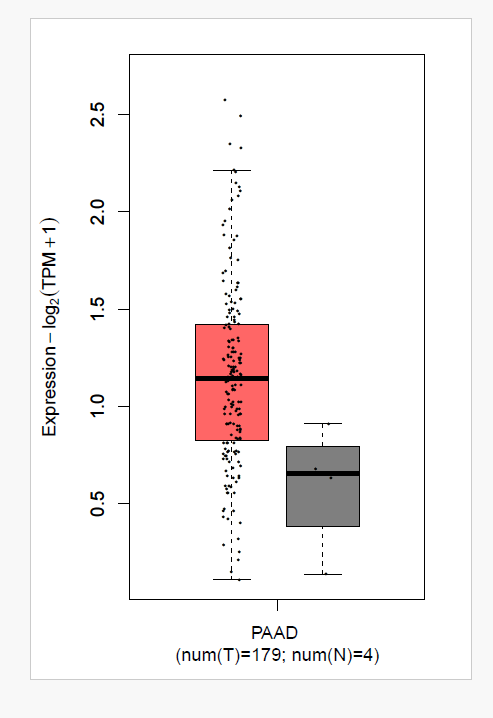

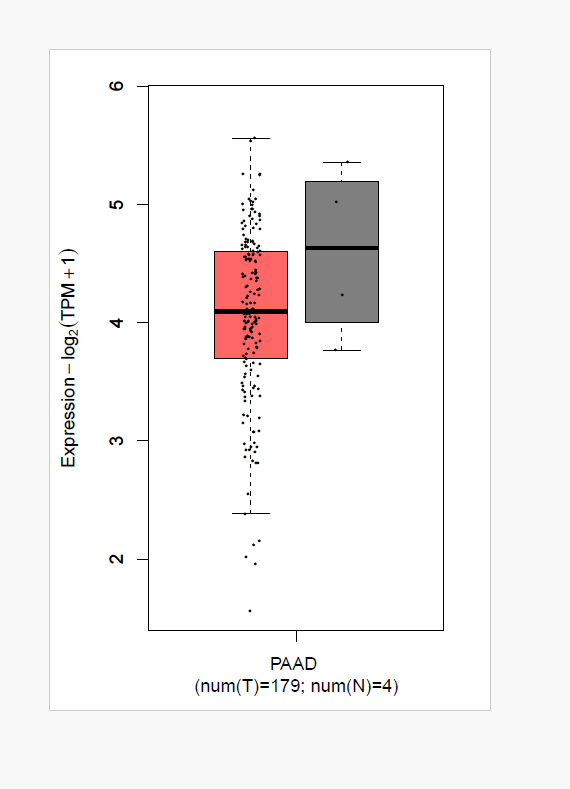
**

**Supplementary Figure 2:** Representation of the expression analysis performed using GEPAI for ARRDC1, DHRS3, EXPH5, and H6PD


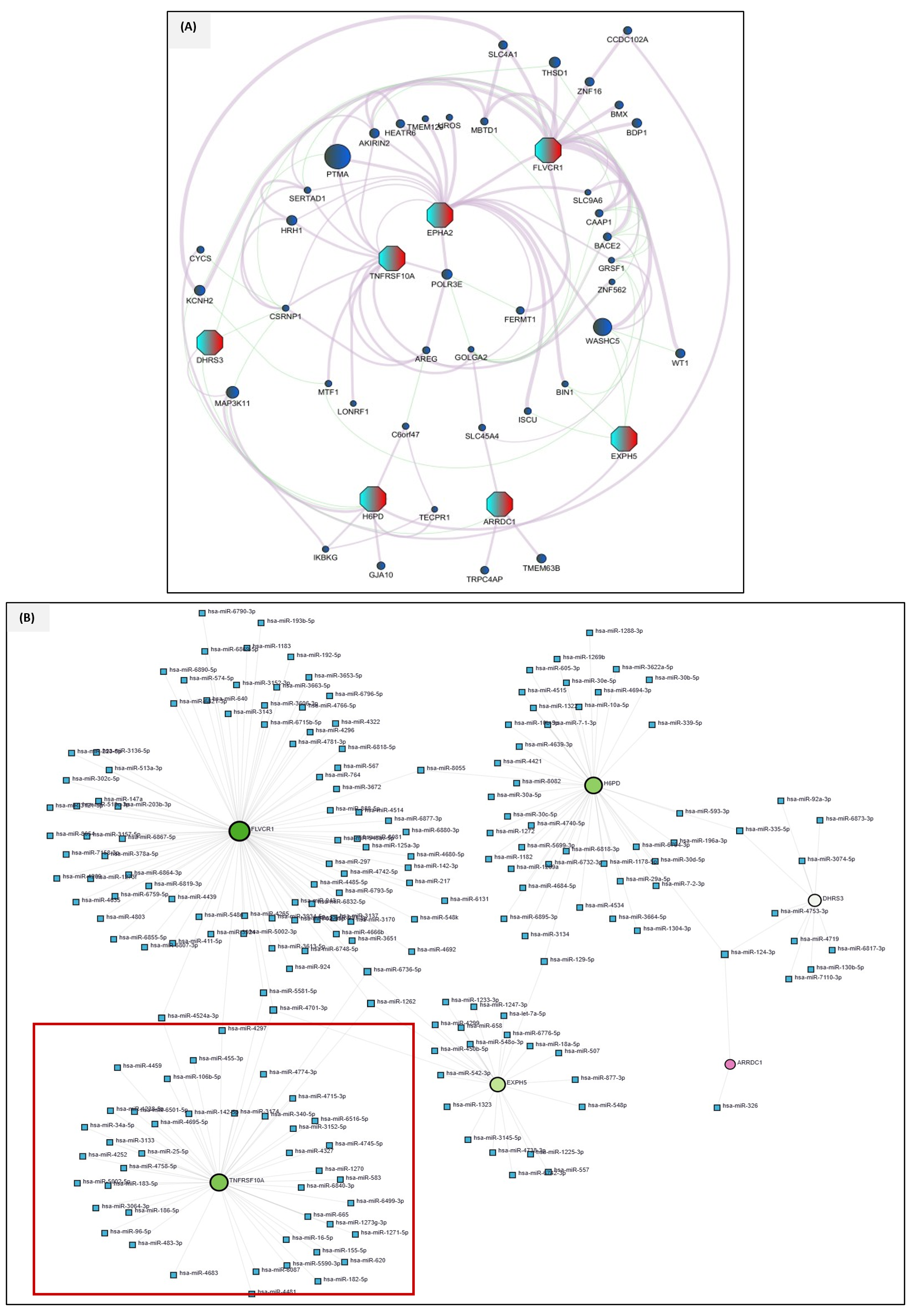


**Supplementary Figure 3:** Representation of the ceRNA regulatory network of the interacting miRNAs with top differential markers and the protein interaction network generated using Cytoscape and.(A) Representation of the Ramachandran plot for Chain D (PDB: 5CIR) after performing the homology modeling using MODELLER for the missing residues. (B) It shows the crystal structure of Chain D of TNFRSF10A/TRAILR1, which was cleaned of any unnecessary ligands or heteroatoms.


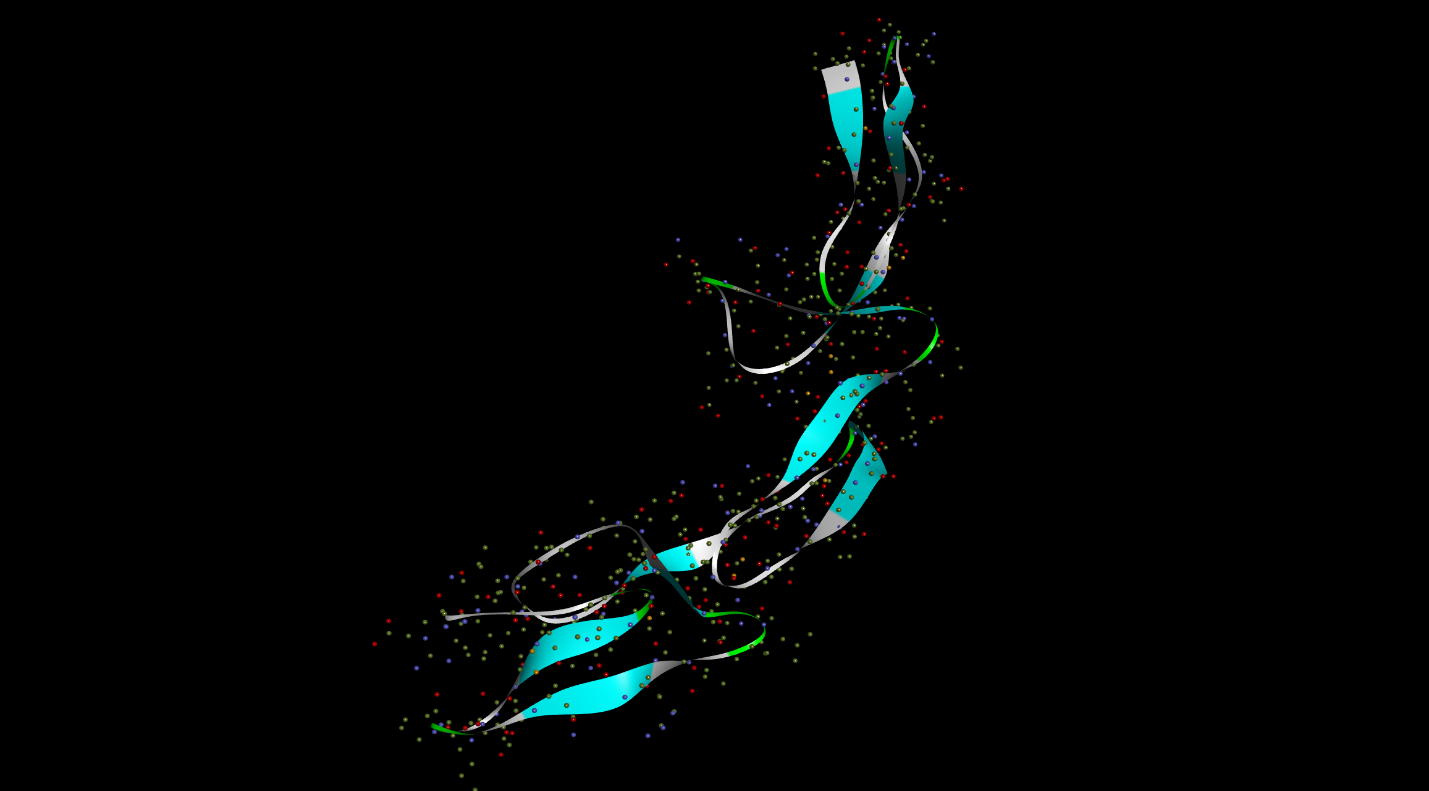

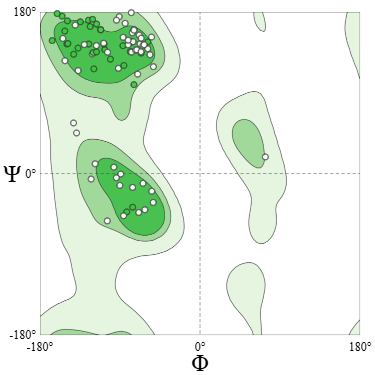


**Supplementary Table 1:** Tabular representation of the enrichment analysis performed at various levels, GO_Biological process

| Gene_set | Term | P-value | Combined Score | Genes |
| --- | --- | --- | --- | --- |
| GO_Biological_Process_2018 | positive regulation of response to DNA damage stimulus (GO:2001022) | 5.52E-08 | 21744.68822 | MYC; BRCA1; EGFR; TNFRSF10a |
| GO_Biological_Process_2018 | response to ionizing radiation (GO:0010212) | 2.19E-07 | 12385.19749 | MYC; BRCA1; TP53 |
| GO_Biological_Process_2018 | positive regulation of DNA metabolic process (GO:0051054) | 2.65E-07 | 11453.96423 | MYC; BRCA1; EGFR |
| GO_Biological_Process_2018 | positive regulation of nucleic acid-templated transcription (GO:1903508) | 3.92E-07 | 1150486.005 | MYC; BRCA1; TP53;EGFR |
| GO_Biological_Process_2018 | response to UV (GO:0009411) | 4.55E-07 | 9177.710371 | MYC;TP53;EGFR |
| GO_Biological_Process_2018 | regulation of cell proliferation (GO:0042127) | 1.86E-06 | 1016562.321 | MYC;BRCA1;TP53;EGFR |
| GO_Biological_Process_2018 | positive regulation of gene expression (GO:0010628) | 2.19E-06 | 1002274.826 | MYC;BRCA1;TP53;EGFR |
| GO_Biological_Process_2018 | DNA damage response, signal transduction by p53 class mediator resulting in transcription of p21 class mediator (GO:0006978) | 2.34E-06 | 23557.08871 | BRCA1;TP53 |
| GO_Biological_Process_2018 | DNA damage response, signal transduction resulting in transcription (GO:0042772) | 2.73E-06 | 21336.31313 | BRCA1;TP53 |
| GO_Biological_Process_2018 | regulation of apoptotic process (GO:0042981) | 2.74E-06 | 982912.6632 | MYC;BRCA1;TP53;EGFR |
| GO_Biological_Process_2018 | positive regulation of transcription from RNA polymerase II promoter (GO:0045944) | 3.21E-06 | 969036.7448 | MYC;BRCA1;TP53;EGFR |
| GO_Biological_Process_2018 | negative regulation of fibroblast proliferation (GO:0048147) | 4.59E-06 | 15350.53372 | MYC;TP53 |
| GO_Biological_Process_2018 | protein deubiquitination (GO:0016579) | 8.31E-06 | 2727.692803 | MYC;BRCA1;TP53 |
| GO_Biological_Process_2018 | protein modification by small protein removal (GO:0070646) | 8.70E-06 | 2674.21826 | MYC;BRCA1;TP53 |
| GO_Biological_Process_2018 | response to gamma radiation (GO:0010332) | 9.73E-06 | 9603.084843 | MYC;TP53 |
| GO_Biological_Process_2018 | positive regulation of transcription, DNA-templated (GO:0045893) | 9.78E-06 | 871103.6033 | MYC;BRCA1;TP53;EGFR |
| GO_Biological_Process_2018 | positive regulation of macromolecule metabolic process (GO:0010604) | 1.03E-05 | 2489.015124 | MYC;BRCA1;TP53 |
| GO_Biological_Process_2018 | positive regulation of histone modification (GO:0031058) | 1.22E-05 | 8370.359426 | BRCA1;TP53 |
| GO_Biological_Process_2018 | cellular response to drug (GO:0035690) | 1.30E-05 | 8021.859107 | MYC;TP53 |
| GO_Biological_Process_2018 | cellular response to DNA damage stimulus (GO:0006974) | 1.74E-05 | 1983.414171 | TRAILR1 |
| GO_Biological_Process_2018 | regulation of DNA repair (GO:0006282) | 1.99E-05 | 6172.579702 | BRCA1;EGFR |
| GO_Biological_Process_2018 | positive regulation of DNA repair (GO:0045739) | 2.10E-05 | 5970.877941 | BRCA1;EGFR |
| GO_Biological_Process_2018 | regulation of stress-activated MAPK cascade (GO:0032872) | 2.45E-05 | 5432.07524 | MYC;EGFR |
| GO_Biological_Process_2018 | regulation of telomerase activity (GO:0051972) | 2.58E-05 | 5271.69962 | MYC;TP53 |
| GO_Biological_Process_2018 | regulation of transcription from RNA polymerase II promoter (GO:0006357) | 2.97E-05 | 772292.1523 | MYC;BRCA1;TP53;EGFR |
| GO_Biological_Process_2018 | regulation of fibroblast proliferation (GO:0048145) | 3.10E-05 | 4708.150009 | MYC;TP53 |
| GO_Biological_Process_2018 | negative regulation of programmed cell death (GO:0043069) | 3.32E-05 | 1496.598893 | MYC;TP53;EGFR |
| GO_Biological_Process_2018 | intrinsic apoptotic signaling pathway in response to DNA damage (GO:0008630) | 3.37E-05 | 4465.736727 | BRCA1;TP53 |
| GO_Biological_Process_2018 | regulation of cell cycle arrest (GO:0071156) | 3.37E-05 | 4465.736727 | BRCA1;TP53 |
| GO_Biological_Process_2018 | regulation of reactive oxygen species metabolic process (GO:2000377) | 3.52E-05 | 4352.842291 | BRCA1;TP53 |
| GO_Biological_Process_2018 | regulation of intracellular signal transduction (GO:1902531) | 3.67E-05 | 1431.412564 | BRCA1;TP53;EGFR |
| GO_Biological_Process_2018 | regulation of transcription, DNA-templated (GO:0006355) | 4.06E-05 | 744281.6887 | MYC;BRCA1;TP53;EGFR |
| GO_Biological_Process_2018 | positive regulation of response to stimulus (GO:0048584) | 4.28E-05 | 3858.169789 | MYC;EGFR |
| GO_Biological_Process_2018 | cellular response to cytokine stimulus (GO:0071345) | 4.63E-05 | 1291.690677 | MYC;BRCA1;TP53 |
| GO_Biological_Process_2018 | negative regulation of apoptotic process (GO:0043066) | 5.57E-05 | 1189.794142 | MYC;TP53;EGFR; TNFRSF10A |

**Supplementary Table 2:** Tabular representation of the model comparison scores after the

run

| Model | R² Score | RMSE (Lower is Better) | MAE (Lower is Better) |
| --- | --- | --- | --- |
| Random Forest | 0.45 | 33500 | 5,600 |
| XGBoost | 0.53 | 31000 | 5,296 |
| Support Vector Regression | 0.06 | 45000 | 6,215 |

**Supplementary Table 3:** Tabular representation of the binding affinities and their properties of top 500 compounds

| Binding Affinity (kcal/mol) | Compound names | canonical_smiles | LogP | Hydrogen_Bond_Acceptors | Hydrogen_Bond_Donors | Polar_Surface_Area | Molecular_Weight | Full_Molecular_Weight |
| --- | --- | --- | --- | --- | --- | --- | --- | --- |
| -9.6 | Ergotamine | CN1C[C@H](C(=O)N[C@]2(C)O[C@@]3(O)[C@@H]4CCCN4C(=O)[C@H](Cc4ccccc4)N3C2=O)C=C2c3cccc4[nH]cc(c34)C[C@H]21 | 1.99 | 6 | 3 | 118.21 | 581.67 | 581.67 |
| -9 | Capivasertib | NC1(C(=O)N[C@@H](CCO)c2ccc(Cl)cc2)CCN(c2nc[nH]c3nccc2-3)CC1 | 2.1 | 6 | 4 | 120.16 | 428.92 | 428.92 |
| -10.1 | Temsirolimus | CO[C@H]1C[C@@H]2CC[C@@H](C)[C@@](O)(O2)C(=O)C(=O)N2CCCC[C@H]2C(=O)O[C@H]([C@H](C)C[C@@H]2CC[C@@H](OC(=O)C(C)(CO)CO)[C@H](OC)C2)CC(=O)[C@H](C)/C=C(\C)[C@@H](O)[C@@H](OC)C(=O)[C@H](C)C[C@H](C)/C=C/C=C/C=C/1C |  |  |  |  | 1030.3 | 1030.3 |
| -8 | Digitoxin | COc1cc2nc(N3CCN(C(=O)c4ccco4)CC3)nc(N)c2cc1OC | 1.78 | 8 | 1 | 106.95 | 383.41 | 383.41 |
| -7.8 | Quinupristin | CN1CCC[C@H]1c1cccnc1 | 1.85 | 2 | 0 | 16.13 | 162.24 | 162.24 |
| -7.8 | Eribulin | CC1COc2c(N3CCN(C)CC3)c(F)cc3c(=O)c(C(=O)O)cn1c23 | 1.54 | 6 | 1 | 75.01 | 361.37 | 361.37 |
| -7.8 | Itraconazole | CCn1cc(C(=O)O)c(=O)c2ccc(C)nc21 | 1.42 | 4 | 1 | 72.19 | 232.24 | 232.24 |
| -7.7 | Rifapentine | COc1ccc2c(c1)c(CC(=O)O)c(C)n2C(=O)c1ccc(Cl)cc1 | 3.93 | 4 | 1 | 68.53 | 357.79 | 357.79 |
| -7.7 | Vapreotide | CC1(C)[C@H](C(=O)O)N2C(=O)C[C@H]2S1(=O)=O | -0.79 | 4 | 1 | 91.75 | 233.24 | 233.24 |
| -7.7 | Acetyldigitoxin | C[C@]1(Cn2ccnn2)[C@H](C(=O)O)N2C(=O)C[C@H]2S1(=O)=O | -1.52 | 7 | 1 | 122.46 | 300.3 | 300.3 |
| -7.6 | Everolimus | O=C(O)c1cn(C2CC2)c2cc(N3CCNCC3)c(F)cc2c1=O | 1.58 | 5 | 2 | 74.57 | 331.35 | 331.35 |
| -7.6 | Formoterol | CCn1cc(C(=O)O)c(=O)c2cc(F)c(N3CCNCC3)cc21 | 1.27 | 5 | 2 | 74.57 | 319.34 | 319.34 |
| -7.5 | Plicamycin | CC(N)Cc1ccccc1 | 1.58 | 1 | 1 | 26.02 | 135.21 | 135.21 |
| -7.5 | Rifaximin | O=[N+]([O-])O[C@H]1CO[C@H]2[C@@H]1OC[C@H]2O[N+](=O)[O-] | -1.06 | 8 | 0 | 123.2 | 236.14 | 236.14 |
| -7.5 | Saquinavir | CN1CCN2c3ccccc3Cc3ccccc3C2C1 | 3.08 | 2 | 0 | 6.48 | 264.37 | 264.37 |
| -7.5 | Dabrafenib | CN(C)CCCN1c2ccccc2CCc2ccccc21 | 3.88 | 2 | 0 | 6.48 | 280.42 | 280.42 |
| -7.5 | Dutasteride | CC1Cc2ccccc2N1NC(=O)c1ccc(Cl)c(S(N)(=O)=O)c1 | 2.08 | 4 | 2 | 92.5 | 365.84 | 365.84 |
| -7.5 | Etoposide | CCOC(=O)c1ncn2c1CN(C)C(=O)c1cc(F)ccc1-2 | 1.77 | 5 | 0 | 64.43 | 303.29 | 303.29 |
| -7.5 | Fluorescein | Cc1c(C)c2c(c(C)c1O)CCC(C)(COc1ccc(CC3SC(=O)NC3=O)cc1)O2 | 4.37 | 6 | 2 | 84.86 | 441.55 | 441.55 |
| -7.5 | Fosaprepitant | CC(O)(CS(=O)(=O)c1ccc(F)cc1)C(=O)Nc1ccc(C#N)c(C(F)(F)F)c1 | 2.88 | 5 | 2 | 107.26 | 430.38 | 430.38 |
| -7.5 | Linaclotide | CN1C(=O)CN=C(c2ccccc2)c2cc(Cl)ccc21 | 3.15 | 2 | 0 | 32.67 | 284.75 | 284.75 |
| -7.4 | Rifampin | COCCc1ccc(OCC(O)CNC(C)C)cc1 | 1.61 | 4 | 2 | 50.72 | 267.37 | 267.37 |
| -7.4 | Tubocurarine | CC/C(=C(/CC)c1ccc(O)cc1)c1ccc(O)cc1 | 4.83 | 2 | 2 | 40.46 | 268.36 | 268.36 |
| -7.4 | Valrubicin | CNC1(C)C2CCC(C2)C1(C)C | 2.42 | 1 | 1 | 12.03 | 167.3 | 167.3 |
| -7.4 | Vancomycin | C[N+](C)(C)CCOC(N)=O.[Cl-] | -0.21 | 2 | 1 | 52.32 | 147.2 | 182.65 |
| -7.4 | Conivaptan | O=C1NC(=O)C(c2ccccc2)(c2ccccc2)N1 | 1.77 | 2 | 2 | 58.2 | 252.27 | 252.27 |
| -7.4 | Demeclocycline | CO[C@H]1C[C@@H]2CC[C@@H](C)[C@@](O)(O2)C(=O)C(=O)N2CCCC[C@H]2C(=O)O[C@H]([C@H](C)C[C@@H]2CC[C@@H](O)[C@H](OC)C2)CC(=O)[C@H](C)/C=C(\C)[C@@H](O)[C@@H](OC)C(=O)[C@H](C)C[C@H](C)/C=C/C=C/C=C/1C | 6.18 | 13 | 3 | 195.43 | 914.19 | 914.19 |
| -7.4 | Eltrombopag | C=CC[C@@H]1/C=C(\C)C[C@H](C)C[C@H](OC)[C@H]2O[C@@](O)(C(=O)C(=O)N3CCCC[C@H]3C(=O)O[C@H](/C(C)=C/[C@@H]3CC[C@@H](O)[C@H](OC)C3)[C@H](C)[C@@H](O)CC1=O)[C@H](C)C[C@@H]2OC | 4.64 | 12 | 3 | 178.36 | 804.03 | 804.03 |
| -7.4 | Amcinonide | CN(C)CCCN1c2ccccc2CCc2ccc(Cl)cc21 | 4.53 | 2 | 0 | 6.48 | 314.86 | 314.86 |
| -7.3 | Pimecrolimus | COc1c2occc2cc2ccc(=O)oc12 | 2.55 | 4 | 0 | 52.58 | 216.19 | 216.19 |
| -7.3 | Rifabutin | NS(=O)(=O)c1cc(Cl)c(Cl)c(S(N)(=O)=O)c1 | 0.29 | 4 | 2 | 120.32 | 305.16 | 305.16 |
| -7.3 | Sirolimus | CCOc1ccc2nc(S(N)(=O)=O)sc2c1 | 1.34 | 5 | 1 | 82.28 | 258.32 | 258.32 |
| -7.3 | Telmisartan | COc1cccc2c1C(=O)c1c(O)c3c(c(O)c1C2=O)C[C@@](O)(C(=O)CO)C[C@@H]3O[C@H]1C[C@H](N)[C@@H](O)[C@H](C)O1 | 0 | 12 | 6 | 206.07 | 543.53 | 543.53 |
| -7.3 | Teniposide | CC(=O)/N=c1/sc(S(N)(=O)=O)nn1C | -1.42 | 6 | 1 | 107.41 | 236.28 | 236.28 |
| -7.3 | Bromocriptine | CC(=O)Nc1nnc(S(N)(=O)=O)s1 | -0.86 | 6 | 2 | 115.04 | 222.25 | 222.25 |
| -7.3 | Carbetocin | NCc1ccc(S(N)(=O)=O)cc1 | -0.21 | 3 | 2 | 86.18 | 186.24 | 186.24 |
| -7.3 | Caspofungin | Nc1ccc(S(N)(=O)=O)cc1 | -0.08 | 3 | 2 | 86.18 | 172.21 | 172.21 |
| -7.3 | Desmopressin | N=C(N)N/N=C/c1c(Cl)cccc1Cl | 1.81 | 2 | 3 | 74.26 | 231.09 | 231.09 |
| -7.3 | Ertapenem | COc1cc(Cc2cnc(N)nc2N)cc(OC)c1OC | 1.26 | 7 | 2 | 105.51 | 290.32 | 290.32 |
| -7.3 | Lopinavir | COc1ccc(CCN(C)CCCC(C#N)(c2ccc(OC)c(OC)c2)C(C)C)cc1OC | 5.09 | 6 | 0 | 63.95 | 454.61 | 454.61 |
| -7.3 | Anidulafungin | O=C(O)c1cc(/N=N/c2ccc(S(=O)(=O)Nc3ccccn3)cc2)ccc1O | 3.7 | 7 | 3 | 141.31 | 398.4 | 398.4 |
| -7.2 | Ponatinib | O=c1ccc2ccccc2o1 | 1.79 | 2 | 0 | 30.21 | 146.14 | 146.14 |
| -7.2 | Spironolactone | COc1ccc([C@@H]2Sc3ccccc3N(CCN(C)C)C(=O)[C@@H]2OC(C)=O)cc1 | 3.37 | 6 | 0 | 59.08 | 414.53 | 414.53 |
| -7.2 | Sulfasalazine | CC(C)NCC(O)COc1ccc(CC(N)=O)cc1 | 0.45 | 4 | 3 | 84.58 | 266.34 | 266.34 |
| -7.2 | Trametinib | CC(=O)Oc1ccccc1C(=O)O | 1.31 | 3 | 1 | 63.6 | 180.16 | 180.16 |
| -7.2 | Bosentan | CN1CCN(CCCN2c3ccccc3Sc3ccc(C(F)(F)F)cc32)CC1 | 4.95 | 4 | 0 | 9.72 | 407.51 | 407.51 |
| -7.2 | Daunorubicin | CCN1CCCC1CNC(=O)c1cc(S(N)(=O)=O)ccc1OC | 0.56 | 5 | 2 | 101.73 | 341.43 | 341.43 |
| -7.2 | Deserpidine | CC(C)NCC(O)COc1ccc(CCOCC2CC2)cc1 | 2.39 | 4 | 2 | 50.72 | 307.43 | 307.43 |
| -8.1 | morphine | O=C(O)c1ccccc1O | 1.09 | 2 | 2 | 57.53 | 138.12 | 138.12 |
| -8.1 | triamterene | O=C(O)c1cc(/N=N/c2ccc(O)c(C(=O)O)c2)ccc1O | 2.91 | 6 | 4 | 139.78 | 302.24 | 302.24 |
| -7.9 | oxymorphone | CC(C)NCC(O)COc1cccc2ccccc12 | 2.58 | 3 | 2 | 41.49 | 259.35 | 259.35 |
| -7.7 | Santin | CC(=O)Nc1ccc(OCC(O)CNC(C)C)cc1 | 1.38 | 4 | 3 | 70.59 | 266.34 | 266.34 |
| -7.7 | Medroxyprogesterone acetate | CN(CCCl)CCCl | 1.4 | 1 | 0 | 3.24 | 156.06 | 156.06 |
| -7.7 | Rubitecan | CC1(C)S[C@@H]2[C@H](NC(=O)Cc3ccccc3)C(=O)N2[C@H]1C(=O)O | 0.86 | 4 | 2 | 86.71 | 334.4 | 334.4 |
| -7.6 | acacetin | N[C@@H]1[C@H]2CN(c3nc4c(cc3F)c(=O)c(C(=O)O)cn4-c3ccc(F)cc3F)C[C@@H]12 | 1.89 | 6 | 2 | 101.45 | 416.36 | 416.36 |
| -7.6 | (1)Benzopyrano(3,4-b)furo(2,3-h)(1)benzopyran-6(6aH)-one, 1,2,12,12a-tetrahydro-8,9-dimethoxy-2-(1-methylethenyl)-, (2S-(2alpha,6aalpha,12aalpha))- | CN/C(=N\CCSCc1nc[nH]c1C)NC#N | 0.6 | 4 | 3 | 88.89 | 252.35 | 252.35 |
| -7.6 | Pachyrrhizin | COc1c(N2CCNC(C)C2)c(F)cc2c(=O)c(C(=O)O)cn(C3CC3)c12 | 1.98 | 6 | 2 | 83.8 | 375.4 | 375.4 |
| -7.6 | (1R,13R)-5,7,11,14,18-pentaoxahexacyclo[11.11.0.0Â²,Â¹â°.0â´,â¸.0Â¹âµ,Â²Â³.0Â¹â·,Â²Â¹]tetracosa-2(10),3,8,15,17(21),19,22-heptaen-24-one | CC(CCc1ccccc1)NCC(O)c1ccc(O)c(C(N)=O)c1 | 2.14 | 4 | 4 | 95.58 | 328.41 | 328.41 |
| -7.6 | Diosmetin | COc1c(N2C[C@@H]3CCCN[C@@H]3C2)c(F)cc2c(=O)c(C(=O)O)cn(C3CC3)c12 | 2.37 | 6 | 2 | 83.8 | 401.44 | 401.44 |
| -7.5 | Eupatilin | C[C@H]1COc2c(N3CCN(C)CC3)c(F)cc3c(=O)c(C(=O)O)cn1c23 | 1.54 | 6 | 1 | 75.01 | 361.37 | 361.37 |
| -7.5 | Jaceosidin | CO/N=C1\CN(c2nc3c(cc2F)c(=O)c(C(=O)O)cn3C2CC2)CC1CN | 0.97 | 8 | 2 | 123.04 | 389.39 | 389.39 |
| -7.5 | 4-hydroxybenzaldehyde | CCOC(=O)[C@H](CCc1ccccc1)N[C@@H](C)C(=O)N1CC2(C[C@H]1C(=O)O)SCCS2 | 2.39 | 7 | 2 | 95.94 | 466.63 | 466.63 |
| -7.5 | Denthyrsinin | CCCC(CCC)C(=O)[O-].[Na+] | 2.29 | 1 | 1 | 37.3 | 144.21 | 166.2 |
| -7.5 | 83088-28-2 | CC(C)NCC(O)c1ccc(O)c(O)c1 | 1.13 | 4 | 4 | 72.72 | 211.26 | 211.26 |
| -7.4 | 72966-94-0 | NS(=O)(=O)c1cc2c(cc1Cl)NCNS2(=O)=O | -0.35 | 5 | 3 | 118.36 | 297.75 | 297.75 |
| -7.4 | batatasin III | NS(=O)(=O)c1cc(C(=O)O)c(NCc2ccco2)cc1Cl | 1.89 | 5 | 3 | 122.63 | 330.75 | 330.75 |
| -7.4 | Retusin | CCc1nc(N)nc(N)c1-c1ccc(Cl)cc1 | 2.52 | 4 | 2 | 77.82 | 248.72 | 248.72 |
| -7.4 | Pachypodol | COc1ccc2c(C(=S)N(C)CC(=O)O)cccc2c1C(F)(F)F | 3.56 | 3 | 1 | 49.77 | 357.35 | 357.35 |
| -7.4 | THYMOL | Nc1ccc(S(=O)(=O)Nc2nccs2)cc1 | 1.53 | 5 | 2 | 85.08 | 255.32 | 255.32 |
| -7.4 | Brevipolide G | Cc1ccnc(NS(=O)(=O)c2ccc(N)cc2)n1 | 1.17 | 5 | 2 | 97.97 | 264.31 | 264.31 |
| -7.4 | Brevipolide I | Nc1ccc(S(=O)(=O)Nc2ncccn2)cc1 | 0.86 | 5 | 2 | 97.97 | 250.28 | 250.28 |
| -7.4 | Brevipolide H | C=CCC1(C(C)CCC)C(=O)NC(=S)NC1=O | 1.52 | 3 | 2 | 58.2 | 254.35 | 254.35 |
| -7.4 | 24672-84-2 | CCCC(C)C1(CC)C(=O)NC(=S)NC1=O | 1.35 | 3 | 2 | 58.2 | 242.34 | 242.34 |
| -7.3 | 2-hydroxy-1-(5-hydroxy-2,2-dimethylchromen-6-yl)ethanone | CCC1(C2=CCCCC2)C(=O)NC(=O)NC1=O | 1.25 | 3 | 2 | 75.27 | 236.27 | 236.27 |
| -7.3 | Genkwanin | CN1C[C@H](C(=O)N[C@]2(C)O[C@@]3(O)[C@@H]4CCCN4C(=O)[C@H](Cc4ccccc4)N3C2=O)C=C2c3cccc4[nH]cc(c34)C[C@H]21 | 1.99 | 6 | 3 | 118.21 | 581.67 | 581.67 |
| -7.3 | Isorhamnetin | CC1=C(/C=C/C(C)=C/C=C/C(C)=C/C(=O)O)C(C)(C)CCC1 | 5.6 | 1 | 1 | 37.3 | 300.44 | 300.44 |
| -7.3 | kaempferol | C=CCC1(C(C)C#CCC)C(=O)NC(=O)N(C)C1=O | 1.31 | 3 | 1 | 66.48 | 262.31 | 262.31 |
| -7.3 | quercetin | CCC1(c2ccccc2)C(=O)NC(=O)NC1=O | 0.7 | 3 | 2 | 75.27 | 232.24 | 232.24 |
| -7.3 | Myristicin | CNCCC(Oc1ccc(C(F)(F)F)cc1)c1ccccc1 | 4.44 | 2 | 1 | 21.26 | 309.33 | 309.33 |
| -7.3 | (E/Z)-ferulic acid | Cc1cc(NS(=O)(=O)c2ccc(N)cc2)no1 | 1.37 | 5 | 2 | 98.22 | 253.28 | 253.28 |
| -7.3 | 23790-83-2 | CCC1(CC)C(=O)NC(=O)NC1=O | 0.16 | 3 | 2 | 75.27 | 184.19 | 184.19 |
| -7.3 | Corymbiferone C | CN1CCN(C2=Nc3cc(Cl)ccc3Nc3ccccc32)CC1 | 3.72 | 4 | 1 | 30.87 | 326.83 | 326.83 |
| -7.3 | Epicacalone | CNCCC=C1c2ccccc2CCc2ccccc21 | 3.83 | 1 | 1 | 12.03 | 263.38 | 263.38 |
| -7.2 | (+)-Epicatechin | Cc1cc(C)nc(NS(=O)(=O)c2ccc(N)cc2)n1 | 1.48 | 5 | 2 | 97.97 | 278.34 | 278.34 |
| -7.2 | Pedalitin | C=CCC1(C(C)CCC)C(=O)NC(=O)NC1=O | 1.35 | 3 | 2 | 75.27 | 238.29 | 238.29 |
| -7.2 | Santamarine | CCCC(C)C1(CC)C(=O)NC(=O)NC1=O | 1.18 | 3 | 2 | 75.27 | 226.28 | 226.28 |
| -7.2 | Methylarbutin | CCC1(CCC(C)C)C(=O)NC(=O)NC1=O | 1.18 | 3 | 2 | 75.27 | 226.28 | 226.28 |
| -7.2 | 6 7-anhydroballotiquinone | CCC(C)C1(CC)C(=O)NC(=O)NC1=O | 0.79 | 3 | 2 | 75.27 | 212.25 | 212.25 |
| -7.2 | 7a-acetoxy-6 7-dihydroicetexone | CCC1(CC)C(=O)NC(=O)N(C)C1=O | 0.5 | 3 | 1 | 66.48 | 198.22 | 198.22 |
| -7.2 | Ballotiquinone | CNC1=Nc2ccc(Cl)cc2C(c2ccccc2)=[N+]([O-])C1 | 2.95 | 3 | 1 | 50.46 | 299.76 | 299.76 |
| -7.2 | 6 7 11 14-tetrahydro-7-oxo-icetexone | N=C(N)c1ccc(CNC(=O)[C@@H]2CCN2C(=O)[C@H](NCC(=O)O)C2CCCCC2)cc1 | 0.81 | 5 | 5 | 148.61 | 429.52 | 429.52 |
| -7.2 | Anastomosine | O=C1CN=C(c2ccccc2Cl)c2cc([N+](=O)[O-])ccc2N1 | 3.04 | 4 | 1 | 84.6 | 315.72 | 315.72 |
| -7.2 | 7 20-dihydroanastomosine | Cc1noc(NS(=O)(=O)c2ccc(N)cc2)c1C | 1.67 | 5 | 2 | 98.22 | 267.31 | 267.31 |
| -7.2 | Icetexone | COc1ccc(NS(=O)(=O)c2ccc(N)cc2)nn1 | 0.87 | 6 | 2 | 107.2 | 280.31 | 280.31 |
| -7.2 | 1 2-anhydroballotiquinone | C=CCC1(CC(C)C)C(=O)NC(=O)NC1=O | 0.96 | 3 | 2 | 75.27 | 224.26 | 224.26 |
| -7.2 | Pinobanksin | CC(=O)NS(=O)(=O)c1ccc(N)cc1 | 0.09 | 4 | 2 | 89.26 | 214.25 | 214.25 |
| -7.2 | 7a-acetoxy-19-hydroxyroyleanone | COc1cc(NS(C)(=O)=O)ccc1Nc1c2ccccc2nc2ccccc12 | 4.51 | 5 | 2 | 80.32 | 393.47 | 393.47 |
| -7.2 | Pinocembrin | C=C(CC)C(=O)c1ccc(OCC(=O)O)c(Cl)c1Cl | 3.61 | 3 | 1 | 63.6 | 303.14 | 303.14 |
| -7.2 | Conacytone | O=C(O)COc1ccc(C(=O)c2cccs2)c(Cl)c1Cl | 3.75 | 4 | 1 | 63.6 | 331.18 | 331.18 |
| -7.2 | chrysin | Cc1ccc(C)c(OCCCC(C)(C)C(=O)O)c1 | 3.57 | 2 | 1 | 46.53 | 250.34 | 250.34 |
| -7.1 | 5,6-Dihydroxy-7,3',4'-Trimethoxyflavone | COc1ccc2[nH]cc(CCNC(C)=O)c2c1 | 1.86 | 2 | 2 | 54.12 | 232.28 | 232.28 |
| -7.1 | 119309-36-3 | NCCCC[C@H](N)C(=O)O | -0.47 | 3 | 3 | 89.34 | 146.19 | 146.19 |
| -7.1 | Alpinetin | Cc1nccn1CC1CCc2c(c3ccccc3n2C)C1=O | 3.13 | 4 | 0 | 39.82 | 293.37 | 293.37 |
| -7.1 | (2R,3R)-alpinone | O=C1CC2(CCCC2)CC(=O)N1CCCCN1CCN(c2ncccn2)CC1 | 2.09 | 6 | 0 | 69.64 | 385.51 | 385.51 |
| -7.1 | Kaempferide | O=C(c1ccc(F)cc1)C1CCN(CCn2c(=O)[nH]c3ccccc3c2=O)CC1 | 2.42 | 5 | 1 | 75.17 | 395.43 | 395.43 |
| -7.1 | Galangin-5-methylether | C[C@](N)(Cc1ccc(O)c(O)c1)C(=O)O | 0.44 | 4 | 4 | 103.78 | 211.22 | 211.22 |
| -7.1 | Pinostrobin | CCc1c(C)[nH]c2c1C(=O)C(CN1CCOCC1)CC2 | 1.96 | 3 | 1 | 45.33 | 276.38 | 276.38 |
| -7.1 | (3R,6R)-dihydroxy-9,7(11)-dien-8-oxoeremophilane | CN1CCc2cccc3c2[C@H]1Cc1ccc(O)c(O)c1-3 | 2.85 | 3 | 2 | 43.7 | 267.33 | 267.33 |
| -7.1 | ISOPETASOL | O=C(CCCN1CCC(O)(c2ccc(Cl)cc2)CC1)c1ccc(F)cc1 | 4.43 | 3 | 1 | 40.54 | 375.87 | 375.87 |
| -7.1 | MEGxp0_000960 | N=C(N)c1ccc(OCCCCCOc2ccc(C(=N)N)cc2)cc1 | 2.88 | 4 | 4 | 118.2 | 340.43 | 340.43 |
| -7.1 | 7-Methoxycoumarin | Nc1ccc(C(=O)NCC(=O)O)cc1 | 0.08 | 3 | 3 | 92.42 | 194.19 | 194.19 |
| -7.1 | Scoparone | CCOC(=O)/C=C(C)/C=C/C=C(C)/C=C/c1c(C)cc(OC)c(C)c1C | 5.65 | 3 | 0 | 35.53 | 354.49 | 354.49 |
| -7.1 | 5128-44-9 | Cc1ccnc2c1NC(=O)c1cccnc1N2C1CC1 | 2.65 | 4 | 1 | 58.12 | 266.3 | 266.3 |
| -7.1 | Isoimperatorin | CCCCCc1cc(O)c2c(c1)OC(C)(C)[C@@H]1CCC(C)=C[C@@H]21 | 5.74 | 2 | 1 | 29.46 | 314.47 | 314.47 |
| -7.1 | Osthole | O=C1c2c(O)ccc(O)c2C(=O)c2c(NCCNCCO)ccc(NCCNCCO)c21 | -0.14 | 10 | 8 | 163.18 | 444.49 | 444.49 |
| -7.1 | methoxsalen | NCCc1ccc(O)c(O)c1 | 0.6 | 3 | 3 | 66.48 | 153.18 | 153.18 |
| -7.1 | Caryophyllene | NC(=O)NO | -0.96 | 2 | 3 | 75.35 | 76.05 | 76.05 |
| -7.1 | Umbelliferone | COc1cc([C@@H]2c3cc4c(cc3[C@H](O)[C@H]3COC(=O)[C@H]23)OCO4)cc(OC)c1OC | 2.41 | 8 | 1 | 92.68 | 414.41 | 414.41 |
| -7.1 | 133164-11-1 | NNC(=O)c1ccncc1 | -0.31 | 3 | 2 | 68.01 | 137.14 | 137.14 |
| -7.1 | Bergapten | O=C1CCC(N2C(=O)c3ccccc3C2=O)C(=O)N1 | 0.09 | 4 | 1 | 83.55 | 258.23 | 258.23 |
| -7.1 | Isoscopoletin | O=C(c1ccccc1)c1ccc2n1CCC2C(=O)O | 2.29 | 3 | 1 | 59.3 | 255.27 | 255.27 |
| -7.1 | scopoletin | CC(C)NCC(O)c1ccc(NS(C)(=O)=O)cc1 | 1.09 | 4 | 3 | 78.43 | 272.37 | 272.37 |
| -7.1 | Brazilin | COc1ccc(Cl)cc1C(=O)NCCc1ccc(S(=O)(=O)NC(=O)NC2CCCCC2)cc1 | 3.64 | 5 | 3 | 113.6 | 494.01 | 494.01 |
| -7.1 | HEMATOXYLIN | CN(CCOc1ccc(NS(C)(=O)=O)cc1)CCc1ccc(NS(C)(=O)=O)cc1 | 1.98 | 6 | 2 | 104.81 | 441.58 | 441.58 |
| -7.1 | Gallic acid | CN(C)/N=N/c1[nH]cnc1C(N)=O | 0.07 | 4 | 2 | 99.73 | 182.19 | 182.19 |
| -7.1 | METHYL GALLATE | Nc1ncnc2c1ncn2[C@@H]1O[C@H](CO)[C@@H](O)[C@H]1O | -1.98 | 9 | 4 | 139.54 | 267.25 | 267.25 |
| -7.1 | DTXSID5020231 | CSc1ccc2c(c1)N(CCC1CCCCN1C)c1ccccc1S2 | 5.89 | 4 | 0 | 6.48 | 370.59 | 370.59 |
| -7.1 | 4-hydroxy cinnamic acid | Cc1c(OCC(F)(F)F)ccnc1C[S+]([O-])c1nc2ccccc2[nH]1 | 3.52 | 4 | 1 | 73.86 | 369.37 | 369.37 |
| -7.1 | Dihydrosporogen AO-1 | CCc1c2c(nc3ccc(OC(=O)N4CCC(N5CCCCC5)CC4)cc13)-c1cc3c(c(=O)n1C2)COC(=O)[C@]3(O)CC | 4.09 | 9 | 1 | 114.2 | 586.69 | 586.69 |
| -7.1 | 2-Hydroxy-6-methoxybenzoic acid | CN1CC[C@]23c4c5ccc(O)c4O[C@H]2[C@@H](O)C=C[C@H]3[C@H]1C5 | 1.2 | 4 | 2 | 52.93 | 285.34 | 285.34 |
| -7.1 | 2-METHOXYBENZOIC ACID | COc1ccc2c3c1O[C@H]1[C@@H](O)C=C[C@H]4[C@@H](C2)N(C)CC[C@@]341 | 1.5 | 4 | 1 | 41.93 | 299.37 | 299.37 |
| -7.1 | (-)-Epicatechin | CN(C)CCCN1c2ccccc2Sc2ccc(Cl)cc21 | 4.89 | 3 | 0 | 6.48 | 318.87 | 318.87 |
| -7.1 | 5,4'-Dihydroxy-3,4,3'-trimethoxybibenzyl | CNCCCN1c2ccccc2CCc2ccccc21 | 3.53 | 2 | 1 | 15.27 | 266.39 | 266.39 |
| -7.1 | erianthridin | CCC1(c2ccc(N)cc2)CCC(=O)NC1=O | 1.35 | 3 | 2 | 72.19 | 232.28 | 232.28 |
| -7.1 | Malbrancheamide | O=C([O-])c1cc(=O)c2c(OCC(O)COc3cccc4oc(C(=O)[O-])cc(=O)c34)cccc2o1.[Na+].[Na+] | 2.11 | 9 | 3 | 173.71 | 468.37 | 512.33 |
| -7.1 | malbrancheamide B | CCN(CC)CCCC(C)Nc1ccnc2cc(Cl)ccc12 | 4.81 | 3 | 1 | 28.16 | 319.88 | 319.88 |
| -7.1 | Trhachylobanoic acid | Fc1ccc([C@@H]2CCNC[C@H]2COc2ccc3c(c2)OCO3)cc1 | 3.33 | 4 | 1 | 39.72 | 329.37 | 329.37 |
| -7.1 | Xanthorrhizol | Cc1ccc(-c2ncc(Cl)cc2-c2ccc(S(C)(=O)=O)cc2)cn1 | 4.18 | 4 | 0 | 59.92 | 358.85 | 358.85 |
| -7.1 | Corymbiferone | CCN(CC)CC(=O)Nc1c(C)cccc1C | 2.58 | 2 | 1 | 32.34 | 234.34 | 234.34 |
| -7 | 4-[2-(3,5-dimethoxyphenyl)ethyl]-2-methoxyphenol | CN1CCN(CC(=O)N2c3ccccc3C(=O)Nc3cccnc32)CC1 | 1.56 | 5 | 1 | 68.78 | 351.41 | 351.41 |
| -7 | Gigantol tetramethyl ether | CCCN(CC)C(CC)C(=O)Nc1c(C)cccc1C | 3.75 | 2 | 1 | 32.34 | 276.42 | 276.42 |
| -7 | 8KZ | C=CCN1CC[C@]23c4c5ccc(O)c4O[C@H]2C(=O)CC[C@@]3(O)[C@H]1C5 | 1.3 | 5 | 2 | 70 | 327.38 | 327.38 |
| -7 | 3'-O-Methylbatatasin III | O=C(c1ccc(OCCN2CCCCC2)cc1)c1c(-c2ccc(O)cc2)sc2cc(O)ccc12 | 6.08 | 6 | 2 | 70 | 473.59 | 473.59 |
| -7 | 3,3',5-Trihydroxybibenzyl | CC/C(=C(\c1ccccc1)c1ccc(OCCN(C)C)cc1)c1ccccc1 | 6 | 2 | 0 | 12.47 | 371.52 | 371.52 |
| -7 | Dihydroresveratrol | CC(C)C[C@H]1C(=O)N2CCC[C@H]2[C@]2(O)O[C@](NC(=O)[C@@H]3C=C4c5cccc6[nH]c(Br)c(c56)C[C@H]4N(C)C3)(C(C)C)C(=O)N12 | 3.19 | 6 | 3 | 118.21 | 654.61 | 654.61 |
| -7 | ephemeranthol A | CC#CCC(C)[C@H](O)/C=C/[C@@H]1[C@H]2C/C(=C/CCCC(=O)O)C[C@H]2C[C@H]1O | 3.54 | 3 | 3 | 77.76 | 360.49 | 360.49 |
| -7 | apigenin | CC[C@@]1(O)C(=O)OCc2c1cc1n(c2=O)Cc2cc3c(CN(C)C)c(O)ccc3nc2-1 | 1.85 | 8 | 2 | 104.89 | 421.45 | 421.45 |
| -7 | Fimbriol A | Cc1nc2n(c(=O)c1CCN1CCC(c3noc4cc(F)ccc34)CC1)CCCC2 | 3.59 | 6 | 0 | 64.16 | 410.49 | 410.49 |
| -7 | 2-(4-Hydroxyphenyl)ethanol | O=C(O)c1cc(=O)c2c(OCC(O)COc3cccc4oc(C(=O)O)cc(=O)c34)cccc2o1 | 2.11 | 9 | 3 | 173.71 | 468.37 | 468.37 |
| -7 | Flavoroseoside | CCN(CC)CCNC(=O)c1cc(Cl)c(N)cc1OC | 2 | 4 | 2 | 67.59 | 299.8 | 299.8 |
| -7 | Methyl 4-hydroxybenzoate | CC(=O)O[C@H]1C(=O)[C@@]2(C)[C@H]([C@H](OC(=O)c3ccccc3)[C@]3(O)C[C@H](OC(=O)[C@H](O)[C@@H](NC(=O)c4ccccc4)c4ccccc4)C(C)=C1C3(C)C)[C@]1(OC(C)=O)CO[C@@H]1C[C@@H]2O | 3.74 | 14 | 4 | 221.29 | 853.92 | 853.92 |
| -7 | Anti-inflammatory agent 57 | CCCCC[C@H](O)/C=C/[C@H]1[C@H](O)CC(=O)[C@@H]1CCCCCCC(=O)O | 3.48 | 4 | 3 | 94.83 | 354.49 | 354.49 |
| -7 | [(9R,10R)-9-hydroxy-8,8-dimethyl-2-oxo-9,10-dihydropyrano[2,3-f]chromen-10-yl] 2-methylpropanoate | Oc1c(Cl)cc(Cl)c(Cl)c1Cc1c(O)c(Cl)cc(Cl)c1Cl | 6.61 | 2 | 2 | 40.46 | 406.91 | 406.91 |
| -7 | Qianhucoumarin A | Oc1c(I)cc(Cl)c2cccnc12 | 3.2 | 2 | 1 | 33.12 | 305.5 | 305.5 |
| -7 | [(9R,10R)-10-hydroxy-8,8-dimethyl-2-oxo-9,10-dihydropyrano[2,3-f]chromen-9-yl] (Z)-2-methylbut-2-enoate | COc1ccc2cccc(CCNC(C)=O)c2c1 | 2.53 | 2 | 1 | 38.33 | 243.31 | 243.31 |
| -7 | Hyuganin D | O=P1(N(CCCl)CCCl)NCCCO1 | 1.88 | 2 | 1 | 41.57 | 261.09 | 261.09 |
| -7 | quianhucoumarin D | CCCNC(=O)NS(=O)(=O)c1ccc(Cl)cc1 | 1.74 | 3 | 2 | 75.27 | 276.75 | 276.75 |
| -7 | (+)-cis-Khellactone | CC(C)(C)NC[C@H](O)COc1nsnc1N1CCOCC1 | 0.5 | 8 | 2 | 79.74 | 316.43 | 316.43 |
| -7 | Selinidin | CC(C)NCC(O)COc1cccc2[nH]ccc12 | 1.91 | 3 | 3 | 57.28 | 248.33 | 248.33 |
| -7 | (R)-O-isobutyroyllomatin | NNc1nncc2ccccc12 | 0.92 | 4 | 2 | 63.83 | 160.18 | 160.18 |
| -7 | Gnaphaliin | CC(N)Cc1ccccc1.O=S(=O)(O)O | 1.58 | 1 | 1 | 26.02 | 135.21 | 233.29 |
| -7 | GNAPHALIIN B | C[C@@H]1[C@H](O)[C@@H](C)/C=C/C=C/C=C/C=C/C=C/C=C/C=C/[C@H](O[C@@H]2O[C@H](C)[C@@H](O)[C@H](N)[C@@H]2O)C[C@@H]2O[C@](O)(C[C@@H](O)C[C@@H](O)[C@H](O)CC[C@@H](O)C[C@@H](O)CC(=O)O[C@H]1C)C[C@H](O)[C@H]2C(=O)O | 0.71 | 17 | 12 | 319.61 | 924.09 | 924.09 |
| -7 | Dendrophenol | NCCc1c[nH]cn1 | -0.09 | 2 | 2 | 54.7 | 111.15 | 111.15 |
| -7 | ephemeranthoquinone | Clc1ccc(COC(Cn2ccnc2)c2ccc(Cl)cc2Cl)c(Cl)c1 | 6.45 | 3 | 0 | 27.05 | 416.13 | 416.13 |
| -7 | Flavanthridin | CC(=O)O[C@@]12CO[C@@H]1C[C@H](O)[C@@]1(C)C(=O)[C@H](O)C3=C(C)[C@@H](OC(=O)[C@H](O)[C@@H](NC(=O)OC(C)(C)C)c4ccccc4)C[C@@](O)([C@@H](OC(=O)c4ccccc4)[C@H]21)C3(C)C | 3.26 | 14 | 5 | 224.45 | 807.89 | 807.89 |
| -7 | Lusianthridin | CC(c1cc2ccccc2s1)N(O)C(N)=O | 2.73 | 3 | 2 | 66.56 | 236.3 | 236.3 |
| -7 | hofmeisterin | CNC(=O)Oc1ccc2c(c1)[C@]1(C)CCN(C)[C@@H]1N2C | 1.77 | 4 | 1 | 44.81 | 275.35 | 275.35 |
| -7 | 3',4',4a',9a'-tetrahydro-6,7'-dimethylspiro[benzofuran-3(2H),2'-pyrano[2,3-b]benzofuran]-2,4a'-diol | Nc1c2c(nc3ccccc13)CCCC2 | 2.7 | 2 | 1 | 38.91 | 198.27 | 198.27 |
| -7 | 2-methyl-4-[(2R)-6-methylhept-5-en-2-yl]phenol | COc1cc2c(cc1OC)C(=O)C(CC1CCN(Cc3ccccc3)CC1)C2 | 4.36 | 4 | 0 | 38.77 | 379.5 | 379.5 |
| -7 | 5-[(3S,3aR,6S,6aR)-3-(3,4-dimethoxyphenyl)-1,3,3a,4,6,6a-hexahydrofuro[3,4-c]furan-6-yl]-4-methyl-1,3-benzodioxole | CC[C@H](C)C(=O)O[C@H]1C[C@@H](C)C=C2C=C[C@H](C)[C@H](CC[C@@H]3C[C@@H](O)CC(=O)O3)[C@H]21 | 4.2 | 5 | 1 | 72.83 | 404.55 | 404.55 |
| -7 | KOBUSIN | O=C(O)/C=C/c1ccc(Cn2ccnc2)cc1 | 2.03 | 3 | 1 | 55.12 | 228.25 | 228.25 |
| -7 | kobusin | CN(C)CCCOc1nn(Cc2ccccc2)c2ccccc12 | 3.42 | 4 | 0 | 30.29 | 309.41 | 309.41 |
| -7 | 2',2' '-dimethoxysesamin | C[S+](C)[O-] | -0.01 | 1 | 0 | 23.06 | 78.14 | 78.14 |
| -7 | Methyl haematommate | CN(C)CCC(c1ccc(Cl)cc1)c1ccccn1 | 3.82 | 2 | 0 | 16.13 | 274.8 | 274.8 |
| -7 | Methyl orsellinate | COc1cc(NC(C)CCCN)c2ncccc2c1 | 2.78 | 4 | 2 | 60.17 | 259.35 | 259.35 |
| -7 | 2AN4H16ZVL | Cc1ccc(Cl)c(Nc2ccccc2C(=O)O)c1Cl | 4.74 | 2 | 2 | 49.33 | 296.15 | 296.15 |
| -7 | Gymnopusin | COc1ccc(CN(CCN(C)C)c2ccccn2)cc1 | 2.66 | 4 | 0 | 28.6 | 285.39 | 285.39 |
| -7 | Hofmeisterin II | O=C1NCCN1CCN1CCC(c2cn(-c3ccc(F)cc3)c3ccc(Cl)cc23)CC1 | 4.63 | 3 | 1 | 40.51 | 440.95 | 440.95 |
| -7 | Thymol derivI | O=NN(CCCl)C(=O)NCCCl | 1.16 | 3 | 1 | 61.77 | 214.05 | 214.05 |
| -7 | 4-hydroxyphenylacetic acid | O=NN(CCCl)C(=O)NC1CCCCC1 | 2.25 | 3 | 1 | 61.77 | 233.7 | 233.7 |
| -7 | Thymol derivIV | O=C(O)CCCc1ccc(N(CCCl)CCCl)cc1 | 3.38 | 2 | 1 | 40.54 | 304.22 | 304.22 |
| -7 | (3S)-8-methoxy-3-methyl-3,4-dihydro-1H-2-benzopyran-1-one | CN1CCC(=C2c3ccccc3C=Cc3ccccc32)CC1 | 4.7 | 1 | 0 | 3.24 | 287.41 | 287.41 |
| -7 | Dihydroflourensic acid | Nc1cc(-c2ccncc2)c[nH]c1=O | 1.02 | 3 | 2 | 71.77 | 187.2 | 187.2 |
| -7 | 1-hydroxy-2-oxoeremo-phil-1(10) 7(11) 8(9)-trien-12(8)-olide | CC(C)N(CCC(C(N)=O)(c1ccccc1)c1ccccn1)C(C)C | 3.36 | 3 | 1 | 59.22 | 339.48 | 339.48 |
| -7 | (2-methoxyphenyl)methyl 2-hydroxybenzoate | O=C(CCCCCCC(=O)Nc1ccccc1)NO | 2.47 | 3 | 3 | 78.43 | 264.32 | 264.32 |
| -7 | Benzyl 2,6-dimethoxybenzoate | CCn1cc(C(=O)O)c(=O)c2cc(F)c(N3CCN(C)CC3)cc21 | 1.61 | 5 | 1 | 65.78 | 333.36 | 333.36 |
| -7 | Benzyl 2-hydroxy-6-methoxybenzoate | CC(C)Cc1ccc(C(C)C(=O)O)cc1 | 3.07 | 1 | 1 | 37.3 | 206.28 | 206.28 |
| -7 | Benzyl 6-hydroxy-2,3-dimethoxybenzoate | COc1ccc2c(C(=O)c3ccc(Cl)cc3)c(C)n(CC(=O)O)c2c1 | 3.93 | 4 | 1 | 68.53 | 357.79 | 357.79 |
| -7 | 9-deoxy-PF1233 B | CN1C(=O)CN=C(c2ccccc2F)c2cc([N+](=O)[O-])ccc21 | 2.55 | 4 | 0 | 75.81 | 313.29 | 313.29 |
| -7 | 9-deoxy-PF1233 A | O=C1CN=C(c2ccccc2)c2cc([N+](=O)[O-])ccc2N1 | 2.38 | 4 | 1 | 84.6 | 281.27 | 281.27 |
| -7 | gedunin | COc1ccc(OC)c(C(O)C(C)N)c1 | 1.08 | 4 | 2 | 64.71 | 211.26 | 211.26 |
| -7 | cadalen-15-oic acid | CC(C)c1cccc(C(C)C)c1O | 3.64 | 1 | 1 | 20.23 | 178.27 | 178.27 |
| -7 | 7-Hydroxycadalene | CN1C(C(=O)Nc2ccccn2)=C(O)c2ccccc2S1(=O)=O | 1.58 | 5 | 2 | 99.6 | 331.35 | 331.35 |
| -6.9 | 7-Prenyloxycoumarin | CO/N=C(\C(=O)N[C@@H]1C(=O)N2C(C(=O)O)=CCS[C@H]12)c1csc(N)n1 | -0.56 | 9 | 3 | 147.21 | 383.41 | 383.41 |
| -6.9 | (2S)-2,6-dihydroxy-4,7-dimethyl-2-propan-2-ylnaphthalen-1-one | CCCCC1C(=O)N(c2ccccc2)N(c2ccccc2)C1=O | 3.79 | 2 | 0 | 40.62 | 308.38 | 308.38 |
| -6.9 | 72943-94-3 | CC[C@H]1OC(=O)[C@H](C)[C@@H](O[C@H]2C[C@@](C)(OC)[C@@H](O)[C@H](C)O2)[C@H](C)[C@@H](O[C@@H]2O[C@H](C)C[C@H](N(C)C)[C@H]2O)[C@](C)(O)C[C@@H](C)CN(C)[C@H](C)[C@@H](O)[C@]1(C)O | 1.9 | 14 | 5 | 180.08 | 749 | 749 |
| -6.9 | (4R)-4-hydroxy-4,7-dimethyl-2-propan-2-ylnaphthalen-1-one | CC1(C)S[C@@H]2[C@H](/N=C/N3CCCCCC3)C(=O)N2[C@H]1C(=O)O | 1.41 | 4 | 1 | 73.21 | 325.43 | 325.43 |
| -6.9 | (5R,8S)-5-hydroxy-5-methyl-8-propan-2-yl-7,8-dihydro-6H-naphthalene-2-carboxylic acid | CCCN1C[C@H](CSC)C[C@@H]2c3cccc4[nH]cc(c34)C[C@H]21 | 4.27 | 2 | 1 | 19.03 | 314.5 | 314.5 |
| -6.9 | 5alpha,7alpha,10betaH-3-Patchoulen-2-one | CC[C@H]1OC(=O)[C@H](C)[C@@H](O[C@H]2C[C@@](C)(OC)[C@@H](O)[C@H](C)O2)[C@H](C)[C@@H](O[C@@H]2O[C@H](C)C[C@H](N(C)C)[C@H]2O)[C@](C)(O)C[C@@H](C)C(=O)[C@H](C)[C@@H](O)[C@]1(C)O | 1.79 | 14 | 5 | 193.91 | 733.94 | 733.94 |
| -6.9 | USEWIKYKYDMBSN-XTELSCJUSA- | CCCCCCCN(CC)CCCC(O)c1ccc(NS(C)(=O)=O)cc1 | 4.16 | 4 | 2 | 69.64 | 384.59 | 384.59 |
| -6.9 | Xanthyletin | CC(=O)[C@H]1CC[C@H]2[C@@H]3CCC4=CC(=O)CC[C@]4(C)[C@H]3CC[C@]12C | 4.72 | 2 | 0 | 34.14 | 314.47 | 314.47 |
| -6.9 | Dimethyl allyl xanthyletin | O=c1[nH]c2ccccc2n1CCCN1CCN(C(c2ccccc2)c2ccccc2)CC1 | 4.13 | 4 | 1 | 44.27 | 426.56 | 426.56 |
| -6.9 | Chalepensin | CN1CCC(=C2c3ccccc3CC(=O)c3sccc32)CC1 | 4.01 | 3 | 0 | 20.31 | 309.43 | 309.43 |
| -6.9 | (2R)-2-prop-1-en-2-yl-2,3-dihydrofuro[3,2-g]chromen-7-one | CCN(CC)CCNC(=O)c1c(C)[nH]c(/C=C2\C(=O)Nc3ccc(F)cc32)c1C | 3.33 | 3 | 3 | 77.23 | 398.48 | 398.48 |
| -6.9 | 2-isopropyl-7H-furo[3,2-g]chromen-7-one | Oc1ccc(O)cc1 | 1.1 | 2 | 2 | 40.46 | 110.11 | 110.11 |
| -6.9 | (2R)-2-(2-hydroxypropan-2-yl)-6-(2-methylbut-3-en-2-yl)-2,3-dihydrofuro[3,2-g]chromen-7-one | CC(=O)O | 0.09 | 1 | 1 | 37.3 | 60.05 | 60.05 |
| -6.9 | Caryophyllene oxide | O=C(O)c1ccccc1 | 1.38 | 1 | 1 | 37.3 | 122.12 | 122.12 |
| -6.9 | (1S 3Z 7E 11S 12S)-(+)-verticilla-3 7-dien-12 20-diol | Nc1ccc(C(=O)O)cc1 | 0.97 | 2 | 2 | 63.32 | 137.14 | 137.14 |
| -6.9 | Shornephine A | O=C(O)C(Cl)(Cl)Cl | 1.44 | 1 | 1 | 37.3 | 163.39 | 163.39 |
| -6.9 | Cacalol | Oc1ccccc1 | 1.39 | 1 | 1 | 20.23 | 94.11 | 94.11 |
| -6.9 | Cacalone | COc1ccc(O)cc1 | 1.4 | 2 | 1 | 29.46 | 124.14 | 124.14 |
| -6.9 | lophodiol A | Clc1ccccc1C(c1ccccc1)(c1ccccc1)n1ccnc1 | 5.38 | 2 | 0 | 17.82 | 344.85 | 344.85 |
| -6.9 | 15,16-epoxylophotoxin | CO[C@@]12[C@H](COC(N)=O)C3=C(C(=O)C(C)=C(N)C3=O)N1C[C@@H]1N[C@@H]12 | -1.65 | 8 | 3 | 146.89 | 334.33 | 334.33 |
| -6.9 | 17-acetoxylophotoxin | CCO | 0 | 1 | 1 | 20.23 | 46.07 | 46.07 |
| -6.9 | Esculetin | C=CCOc1ccccc1OCC(O)CNC(C)C | 1.99 | 4 | 2 | 50.72 | 265.35 | 265.35 |
| -6.9 | (-)-Rubifolide | OC(Cn1cncn1)(Cn1cncn1)c1ccc(F)cc1F | 0.74 | 7 | 1 | 81.65 | 306.28 | 306.28 |
| -6.9 | Patuletin | CC1=C(/C=C/C(C)=C/C=C/C(C)=C\C(=O)O)C(C)(C)CCC1 | 5.6 | 1 | 1 | 37.3 | 300.44 | 300.44 |
| -6.9 | 8-Acetyl-7-hydroxycoumarin | COc1cc(C(C)=O)ccc1OCCCN1CCC(c2noc3cc(F)ccc23)CC1 | 4.83 | 6 | 0 | 64.8 | 426.49 | 426.49 |
| -6.9 | 6-acetyl-7-methoxychromen-2-one | CCCCC[C@H](O)/C=C/[C@H]1[C@H](O)CC(=O)[C@@H]1C/C=C\CCCC(=O)O | 3.25 | 4 | 3 | 94.83 | 352.47 | 352.47 |
| -6.9 | lopholide | CN(C)C(=O)Oc1cccc([N+](C)(C)C)c1 | 1.94 | 2 | 0 | 29.54 | 223.3 | 223.3 |
| -6.9 | lophodiol B | CCOc1ccccc1OC(c1ccccc1)C1CNCCO1 | 3.19 | 4 | 1 | 39.72 | 313.4 | 313.4 |
| -6.9 | Lophotoxin | CN(C)CCCC1(c2ccc(F)cc2)OCc2cc(C#N)ccc21 | 3.81 | 3 | 0 | 36.26 | 324.4 | 324.4 |
| -6.9 | axinisothiocyanate M | CC[C@@H]1C(=O)OC[C@@H]1Cc1cncn1C | 1.16 | 4 | 0 | 44.12 | 208.26 | 208.26 |
| -6.9 | axinisothiocyanate N | COc1cc2c(c(OC)c1OC)-c1ccc(OC)c(=O)cc1[C@@H](NC(C)=O)CC2 | 2.87 | 6 | 1 | 83.09 | 399.44 | 399.44 |
| -6.9 | 3-[(3R)-7-hydroxy-3,4-dihydro-2H-chromen-3-yl]-6-methoxybenzene-1,2-diol | C#Cc1cccc(Nc2ncnc3cc(OCCOC)c(OCCOC)cc23)c1 | 3.41 | 7 | 1 | 74.73 | 393.44 | 393.44 |
| -6.9 | 57128-11-7 | CS(=O)(=O)CCNCc1ccc(-c2ccc3ncnc(Nc4ccc(OCc5cccc(F)c5)c(Cl)c4)c3c2)o1 | 6.14 | 8 | 2 | 106.35 | 581.07 | 581.07 |
| -6.9 | 4-Hydroxyhomopterocarpin | O=C(CCCN1CCC(O)(c2cccc(C(F)(F)F)c2)CC1)c1ccc(F)cc1 | 4.79 | 3 | 1 | 40.54 | 409.42 | 409.42 |
| -6.9 | (+)-Vestitol | NC(=O)N1c2ccccc2C=Cc2ccccc21 | 3.39 | 1 | 1 | 46.33 | 236.27 | 236.27 |
| -6.9 | Meliotocarpan D | COC(=O)[C@@H]1[C@H]2C[C@H]3c4[nH]c5ccccc5c4CCN3C[C@@H]2CC[C@@H]1O | 2.65 | 4 | 2 | 65.56 | 354.45 | 354.45 |
| -6.9 | (2S)-7-hydroxyflavanone | CCCC(CCC)C(=O)O | 2.29 | 1 | 1 | 37.3 | 144.21 | 144.21 |
| -6.9 | Elemicin | CC1CN(c2cc3c(cc2F)c(=O)c(C(=O)O)cn3-c2ccc(F)cc2F)CCN1 | 2.9 | 5 | 2 | 74.57 | 417.39 | 417.39 |
| -6.9 | Jacaranone | O=C(Cn1ccnc1[N+](=O)[O-])NCc1ccccc1 | 1.11 | 5 | 1 | 90.06 | 260.25 | 260.25 |
| -6.9 | (-)-syringaresinol | CC(=O)N(O)CCCCCNC(=O)CCC(=O)N(O)CCCCCNC(=O)CCC(=O)N(O)CCCCCN | 0.92 | 9 | 6 | 205.84 | 560.69 | 560.69 |
| -6.9 | (5S,7R,8R,9R,10S)-(-)-7,8-seco-7,8-oxacassa-13,15-diene-7,17-diol | Cc1cccc(C)c1OCC(C)N | 2.03 | 2 | 1 | 35.25 | 179.26 | 179.26 |
| -6.9 | (5S,7R, 8R,9R,10S)-(-)-7,8-seco-7,8-oxacassa-13,15-dien-7-ol | N[C@H](Cc1cc(I)c(Oc2cc(I)c(O)c(I)c2)c(I)c1)C(=O)O | 4.56 | 4 | 3 | 92.78 | 776.87 | 776.87 |
| -6.9 | notoamide B | Cc1c(C(=O)NN2CCCCC2)nn(-c2ccc(Cl)cc2Cl)c1-c1ccc(Cl)cc1 | 5.94 | 4 | 1 | 50.16 | 463.8 | 463.8 |
| -6.9 | [(1S)-1-[(2S,5R)-5-[(S)-hydroxy-[(2R)-6-oxo-2,3-dihydropyran-2-yl]methyl]oxolan-2-yl]ethyl] (Z)-3-(4-methoxyphenyl)prop-2-enoate | CN1Cc2c(N)cccc2C(c2ccccc2)C1 | 2.85 | 2 | 1 | 29.26 | 238.33 | 238.33 |
| -6.9 | (3R)-5-[(1R,2R,4aS,8aS)-2-hydroxy-2,5,5,8a-tetramethyl-3,4,4a,6,7,8-hexahydro-1H-naphthalen-1-yl]-3-methylpentanoic acid | CCn1cc(C(=O)O)c(=O)c2cc(F)c(N3CCNC(C)C3)c(F)c21 | 1.8 | 5 | 2 | 74.57 | 351.35 | 351.35 |
| -6.9 | [(1S)-1-[(2S,5R)-5-[(S)-hydroxy-[(2R)-6-oxo-2,3-dihydropyran-2-yl]methyl]oxolan-2-yl]ethyl] (Z)-3-(4-hydroxyphenyl)prop-2-enoate | COC1=CC(=O)C[C@@H](C)[C@]12Oc1c(Cl)c(OC)cc(OC)c1C2=O | 2.81 | 6 | 0 | 71.06 | 352.77 | 352.77 |
| -6.9 | [(1S)-1-[(2S,5R)-5-[(S)-hydroxy-[(2R)-6-oxo-2,3-dihydropyran-2-yl]methyl]oxolan-2-yl]ethyl] (E)-3-(4-methoxyphenyl)prop-2-enoate | CC(C(=O)O)c1ccc(-c2ccccc2)c(F)c1 | 3.68 | 1 | 1 | 37.3 | 244.26 | 244.26 |
| -6.9 | 28590-40-1 | CN(C)CCCN1c2ccccc2Sc2ccccc21 | 4.24 | 3 | 0 | 6.48 | 284.43 | 284.43 |
| -6.9 | Podophyllotoxin acetate | CCOC(=O)C(C)(C)Oc1ccc(Cl)cc1 | 3.06 | 3 | 0 | 35.53 | 242.7 | 242.7 |
| -6.8 | Chrysoeriol | CC(=O)Nc1ccc(O)cc1 | 1.35 | 2 | 2 | 49.33 | 151.16 | 151.16 |
| -6.8 | [(1S)-1-[(2S,5R)-5-[(S)-hydroxy-[(2R)-6-oxo-2,3-dihydropyran-2-yl]methyl]oxolan-2-yl]ethyl] (E)-3-(3,4-dihydroxyphenyl)prop-2-enoate | O=C(O)CCC(=O)c1ccc(-c2ccccc2)cc1 | 3.4 | 2 | 1 | 54.37 | 254.28 | 254.28 |
| -6.8 | LUPINIFOLINOL | Cn1c(=O)c2c(ncn2C)n(C)c1=O | -1.03 | 6 | 0 | 61.82 | 194.19 | 194.19 |
| -6.8 | 2-[[(8S)-6,15,16-trimethoxy-12-oxo-11-oxa-10-azatetracyclo[7.7.1.02,7.013,17]heptadeca-1(17),2(7),3,5,9,13,15-heptaen-8-yl]sulfanyl]acetamide | CCOc1ccc(NC(C)=O)cc1 | 2.04 | 2 | 1 | 38.33 | 179.22 | 179.22 |
| -6.8 | Aristololactam | OCCN1CCN(CCCN2c3ccccc3Sc3ccc(Cl)cc32)CC1 | 3.94 | 5 | 1 | 29.95 | 403.98 | 403.98 |
| -6.8 | Humul-7-en-1 4 11-triol | O=C1Nc2ccc(Cl)cc2C(c2ccccc2)=NC1O | 2.45 | 3 | 2 | 61.69 | 286.72 | 286.72 |
| -6.8 | Arborinine | CC1=C(CC(=O)O)c2cc(F)ccc2/C1=C\c1ccc([S+](C)[O-])cc1 | 4.37 | 2 | 1 | 60.36 | 356.42 | 356.42 |
| -6.8 | 17014-60-7 | O=C(O)Cc1ccccc1Oc1ccc(Cl)cc1Cl | 4.41 | 2 | 1 | 46.53 | 297.14 | 297.14 |
| -6.8 | 1-hydroxy-3-methoxy-10-methylacridin-9-one | Clc1ccc(C(c2ccc(Cl)cc2)C(Cl)(Cl)Cl)cc1 | 6.5 | 0 | 0 | 0 | 354.49 | 354.49 |
| -6.8 | 4-methoxy-1-methylquinolin-2-one | c1ccc2c(CCC3CCNCC3)c[nH]c2c1 | 3.1 | 1 | 2 | 27.82 | 228.34 | 228.34 |
| -6.8 | Graveoline | CCN(CC)CCOC(=O)c1ccc(N)cc1 | 1.77 | 4 | 1 | 55.56 | 236.31 | 236.31 |
| -6.8 | beta-Peltatin A methyl ether | CN(C)CCCN1c2ccccc2Sc2ccc(C(F)(F)F)cc21 | 5.26 | 3 | 0 | 6.48 | 352.43 | 352.43 |
| -6.8 | 5? -desmethoxy-?-peltatin-A-methylether | CC(C(=O)O)c1cccc(C(=O)c2ccccc2)c1 | 3.11 | 2 | 1 | 54.37 | 254.29 | 254.29 |
| -6.8 | desoxypodophyllotoxin | O=C1CN(/N=C/c2ccc([N+](=O)[O-])o2)C(=O)N1 | 0.07 | 6 | 1 | 118.05 | 238.16 | 238.16 |
| -6.8 | burseranin | CC(C(=O)O)c1ccc(N2Cc3ccccc3C2=O)cc1 | 3.04 | 2 | 1 | 57.61 | 281.31 | 281.31 |
| -6.8 | 7? 8 ?-dehydropodophyllotoxin | Cl[C@H]1[C@H](Cl)[C@@H](Cl)[C@@H](Cl)[C@H](Cl)[C@H]1Cl | 3.64 | 0 | 0 | 0 | 290.83 | 290.83 |
| -6.8 | 7? 8?-dehydroacethyl podophyllotxin | O=C(O)c1cccnc1 | 0.78 | 2 | 1 | 50.19 | 123.11 | 123.11 |
| -6.8 | (-)-(13E)-labd-13-ene-8a 15-diol | COc1cccc(OC)c1C(=O)N[C@@H]1C(=O)N2[C@@H]1SC(C)(C)[C@@H]2C(=O)O | 0.95 | 6 | 2 | 105.17 | 380.42 | 380.42 |
| -6.8 | (+)-(Z)-labda-8 13-diene-15 16-diol | CC(Cl)(Cl)Cl | 2.38 | 0 | 0 | 0 | 133.41 | 133.41 |
| -6.8 | (1R 4aS 8aS)-1-((R E)-2 5-dihydroxy-3-(hydroxymethyl)pent-3-en-1-yl)-5 5 8a-trimethyloctahydronaphthalen-2(1H)-one | CCOC(=O)c1ccc(N)cc1 | 1.45 | 3 | 1 | 52.32 | 165.19 | 165.19 |
| -6.8 | (1R 4aS 8aS)-1-((R E)-2 5-dihydroxy-3-methylpent-3-en-1-yl)-5 5 8a-trimethyloctahydronaphthalen-2(1H)-one | C[C@H](N[C@@H](CCc1ccccc1)C(=O)O)C(=O)N1CCC[C@H]1C(=O)O | 1.13 | 4 | 3 | 106.94 | 348.4 | 348.4 |
| -6.8 | (1R 4aS 8aS)-1-((E)-5-hydroxy-3-methyl-2-oxopent-3-en-1-yl)-5 5 8a-trimethyloctahydronaphthalen-2(1H)-one | CCOC(=O)[C@H](CCc1ccccc1)N[C@@H](C)C(=O)N1CCC[C@H]1C(=O)O | 1.6 | 5 | 2 | 95.94 | 376.45 | 376.45 |
| -6.8 | (1R 4aS 8aS)-5 5 8a-trimethyl-1-((3-methylfuran-2-yl)methyl)octahydronaphthalen-2(1H)-one | O=C1Nc2ccc(Cl)cc2C(c2ccccc2Cl)=NC1O | 3.1 | 3 | 2 | 61.69 | 321.16 | 321.16 |
| -6.8 | (1R 4aS 8aS)-1-((S E)-5-hydroxy-3-methyl-2-(((2R 3R 4S 5S 6R)-3 4 5-trihydroxy-6-(hydroxymethyl)tetrahydro-2H-pyran-2-yl)oxy)pent-3-en-1-yl)-5 5 8a-trimethyloctahydronaphthalen-2(1H)-one | O=C1CN=C(c2ccccn2)c2cc(Br)ccc2N1 | 2.63 | 3 | 1 | 54.35 | 316.16 | 316.16 |
| -6.8 | (1R 4aS 8aS)-5 5 8a-trimethyl-1-((2R)-2 4 5-trihydroxy-3-oxopentyl)octahydronaphthalen-2(1H)-one | ClC=C(Cl)Cl | 2.5 | 0 | 0 | 0 | 131.39 | 131.39 |
| -6.8 | (R)-4-hydroxy-5-((1R 4aS 8aS)-5 5 8a-trimethyl-2-oxodecahydronaphthalen-1-yl)pentane-2 3-dione | c1ccc(-c2ccc(C(c3ccccc3)n3ccnc3)cc2)cc1 | 5.19 | 2 | 0 | 17.82 | 310.4 | 310.4 |
| -6.8 | 1 2-dihydroxy-5-((1R 2R 4aS 8aS)-2-hydroxy-2 5 5 8a-tetramethyldecahydronaphthalen-1-yl)pentan-3-one | CC(C)O | 0.39 | 1 | 1 | 20.23 | 60.1 | 60.1 |
| -6.8 | 6-((1R)-1-hydroxy-1-((8R 9S 10S 13R 14S)-14-hydroxy-10 13-dimethyl-1-oxo-4 5 6 7 8 9 10 11 12 13 14 15 16 17-tetradecahydro-1H-cyclopenta[a]phenanthren-17-yl)ethyl)-3 4-dimethyl-5 6-dihydro-2H-pyran-2-one | Cc1c(F)c(N2CCNC(C)C2)cc2c1c(=O)c(C(=O)O)cn2C1CC1 | 2.28 | 5 | 2 | 74.57 | 359.4 | 359.4 |
| -6.8 | 3,7-Di-O-methylquercetin | CC(C)(C)NC(=O)[C@@H]1C[C@@H]2CCCC[C@@H]2CN1C[C@@H](O)[C@H](Cc1ccccc1)NC(=O)[C@H](CC(N)=O)NC(=O)c1ccc2ccccc2n1 | 3.09 | 7 | 5 | 166.75 | 670.86 | 670.86 |
| -6.8 | 3,4-Dimethoxybenzoic acid | CC(C)(C)NC(=O)[C@@H]1CN(Cc2cccnc2)CCN1C[C@@H](O)C[C@@H](Cc1ccccc1)C(=O)N[C@H]1c2ccccc2C[C@H]1O | 2.87 | 7 | 4 | 118.03 | 613.8 | 613.8 |
| -6.8 | areolal | Cc1c(O)cccc1C(=O)N[C@@H](CSc1ccccc1)[C@H](O)CN1C[C@H]2CCCC[C@H]2C[C@H]1C(=O)NC(C)(C)C | 4.75 | 6 | 4 | 101.9 | 567.8 | 567.8 |
| -6.8 | Ciquitin A | CC(C)CN(C[C@@H](O)[C@H](Cc1ccccc1)NC(=O)O[C@H]1CCOC1)S(=O)(=O)c1ccc(N)cc1 | 2.4 | 7 | 3 | 131.19 | 505.64 | 505.64 |
| -6.8 | Ciquitin B | Nc1nc(N)c2nc(-c3ccccc3)c(N)nc2n1 | 0.83 | 7 | 3 | 129.62 | 253.27 | 253.27 |
| -6.8 | Ciquitin C | CC(C)NCC(O)c1ccc2ccccc2c1 | 2.87 | 2 | 2 | 32.26 | 229.32 | 229.32 |
| -6.8 | (3S)-hydroxy-3' 4'-dimethoxy-L-phenylalanine | Cc1ccc(-c2cc(C(F)(F)F)nn2-c2ccc(S(N)(=O)=O)cc2)cc1 | 3.51 | 4 | 1 | 77.98 | 381.38 | 381.38 |
| -6.8 | (-)-Germacrene D | COc1cc(NCc2ccc3nc(N)nc(N)c3c2C)cc(OC)c1OC | 2.74 | 8 | 3 | 117.54 | 369.43 | 369.43 |
| -6.8 | 62458-61-1 | Oc1ccc(C2CNCCc3c2cc(O)c(O)c3Cl)cc1 | 2.73 | 4 | 4 | 72.72 | 305.76 | 305.76 |
| -6.8 | Encecalin | CCCN(CCC)CCc1cccc2c1CC(=O)N2 | 2.85 | 2 | 1 | 32.34 | 260.38 | 260.38 |
| -6.8 | 19013-07-1 | CC1=CC(=O)c2ccccc2C1=O | 2.01 | 2 | 0 | 34.14 | 172.18 | 172.18 |
| -6.8 | (S)-O-Methylencecalinol | C#CC(O)(/C=C/Cl)CC | 1.51 | 1 | 1 | 20.23 | 144.6 | 144.6 |
| -6.8 | 2-(3-(2-methylbut-3-en-2-yl)-2-oxo-2 6 7 8-tetrahydrocyclopenta[g]chromen-7-yl)propan-2-yl acetate | CC(C)(C)c1ccc(C(O)CCCN2CCC(C(O)(c3ccccc3)c3ccccc3)CC2)cc1 | 6.45 | 3 | 2 | 43.7 | 471.69 | 471.69 |
| -6.8 | 4-HYDROXYBENZOIC ACID | CN1CC[C@]23CCCC[C@H]2[C@H]1Cc1ccc(O)cc13 | 3.08 | 2 | 1 | 23.47 | 257.38 | 257.38 |
| -6.8 | 2-Propenoic acid, 3-phenyl- | CC(C)Nc1cccnc1N1CCN(C(=O)c2cc3cc(NS(C)(=O)=O)ccc3[nH]2)CC1 | 2.72 | 6 | 3 | 110.43 | 456.57 | 456.57 |
| -6.8 | salicylic acid | CCOCCn1c(N2CCCN(C)CC2)nc2ccccc21 | 2.21 | 5 | 0 | 33.53 | 302.42 | 302.42 |
| -6.8 | Vanillic acid | CCc1ccc(CCOc2ccc(CC3SC(=O)NC3=O)cc2)nc1 | 3.16 | 5 | 1 | 68.29 | 356.45 | 356.45 |
| -6.8 | vanillin | CCC(=O)N(c1ccccc1)C1CCN(CCc2ccccc2)CC1 | 4.14 | 2 | 0 | 23.55 | 336.48 | 336.48 |
| -6.8 | 4-METHOXYBENZOIC ACID | CN(CCOc1ccc(CC2SC(=O)NC2=O)cc1)c1ccccn1 | 2.49 | 6 | 1 | 71.53 | 357.44 | 357.44 |
| -6.8 | Mirabilin B | CCCCC1(CCCC)CS(=O)(=O)c2ccc(N(C)C)cc2[C@@H](c2ccc(OCc3ccc(C[N+]45CCN(CC4)CC5)cc3)cc2)[C@H]1O.[Cl-] | 6.62 | 6 | 1 | 70.08 | 674.97 | 710.42 |
| -6.8 | Perezone | CC(Oc1c(Cl)cccc1Cl)C1=NCCN1 | 2.76 | 3 | 1 | 33.62 | 259.14 | 259.14 |
| -6.8 | 2211-21-4 | Cc1ccc(N(CC2=NCCN2)c2cccc(O)c2)cc1 | 2.84 | 4 | 2 | 47.86 | 281.36 | 281.36 |
| -6.8 | 8beta-d-glucopyranosyloxy-4-methoxy-5-methyl-coumarin | c1cc(CN2CCCNCCNCCCNCC2)ccc1CN1CCCNCCNCCCNCC1 | 0.42 | 8 | 6 | 78.66 | 502.8 | 502.8 |
| -6.8 | Benzomalvin-A | Cc1cccc(N2CC(CO)OC2=O)c1 | 1.31 | 3 | 1 | 49.77 | 207.23 | 207.23 |
| -6.8 | Quinolactacin A1 | Cc1cnc(NC(=O)C2=C(O)c3ccccc3S(=O)(=O)N2C)s1 | 1.95 | 6 | 2 | 99.6 | 351.41 | 351.41 |
| -6.8 | Quinolactacin A2 | CC(=O)N[C@@H](CS)C(=O)O | -0.49 | 3 | 3 | 66.4 | 163.2 | 163.2 |
| -6.8 | quinolonimide | NCC(=O)CCC(=O)O | -0.62 | 3 | 2 | 80.39 | 131.13 | 131.13 |
| -6.8 | Quinoline alkaloid | NCCS | -0.13 | 2 | 2 | 26.02 | 77.15 | 77.15 |
| -6.8 | benzaldehyde | O=C1CC[C@@]2(O)[C@H]3Cc4ccc(O)c5c4[C@@]2(CCN3CC2CC2)[C@H]1O5 | 1.53 | 5 | 2 | 70 | 341.41 | 341.41 |
| -6.8 | Quininae | COc1cc(C(=O)NS(=O)(=O)c2ccccc2C)ccc1Cc1cn(C)c2ccc(NC(=O)OC3CCCC3)cc12 | 5.7 | 7 | 2 | 115.73 | 575.69 | 575.69 |
| -6.8 | ABSINTHIN | C/C=C/C1=C(C(=O)O)N2C(=O)[C@@H](NC(=O)[C@H](N)c3ccc(O)cc3)[C@H]2SC1 | 0.71 | 6 | 4 | 132.96 | 389.43 | 389.43 |
| -6.8 | Aristolochic acid | CS(=O)(=O)c1ccc(C2=C(c3ccccc3)C(=O)OC2)cc1 | 2.56 | 4 | 0 | 60.44 | 314.36 | 314.36 |
| -6.8 | (1R,3S,6S,10S,11S)-3,12-dimethyl-7-methylidene-2,9-dioxatetracyclo[9.3.0.0^{1,3}.0^{6,10}]tetradec-12-en-8-one | COc1nc(C)nc(Cl)c1NC1=NCCN1 | 0.82 | 6 | 2 | 71.43 | 241.68 | 241.68 |
| -6.8 | coumarin | COc1ccc(Cc2nccc3cc(OC)c(OC)cc23)cc1OC | 3.86 | 5 | 0 | 49.81 | 339.39 | 339.39 |
| -6.8 | Artemorin | CCN(CC)CCNC(=O)c1ccc(N)cc1.Cl | 1.34 | 3 | 2 | 58.36 | 235.33 | 271.79 |
| -6.8 | Cascarillin | CN1C[C@H](CNC(=O)OCc2ccccc2)C[C@@H]2c3cccc4c3c(cn4C)C[C@H]21 | 4.06 | 4 | 1 | 46.5 | 403.53 | 403.53 |
| -6.8 | papaverine | CCCCC(C)(O)C/C=C/[C@H]1[C@H](O)CC(=O)[C@@H]1CCCCCCC(=O)OC | 3.95 | 5 | 2 | 83.83 | 382.54 | 382.54 |
| -6.8 | parthenolide | CC(C)(N)C(=O)N[C@H](Cc1c[nH]c2ccccc12)C(=O)N[C@H](Cc1c[nH]c2ccccc12)NC=O | 1.84 | 4 | 6 | 144.9 | 474.57 | 474.57 |
| -6.8 | noscapine | CCOC(=O)C1(c2ccccc2)CCN(C)CC1 | 2.21 | 3 | 0 | 29.54 | 247.34 | 247.34 |
| -6.8 | Benzamide | Cc1ccccc1-n1c(C)nc2ccccc2c1=O | 3 | 3 | 0 | 34.89 | 250.3 | 250.3 |
| -6.8 | azathioprine | CC(C)(Sc1cc(C(C)(C)C)c(O)c(C(C)(C)C)c1)Sc1cc(C(C)(C)C)c(O)c(C(C)(C)C)c1 | 9.91 | 4 | 2 | 40.46 | 516.86 | 516.86 |
| -6.8 | quassin | CC(C(=O)O)c1ccc(CC2CCCC2=O)cc1 | 2.79 | 2 | 1 | 54.37 | 246.31 | 246.31 |
| -6.8 | diphenhydramine | NCCNCCNCCN | -1.92 | 4 | 4 | 76.1 | 146.24 | 146.24 |
| -6.8 | Thiocarbanilide | Cc1cc(CC(=O)O)n(C)c1C(=O)c1ccc(Cl)cc1 | 2.85 | 3 | 1 | 59.3 | 291.73 | 291.73 |
| -6.8 | sodium benzoate | COc1cc2nc(N3CCN(C(=O)C4CCCO4)CC3)nc(N)c2cc1OC | 1.06 | 8 | 1 | 103.04 | 387.44 | 387.44 |
| -6.8 | thiamine | C[C@H](N)Cc1ccccc1 | 1.58 | 1 | 1 | 26.02 | 135.21 | 135.21 |
| -6.8 | chloramphenicol | Cc1cc2c(cc1CC(=O)c1sccc1S(=O)(=O)Nc1onc(C)c1Cl)OCO2 | 3.96 | 8 | 1 | 107.73 | 454.91 | 454.91 |
| -6.8 | MFCD00210576 | NC(=O)c1cnccn1 | -0.42 | 3 | 1 | 68.87 | 123.11 | 123.11 |
| -6.8 | (1R,9R,10S)-4-methoxy-17-methyl-17-azatetracyclo[7.5.3.0^{1,10}.0^{2,7}]heptadeca-2,4,6-triene | CC(C)C(=O)c1c(C(C)C)nn2ccccc12 | 3.3 | 3 | 0 | 34.37 | 230.31 | 230.31 |
| -6.8 | SODIUM CYCLAMATE | CC(C)(C)NC(=O)[C@@H]1C[C@@H]2CCCC[C@@H]2CN1C[C@@H](O)[C@H](Cc1ccccc1)NC(=O)[C@H](CC(N)=O)NC(=O)c1ccc2ccccc2n1.CS(=O)(=O)O | 3.09 | 7 | 5 | 166.75 | 670.86 | 766.96 |
| -6.8 | picrotoxinin | O=c1[nH]c(=O)n(C2CCCO2)cc1F | -0.02 | 4 | 1 | 64.09 | 200.17 | 200.17 |
| -6.8 | beta-Thujone | CC1(C)S[C@@H]2[C@H](NC(=O)COc3ccccc3)C(=O)N2[C@H]1C(=O)O | 0.7 | 5 | 2 | 95.94 | 350.4 | 350.4 |
| -6.8 | dapsone | CC(=O)OCC1=C(C(=O)O)N2C(=O)[C@@H](NC(=O)Cc3cccs3)[C@H]2SC1 | 0.59 | 7 | 2 | 113.01 | 396.45 | 396.45 |
| -6.8 | colchicine | CCCCCCCCCCCCCCCCOP(=O)([O-])OCC[N+](C)(C)C | 5.68 | 4 | 0 | 58.59 | 407.58 | 407.58 |
| -6.8 | SR-05000001553 | CC(=O)NC[C@H]1CN(c2ccc(N3CCOCC3)c(F)c2)C(=O)O1 | 1.12 | 5 | 1 | 71.11 | 337.35 | 337.35 |
| -6.8 | DENATONIUM BENZOATE | C[N+]12CCC(CC1)C(OC(=O)C(O)(c1ccccc1)c1ccccc1)C2 | 2.7 | 3 | 1 | 46.53 | 352.45 | 352.45 |
| -6.8 | cycloheximide | C[C@@H](O)[C@H]1C(=O)N2C(C(=O)O)=C(S[C@@H]3CN[C@H](C(=O)N(C)C)C3)[C@H](C)[C@H]12 | -0.31 | 6 | 3 | 110.18 | 383.47 | 383.47 |
| -6.8 | arbutin | O=c1n(CCCN2CCN(c3cccc(Cl)c3)CC2)nc2ccccn12 | 2.36 | 6 | 0 | 45.78 | 371.87 | 371.87 |
| -6.8 | N-Acetylthiourea | CCc1cccc2c3c([nH]c12)C(CC)(CC(=O)O)OCC3 | 3.38 | 2 | 2 | 62.32 | 287.36 | 287.36 |
| -6.8 | Helicin | CC(C1=C(CCN(C)C)Cc2ccccc21)c1ccccn1 | 4.15 | 2 | 0 | 16.13 | 292.43 | 292.43 |
| -6.8 | azepan-2-one | O=C(Nc1cccc2c(=O)cc(-c3nnn[nH]3)oc12)c1ccc(OCCCCc2ccccc2)cc1 | 4.63 | 7 | 2 | 123 | 481.51 | 481.51 |
| -6.8 | 2-ETHYLPYRAZINE | CCc1nn(CCCN2CCN(c3cccc(Cl)c3)CC2)c(=O)n1CCOc1ccccc1 | 3.55 | 7 | 0 | 55.53 | 470.02 | 470.02 |
| -6.8 | imidazolidine-2-thione | c1ccc2[nH]c(-c3cscn3)nc2c1 | 2.69 | 3 | 1 | 41.57 | 201.25 | 201.25 |
| -6.7 | Ethylthiourea | CN(C/C=C/c1ccccc1)Cc1cccc2ccccc12 | 4.99 | 1 | 0 | 3.24 | 287.41 | 287.41 |
| -6.7 | methimazole | Cc1cc(=O)n(-c2ccccc2)n1C | 1.48 | 3 | 0 | 26.93 | 188.23 | 188.23 |
| -6.7 | Limonin | CN1C(=O)C(O)N=C(c2ccccc2Cl)c2cc(Cl)ccc21 | 3.13 | 3 | 1 | 52.9 | 335.19 | 335.19 |
| -6.7 | methylthiouracil | CC(=O)CCCCn1c(=O)c2c(ncn2C)n(C)c1=O | 0.19 | 7 | 0 | 78.89 | 278.31 | 278.31 |
| -6.7 | 2-Phenylethyl isothiocyanate | CN(C)CCC=C1c2ccccc2CCc2ccccc21 | 4.17 | 1 | 0 | 3.24 | 277.41 | 277.41 |
| -6.7 | Phenylthiourea | O=C(Nc1ccc(Br)cc1)c1cc(Br)ccc1O | 4.17 | 2 | 2 | 49.33 | 371.03 | 371.03 |
| -6.7 | FSL0315 | O=C(Nc1ccc(Br)cc1)c1cc(Br)cc(Br)c1O | 4.93 | 2 | 2 | 49.33 | 449.92 | 449.92 |
| -6.7 | saccharin | CNS(=O)(=O)Cc1ccc2[nH]cc(CCN(C)C)c2c1 | 1.32 | 3 | 2 | 65.2 | 295.41 | 295.41 |
| -6.7 | Cnicin | CCCNCC(O)COc1ccccc1C(=O)CCc1ccccc1 | 3.24 | 4 | 2 | 58.56 | 341.45 | 341.45 |
| -6.7 | Andrographolide,(S) | C[C@H]1C[C@H]2[C@@H]3CCC4=CC(=O)C=C[C@]4(C)[C@@]3(F)[C@@H](O)C[C@]2(C)[C@@]1(O)C(=O)CO | 1.9 | 5 | 3 | 94.83 | 392.47 | 392.47 |
| -6.7 | Crispolide | CCCCc1oc2ccccc2c1C(=O)c1cc(I)c(OCCN(CC)CC)c(I)c1 | 6.94 | 4 | 0 | 42.68 | 645.32 | 645.32 |
| -6.7 | 6-Methylcoumarin | CCC(=O)N(c1ccccc1)C1(COC)CCN(CCn2nnn(CC)c2=O)CC1 | 1.38 | 8 | 0 | 85.49 | 416.53 | 416.53 |
| -6.7 | GAMMA-TERPINENE | C[C@]12C=CC(=O)C=C1CC[C@@H]1[C@@H]2C(=O)C[C@@]2(C)[C@H]1CC[C@]2(O)C(=O)CO | 1.77 | 5 | 2 | 91.67 | 358.43 | 358.43 |
| -6.7 | ALPHA-TERPINENE | CCN(C)C(=O)Oc1cccc([C@H](C)N(C)C)c1 | 2.76 | 3 | 0 | 32.78 | 250.34 | 250.34 |
| -6.7 | Phenethyl formate | COc1ccc(C(CN(C)C)C2(O)CCCCC2)cc1 | 3.04 | 3 | 1 | 32.7 | 277.41 | 277.41 |
| -6.7 | PIPERONAL | Cc1cn([C@H]2C[C@H](N=[N+]=[N-])[C@@H](CO)O2)c(=O)[nH]c1=O | -0.2 | 6 | 2 | 133.08 | 267.24 | 267.24 |
| -6.7 | METHYL ANTHRANILATE | C[C@@H](c1ncncc1F)[C@](O)(Cn1cncn1)c1ccc(F)cc1F | 2.18 | 6 | 1 | 76.72 | 349.32 | 349.32 |
| -6.7 | 2-METHOXYBENZALDEHYDE | CN1CCCC(n2nc(Cc3ccc(Cl)cc3)c3ccccc3c2=O)CC1 | 4.3 | 4 | 0 | 38.13 | 381.91 | 381.91 |
| -6.7 | PIPERONYL ACETATE | O=C(N[C@H](CO)[C@H](O)c1ccc([N+](=O)[O-])cc1)C(Cl)Cl | 0.91 | 5 | 3 | 112.7 | 323.13 | 323.13 |
| -6.7 | Terpinyl formate | CCN(CC)CCNC(=O)c1ccc(N)cc1 | 1.34 | 3 | 2 | 58.36 | 235.33 | 235.33 |
| -6.7 | 2-(Isopropyl)-5-methylcyclohexyl 2-methylbutyrate | CNCC[C@@H](Oc1ccccc1C)c1ccccc1 | 3.72 | 2 | 1 | 21.26 | 255.36 | 255.36 |
| -6.7 | 3-Phenylpropyl acetate | CCCC(=O)Nc1ccc(OCC(O)CNC(C)C)c(C(C)=O)c1 | 2.37 | 5 | 3 | 87.66 | 336.43 | 336.43 |
| -6.7 | 4-Methoxybenzaldehyde | CC(CN1c2ccccc2Sc2ccccc21)N(C)C | 4.24 | 3 | 0 | 6.48 | 284.43 | 284.43 |
| -6.7 | alpha-Terpinyl anthranilate | C[C@]12C=CC(=O)C=C1CC[C@@H]1[C@@H]2[C@@H](O)C[C@@]2(C)[C@H]1CC[C@]2(O)C(=O)CO | 1.56 | 5 | 3 | 94.83 | 360.45 | 360.45 |
| -6.7 | Terpinyl propionate | CC(CN(C)C)CN1c2ccccc2CCc2ccccc21 | 4.12 | 2 | 0 | 6.48 | 294.44 | 294.44 |
| -6.7 | Alpha-terpinyl isovalerate | CC(C)NCC(O)COc1ccc(COCCOC(C)C)cc1 | 2.37 | 5 | 2 | 59.95 | 325.45 | 325.45 |
| -6.7 | 3,7-Dimethyl-2,6-octadien-1-ol | Cc1nnc2n1-c1ccc(Cl)cc1C(c1ccccc1Cl)=NC2 | 4.23 | 4 | 0 | 43.07 | 343.22 | 343.22 |
| -6.7 | genistein | Nc1cc(Cl)c(N=C2NCCN2)c(Cl)c1 | 1.76 | 2 | 3 | 62.44 | 245.11 | 245.11 |
| -6.7 | daidzein | CN1CCN(C(c2ccccc2)c2ccccc2)CC1 | 3.02 | 2 | 0 | 6.48 | 266.39 | 266.39 |
| -6.7 | Prunetin | OC(CNCC(O)C1CCc2cc(F)ccc2O1)C1CCc2cc(F)ccc2O1 | 2.36 | 5 | 3 | 70.95 | 405.44 | 405.44 |
| -6.7 | 1-TETRALONE | C[C@H]1C[C@@H]2[C@H]([C@@H](O)C[C@@]3(C)[C@H]2CC[C@]3(O)C(=O)CO)[C@@]2(C)C=CC(=O)C=C12 | 1.8 | 5 | 3 | 94.83 | 374.48 | 374.48 |
| -6.7 | berberine | CCC(=O)C(CC(C)N(C)C)(c1ccccc1)c1ccccc1 | 4.29 | 2 | 0 | 20.31 | 309.45 | 309.45 |
| -6.7 | Tangeretin | O=C(NCC1CCCCN1)c1cc(OCC(F)(F)F)ccc1OCC(F)(F)F | 3.44 | 4 | 2 | 59.59 | 414.35 | 414.35 |
| -6.7 | Cianidanol | Clc1cccc(Cl)c1NC1=NCCN1 | 2.36 | 3 | 2 | 36.42 | 230.1 | 230.1 |
| -6.7 | Sinensetin | CN1CCN2c3ncccc3Cc3ccccc3C2C1 | 2.48 | 3 | 0 | 19.37 | 265.36 | 265.36 |
| -6.7 | Nobiletin | Cc1ncc2n1-c1ccc(Cl)cc1C(c1ccccc1F)=NC2 | 4.32 | 3 | 0 | 30.18 | 325.77 | 325.77 |
| -6.7 | 3-(4-hydroxyphenyl)-7-{[(3R,4S,5S,6R)-3,4,5-trihydroxy-6-(hydroxymethyl)oxan-2-yl]oxy}-4H-chromen-4-one | CCN1CCC[C@H]1CNC(=O)c1c(OC)ccc(Br)c1OC | 2.68 | 4 | 1 | 50.8 | 371.28 | 371.28 |
| -6.7 | COUMESTROL | COc1ccc2c3c1O[C@H]1C(=O)CC[C@@]4(O)[C@@H](C2)N(C)CC[C@]314 | 1.05 | 5 | 1 | 59 | 315.37 | 315.37 |
| -6.7 | TRANS-2-(4-CHLOROPHENYL)VINYLBORONICACID | C[C@]12CC[C@@H]3c4ccc(O)cc4CC[C@H]3[C@@H]1CC[C@@H]2O | 3.61 | 2 | 2 | 40.46 | 272.39 | 272.39 |
| -6.7 | (E)-4-Isopropylbenzaldehyde oxime | CN(C)CCOC(c1ccccc1)c1ccccc1 | 3.35 | 2 | 0 | 12.47 | 255.36 | 255.36 |
| -6.7 | 6-nitro-2-(propan-2-yl)-2,3-dihydro-1??,2-benzothiazole-1,1,3-trione | CCC(=O)N(c1ccccc1)C1(COC)CCN(CCc2cccs2)CC1 | 4.21 | 4 | 0 | 32.78 | 386.56 | 386.56 |
| -6.7 | 2-butyl-6-nitro-2,3-dihydro-1??,2-benzothiazole-1,1,3-trione | COc1ccc2c3c1O[C@H]1C[C@@H](O)C=C[C@@]31CCN(C)C2 | 1.85 | 4 | 1 | 41.93 | 287.36 | 287.36 |
| -6.7 | 6-nitro-2-pentyl-2,3-dihydro-1??,2-benzothiazole-1,1,3-trione | NC12CC3CC(CC(C3)C1)C2 | 1.91 | 1 | 1 | 26.02 | 151.25 | 151.25 |
| -6.7 | 6-nitro-2-[(trichloromethyl)sulfanyl]-2,3-dihydro-1??,2-benzothiazole-1,1,3-trione | Cc1nnc2n1-c1ccc(Cl)cc1C(c1ccccc1)=NC2 | 3.58 | 4 | 0 | 43.07 | 308.77 | 308.77 |
| -6.7 | 2-[2-(diethylamino)ethyl]-1,1-dioxo-1,2-benzothiazol-3-one | CNCCCC12CCC(c3ccccc31)c1ccccc12 | 4.21 | 1 | 1 | 12.03 | 277.41 | 277.41 |
| -6.7 | 2-[3-(diethylamino)propyl]-1,1-dioxo-1,2-benzothiazol-3-one | O=C(O)P(=O)(O)O | -0.16 | 2 | 3 | 94.83 | 126 | 126 |
| -6.7 | 6-nitro-2-(prop-2-en-1-yl)-2,3-dihydro-1??,2-benzothiazole-1,1,3-trione | CC(=O)OCC[N+](C)(C)C | 0.26 | 2 | 0 | 26.3 | 146.21 | 146.21 |
| -6.7 | MLS000673467 | Cc1ncc([N+](=O)[O-])n1CCO | 0.09 | 5 | 1 | 81.19 | 171.16 | 171.16 |
| -6.7 | 3-(propan-2-yloxy)-1,2-benzothiazole 1,1-dioxide | CNCCCC1c2ccccc2C=Cc2ccccc21 | 4.3 | 1 | 1 | 12.03 | 263.38 | 263.38 |
| -6.7 | sulfamerazine | CN(C)CCC=C1c2ccccc2C=Cc2ccccc21 | 4.55 | 1 | 0 | 3.24 | 275.4 | 275.4 |
| -6.7 | sulfameter | S=P(N1CC1)(N1CC1)N1CC1 | 0.16 | 1 | 0 | 9.03 | 189.22 | 189.22 |
| -6.7 | sulfamethoxypyridazine | CC(C)OC(=O)C(C)(C)Oc1ccc(C(=O)c2ccc(Cl)cc2)cc1 | 4.68 | 4 | 0 | 52.6 | 360.84 | 360.84 |
| -6.7 | suxibuzone | C#CCN(C)Cc1ccccc1 | 1.75 | 1 | 0 | 3.24 | 159.23 | 159.23 |
| -6.7 | TALBUTAL | N=C(N)c1ccc(OCCCCCCOc2ccc(C(=N)N)cc2)cc1 | 3.27 | 4 | 4 | 118.2 | 354.45 | 354.45 |
| -6.7 | (5r,6s)-5-ethenyl-4a-hydroxy-6-[(2s,3r,4s,5s,6r)-3,4,5-trihydroxy-6-(hydroxymethyl)oxan-2-yl]oxy-3,4,5,6-tetrahydropyrano[5,4-c]pyran-1-one | NCCCC(O)(P(=O)([O-])O)P(=O)(O)O.[Na+] | -1.27 | 4 | 6 | 161.31 | 249.1 | 271.08 |
| -6.7 | Tetraethylammonium hydroxide | C[C@H](N)[C@H](O)c1ccc(O)c(O)c1 | 0.48 | 4 | 4 | 86.71 | 183.21 | 183.21 |
| -6.7 | Theophylline sodium acetate | CNC[C@H](O)c1ccc(O)c(O)c1 | 0.35 | 4 | 4 | 72.72 | 183.21 | 183.21 |
| -6.7 | Thiosemicarbazone | N[C@@H](C(=O)N[C@@H]1C(=O)N2C(C(=O)O)=C(Cl)CS[C@H]12)c1ccccc1 | 0.62 | 5 | 3 | 112.73 | 367.81 | 367.81 |
| -6.7 | THIAMINE HYDROCHLORIDE | CCOC(=O)c1cncn1[C@H](C)c1ccccc1 | 2.67 | 4 | 0 | 44.12 | 244.29 | 244.29 |
| -6.7 | tiaramide hydrochloride | CCN(CC)Cc1cc(Nc2ccnc3cc(Cl)ccc23)ccc1O | 5.18 | 4 | 2 | 48.39 | 355.87 | 355.87 |
| -6.7 | Tridihexethyl | CCN(CC)C(=O)N1CCN(C)CC1 | 0.7 | 2 | 0 | 26.79 | 199.3 | 199.3 |
| -6.7 | trapidil | COC(=O)Nc1nc2cc(C(=O)c3ccccc3)ccc2[nH]1 | 2.97 | 4 | 2 | 84.08 | 295.3 | 295.3 |
| -6.7 | TRIMETHAPHAN | O=C(O)c1ccccc1Nc1cccc(C(F)(F)F)c1 | 4.15 | 2 | 2 | 49.33 | 281.23 | 281.23 |
| -6.7 | trimethoprim | O=C(O)Cc1ccccc1Nc1c(Cl)cccc1Cl | 4.36 | 2 | 2 | 49.33 | 296.15 | 296.15 |
| -6.7 | carnosol | Cc1cccc(Nc2ccccc2C(=O)O)c1C | 3.75 | 2 | 2 | 49.33 | 241.29 | 241.29 |
| -6.7 | cycrimine | CCC(=O)N(c1ccccc1)C1CCN(CCc2ccccc2)CC1.O=C(O)CC(O)(CC(=O)O)C(=O)O | 4.14 | 2 | 0 | 23.55 | 336.48 | 528.6 |
| -6.7 | Metalkonium chloride | CN1CCC2=C(C1)C(c1ccccc1)c1ccccc12 | 3.92 | 1 | 0 | 3.24 | 261.37 | 261.37 |
| -6.7 | Ethylbenzhydramine hydrochloride | OC[C@@H](O)[C@@H](O)[C@H](O)[C@H](O)CO | -3.59 | 6 | 6 | 121.38 | 182.17 | 182.17 |
| -6.7 | Butallylonal | CC[C@H](C)C(=O)O[C@H]1C[C@H](O)C=C2C=C[C@H](C)[C@H](CC[C@@H](O)C[C@@H](O)CC(=O)[O-])[C@H]21.[Na+] | 2.44 | 6 | 4 | 124.29 | 424.53 | 446.52 |
| -6.7 | CHLORBETAMIDE | CC(Cc1ccccc1)NCCC(c1ccccc1)c1ccccc1 | 5.43 | 1 | 1 | 12.03 | 329.49 | 329.49 |
| -6.7 | Camphotamide | COc1c2ccoc2cc2oc(=O)ccc12 | 2.55 | 4 | 0 | 52.58 | 216.19 | 216.19 |
| -6.7 | Chloralodol | C#C[C@]1(O)CC[C@H]2[C@@H]3CCc4cc(O)ccc4[C@H]3CC[C@@]21C | 3.61 | 2 | 2 | 40.46 | 296.41 | 296.41 |
| -6.7 | 5-(2-bromoprop-2-enyl)-5-butan-2-yl-2-hydroxy-1H-pyrimidine-4,6-dione | Nc1ccncc1 | 0.66 | 2 | 1 | 38.91 | 94.12 | 94.12 |
| -6.7 | Cyclopentobarbital | OCC(O)CO | -1.67 | 3 | 3 | 60.69 | 92.09 | 92.09 |
| -6.7 | 5-ethyl-5-hexyl-2-hydroxy-1H-pyrimidine-4,6-dione | CN1C(=O)OC(C)(C)C1=O | 0.37 | 3 | 0 | 46.61 | 143.14 | 143.14 |
| -6.7 | Hexetone | CCC1(C)CC(=O)NC1=O | 0.45 | 2 | 1 | 46.17 | 141.17 | 141.17 |
| -6.7 | Narcobarbital | CN1C(=O)CC(C)(c2ccccc2)C1=O | 1.33 | 2 | 0 | 37.38 | 203.24 | 203.24 |
| -6.7 | p-Lactophenetide | CCCCNc1ccc(C(=O)OCCN(C)C)cc1 | 2.62 | 4 | 1 | 41.57 | 264.37 | 264.37 |
| -6.7 | Pentamethonium bromide | CNCCc1ccccn1 | 0.84 | 2 | 1 | 24.92 | 136.2 | 136.2 |
| -6.7 | 5-(2-methylbutan-2-yl)-5-(prop-2-en-1-yl)-1,3-diazinane-2,4,6-trione | Nc1ccc(S(=O)(=O)Nc2ccccn2)cc1 | 1.46 | 4 | 2 | 85.08 | 249.3 | 249.3 |
| -6.7 | Phenallymal | NCC(CC(=O)O)c1ccc(Cl)cc1 | 1.86 | 2 | 2 | 63.32 | 213.66 | 213.66 |
| -6.7 | Phenylmethylbarbituric acid | Oc1cccc(O)c1 | 1.1 | 2 | 2 | 40.46 | 110.11 | 110.11 |
| -6.7 | barbital | CCN1CCN(C(=O)N[C@@H](C(=O)N[C@@H]2C(=O)N3[C@@H]2SC(C)(C)[C@@H]3C(=O)O)c2ccccc2)C(=O)C1=O | -0.24 | 7 | 3 | 156.43 | 517.56 | 517.56 |
| -6.7 | Acranil | C[N+](C)(C)CCOC(=O)CCC(=O)OCC[N+](C)(C)C | 0.27 | 4 | 0 | 52.6 | 290.4 | 290.4 |
| -6.7 | DTXSID1023738 | Nc1ccc(O)c(C(=O)O)c1 | 0.67 | 3 | 3 | 83.55 | 153.14 | 153.14 |
| -6.7 | Barbital sodium | CC1=C(/C=C/C(C)=C\C=C\C(C)=C\C(=O)O)C(C)(C)CCC1 | 5.6 | 1 | 1 | 37.3 | 300.44 | 300.44 |
| -6.7 | BENZILIC ACID | CCCCCCCCCCCCCCCCO | 5.46 | 1 | 1 | 20.23 | 242.45 | 242.45 |
| -6.7 | Bretylium | C[C@H](Cc1cc2c(c(C(N)=O)c1)N(CCCO)CC2)NCCOc1ccccc1OCC(F)(F)F | 3.07 | 6 | 3 | 97.05 | 495.54 | 495.54 |
| -6.7 | 5-butan-2-yl-5-ethyl-2-hydroxy-1H-pyrimidine-4,6-dione | C[N+](C)(C)CCO.[Cl-] |  |  |  |  | 139.63 | 139.63 |
| -6.7 | butalbital | COc1cc2nc(N3CCN(C(=O)C4COc5ccccc5O4)CC3)nc(N)c2cc1OC | 1.72 | 9 | 1 | 112.27 | 451.48 | 451.48 |
| -6.7 | Butethal | O=C(NC1CCN(CCc2c[nH]c3ccccc23)CC1)c1ccccc1 | 3.6 | 2 | 2 | 48.13 | 347.46 | 347.46 |
| -6.7 | 4-Hydroxybenzenesulfonic acid | COc1cc2/c(=N/c3ccc(Br)cc3F)nc[nH]c2cc1OCC1CCN(C)CC1 | 4.43 | 5 | 1 | 62.74 | 475.36 | 475.36 |
| -6.7 | Carbimazole | Cc1cc(-c2ccccc2)nnc1NCCN1CCOCC1 | 2.2 | 5 | 1 | 50.28 | 298.39 | 298.39 |
| -6.7 | 5,2'-Dihydroxyflavone | CC1(C)CCC(C)(C)c2cc(NC(=O)c3ccc(C(=O)O)cc3)ccc21 | 4.99 | 2 | 2 | 66.4 | 351.45 | 351.45 |
| -6.7 | 4',5-Dihydroxyflavone | O=C1Cc2cc(CCN3CCN(c4nsc5ccccc45)CC3)c(Cl)cc2N1 | 3.81 | 5 | 1 | 48.47 | 412.95 | 412.95 |
| -6.7 | 7,4'-Dihydroxyflavone | COc1cc2nc(N(C)CCCNC(=O)C3CCCO3)nc(N)c2cc1OC | 1.35 | 8 | 2 | 111.83 | 389.46 | 389.46 |
| -6.7 | 6,4'-Dihydroxyflavone | CC(C)(C)NC(=O)[C@H]1CC[C@H]2[C@@H]3CC[C@H]4NC(=O)C=C[C@]4(C)[C@H]3CC[C@]12C | 3.81 | 2 | 2 | 58.2 | 372.55 | 372.55 |
| -6.7 | 5,7-Dimethoxyflavone | O=C1c2ccccc2C(=O)C1c1ccccc1 | 2.85 | 2 | 0 | 34.14 | 222.24 | 222.24 |
| -6.7 | 6,7-dimethoxyflavone | COc1ccc(C2C(=O)c3ccccc3C2=O)cc1 | 2.86 | 3 | 0 | 43.37 | 252.27 | 252.27 |
| -6.7 | FLAVONE | O=C1c2ccccc2C(=O)C1c1ccc(F)cc1 | 2.99 | 2 | 0 | 34.14 | 240.23 | 240.23 |
| -6.7 | 5-Hydroxyflavone | C=C1[C@H](CO)[C@@H](O)C[C@@H]1n1cnc2c(=O)[nH]c(N)nc21 | -0.83 | 7 | 4 | 130.05 | 277.28 | 277.28 |
| -6.7 | 4'-Hydroxyflavone | Nc1ccn([C@@H]2CS[C@H](CO)O2)c(=O)n1 | -0.59 | 7 | 2 | 90.37 | 229.26 | 229.26 |
| -6.7 | 4'-HYDROXY-6-METHOXYFLAVONE | CC(C)(C)NCC(O)c1ccc(O)c(CO)c1 | 1.31 | 4 | 4 | 72.72 | 239.31 | 239.31 |
| -6.7 | Isopratol | Cc1cc2c(s1)Nc1ccccc1N=C2N1CCN(C)CC1 | 3.44 | 5 | 1 | 30.87 | 312.44 | 312.44 |
| -6.7 | 6-Methoxyluteolin | OCCOCCN1CCN(C2=Nc3ccccc3Sc3ccccc32)CC1 | 2.86 | 6 | 1 | 48.3 | 383.52 | 383.52 |
| -6.7 | luteolin | CN(C)CCOC1=Cc2ccccc2Sc2ccc(Cl)cc21 | 4.88 | 3 | 0 | 12.47 | 331.87 | 331.87 |
| -6.7 | Tricetin | CN1CCN=C(c2ccccc2)c2cc(Cl)ccc21 | 3.63 | 2 | 0 | 15.6 | 270.76 | 270.76 |
| -6.7 | Scutellarein | CC(=O)O[C@]1(C(C)=O)CC[C@H]2[C@@H]3C[C@H](C)C4=CC(=O)CC[C@]4(C)[C@H]3CC[C@@]21C | 4.66 | 4 | 0 | 60.44 | 386.53 | 386.53 |
| -6.7 | 5,7,2'-Trihydroxyflavone | O=C(CCCN1CCC(O)(c2ccc(Br)cc2)CC1)c1ccc(F)cc1 | 4.53 | 3 | 1 | 40.54 | 420.32 | 420.32 |
| -6.7 | 7,3',4'-Trihydroxyflavone | C/C(=C\C(=O)OCCCCCCCCC(=O)O)C[C@@H]1OC[C@H](C[C@@H]2O[C@H]2[C@@H](C)[C@H](C)O)[C@@H](O)[C@H]1O | 2.59 | 8 | 4 | 146.05 | 500.63 | 500.63 |
| -6.7 | 4',5,7-Trimethoxyflavone | OCc1ccccc1 | 1.18 | 1 | 1 | 20.23 | 108.14 | 108.14 |
| -6.7 | Datiscetin | CCN(CC)C(=O)Nc1ccc(OCC(O)CNC(C)(C)C)c(C(C)=O)c1 | 2.89 | 5 | 3 | 90.9 | 379.5 | 379.5 |
| -6.6 | Fisetin | CC(C)(C)NCC(O)COc1cccc2c1SCCC2 | 2.85 | 4 | 2 | 41.49 | 295.45 | 295.45 |
| -6.6 | Herbacetin | COc1ccccc1OCCNCC(O)COc1cccc2[nH]c3ccccc3c12 | 3.74 | 5 | 3 | 75.74 | 406.48 | 406.48 |
| -6.6 | morin | OCCN1CCN(CCCN2c3ccccc3Sc3ccc(C(F)(F)F)cc32)CC1 | 4.31 | 5 | 1 | 29.95 | 437.53 | 437.53 |
| -6.6 | 3,7,4'-Trihydroxyflavone | Nc1nc2[nH]cnc2c(=S)[nH]1 | 0.6 | 4 | 3 | 83.38 | 167.2 | 167.2 |
| -6.6 | 3,6,3',4'-TETRAHYDROXYFLAVONE | C[C@H](CCc1ccccc1)NC[C@H](O)c1ccc(O)c(C(N)=O)c1 | 2.14 | 4 | 4 | 95.58 | 328.41 | 328.41 |
| -6.6 | hesperetin | CN1CCN(CCCN2c3ccccc3Sc3ccc(Cl)cc32)CC1 | 4.58 | 4 | 0 | 9.72 | 373.95 | 373.95 |
| -6.6 | Homoeriodictyol | Cc1cccc(C)c1OCC(=O)N[C@@H](Cc1ccccc1)[C@@H](O)C[C@H](Cc1ccccc1)NC(=O)[C@H](C(C)C)N1CCCNC1=O | 4.33 | 5 | 4 | 120 | 628.81 | 628.81 |
| -6.6 | 2',3,4,4'-tetrahydroxy Chalcone | O=[N+]([O-])OCC(CO[N+](=O)[O-])O[N+](=O)[O-] | -1.02 | 9 | 0 | 157.11 | 227.08 | 227.08 |
| -6.6 | Cinnamoylbenzene | CCn1cc(C(=O)O)c(=O)c2cnc(N3CCNCC3)nc21 | -0.08 | 7 | 2 | 100.35 | 303.32 | 303.32 |
| -6.6 | silibinin | CC(=O)NO | -0.49 | 2 | 2 | 49.33 | 75.07 | 75.07 |
| -6.6 | (3R)-2-(3,4-dihydroxyphenyl)-3,5,7-trihydroxy-2,3-dihydro-4H-chromen-4-one | O=C(NCCO[N+](=O)[O-])c1cccnc1 | 0.02 | 5 | 1 | 94.36 | 211.18 | 211.18 |
| -6.6 | MLS002693861 | CCN(CCCCOC(=O)c1ccc(OC)c(OC)c1)C(C)Cc1ccc(OC)cc1 | 4.6 | 6 | 0 | 57.23 | 429.56 | 429.56 |
| -6.6 | CRBYNQCDRNZCNX-UHFFFAOYSA-N | CCCC(C)C1(CC)C(=O)[N-]C(=S)NC1=O.[Na+] | 1.35 | 3 | 2 | 58.2 | 242.34 | 264.33 |
| -6.6 | Spectrum_000721 | Cc1ccc(C(C)C)c(O)c1 | 2.82 | 1 | 1 | 20.23 | 150.22 | 150.22 |
| -6.6 | GU17;ISL;Isoliquiritigen | C=CCC1(C(C)C#CCC)C(=O)N=C([O-])N(C)C1=O.[Na+] | 1.31 | 3 | 1 | 66.48 | 262.31 | 284.29 |
| -6.6 | 2',4',2-Trihydroxychalcone | Nc1nnc(-c2cccc(Cl)c2Cl)c(N)n1 | 2.01 | 5 | 2 | 90.71 | 256.1 | 256.1 |
| -6.6 | 2-Propen-1-one, 1-(2,5-dihydroxyphenyl)-3-(4-hydroxyphenyl)- | Fc1ccc(C(c2ccc(F)cc2)N2CCN(C/C=C/c3ccccc3)CC2)cc1 | 5.39 | 2 | 0 | 6.48 | 404.5 | 404.5 |
| -6.6 | Cyanidin | CNC1(c2ccccc2Cl)CCCCC1=O | 2.9 | 2 | 1 | 29.1 | 237.73 | 237.73 |
| -6.6 | phloretin | Nc1nc2ccc(OC(F)(F)F)cc2s1 | 2.78 | 4 | 1 | 48.14 | 234.2 | 234.2 |
| -6.6 | biochanin A | CCCc1c2oc(C(=O)O)cc(=O)c2cc2c(=O)cc(C(=O)O)n(CC)c12 | 2.48 | 6 | 2 | 126.81 | 371.35 | 371.35 |
| -6.6 | 4',7-Dimethoxyisoflavone | C[n+]1ccccc1/C=N/O.[Cl-] | 0.32 | 2 | 1 | 36.47 | 137.16 | 172.62 |
| -6.6 | Glycitein | C[C@]12CC[C@@H]3c4ccc(OC(=O)c5ccccc5)cc4CC[C@H]3[C@@H]1CC[C@@H]2O | 5.12 | 3 | 1 | 46.53 | 376.5 | 376.5 |
| -6.6 | 7-Hydroxyisoflavone | NS(=O)(=O)Cc1noc2ccccc12 | 0.62 | 4 | 1 | 86.19 | 212.23 | 212.23 |
| -6.6 | Isoflavone | Nc1ncnc2c1ncn2[C@@H]1O[C@H](COP(=O)(O)O)[C@@H](O)[C@H]1O | -1.86 | 10 | 5 | 186.07 | 347.22 | 347.22 |
| -6.6 | Glycitin | CC(COc1ccccc1)N(CCCl)Cc1ccccc1 | 4.19 | 2 | 0 | 12.47 | 303.83 | 303.83 |
| -6.6 | 17817-31-1 | O=C([O-])P(=O)([O-])[O-].[Na+].[Na+].[Na+] | -0.16 | 2 | 3 | 94.83 | 126 | 191.95 |
| -6.6 | 3',4',7-Trihydroxyisoflavone | O=C1CN2Cc3c(ccc(Cl)c3Cl)N=C2N1 | 1.93 | 3 | 1 | 44.7 | 256.09 | 256.09 |
| -6.6 | 75187-63-2 | c1ccc2c(CC3=NCCN3)cccc2c1 | 2.38 | 2 | 1 | 24.39 | 210.28 | 210.28 |
| -6.6 | 1,3-Benzenediol, 5-[(1Z)-2-(4-hydroxyphenyl)ethenyl]- | Cc1cc(C(C)(C)C)c(O)c(C)c1CC1=NCCN1 | 2.85 | 3 | 2 | 44.62 | 260.38 | 260.38 |
| -6.6 | 6,3',4'-Trihydroxyaurone | N=C(N)NCCN1CCCCCCC1 | 0.74 | 2 | 3 | 65.14 | 198.31 | 198.31 |
| -6.6 | XANTHONE | CC1(C)C[C@@H]1C(=O)N/C(=C\CCCCSC[C@H](N)C(=O)O)C(=O)O | 1.43 | 5 | 4 | 129.72 | 358.46 | 358.46 |
| -6.6 | 4-Methoxyphenol | COC(=O)CCc1ccc(OCC(O)CNC(C)C)cc1 | 1.53 | 5 | 2 | 67.79 | 295.38 | 295.38 |
| -6.6 | 1-NAPHTHOIC ACID | c1ccc(CC2=NCCN2)cc1 | 1.23 | 2 | 1 | 24.39 | 160.22 | 160.22 |
| -6.6 | 5811-87-0 | N[C@@H]1CONC1=O | -1.62 | 3 | 2 | 64.35 | 102.09 | 102.09 |
| -6.6 | PIPERONYLIC ACID | C[C@@H](O)[C@H]1C(=O)N2C(C(=O)O)=C(SCCNC=N)C[C@H]12 | -0.18 | 5 | 4 | 113.72 | 299.35 | 299.35 |
| -6.6 | picrotin | COC(=O)[C@H]1[C@H]2C[C@@H]3c4[nH]c5cc(OC)ccc5c4CCN3C[C@H]2C[C@@H](OC(=O)c2cc(OC)c(OC)c(OC)c2)[C@@H]1OC | 4.17 | 10 | 1 | 117.78 | 608.69 | 608.69 |
| -6.6 | formononetin | NCC(=O)O | -0.97 | 2 | 2 | 63.32 | 75.07 | 75.07 |
| -6.6 | Pemirolast | CC(C)NCC(O)c1cc(O)cc(O)c1 | 1.13 | 4 | 4 | 72.72 | 211.26 | 211.26 |
| -6.6 | mefenamic acid | O=C(O)[C@H]1/C(=C/CO)O[C@@H]2CC(=O)N21 | -1.1 | 4 | 2 | 87.07 | 199.16 | 199.16 |
| -6.6 | Salsalate | Cc1cccc([C@H](C)c2c[nH]cn2)c1C | 3.18 | 1 | 1 | 28.68 | 200.28 | 200.28 |
| -6.6 | sodium [3-[[2-(carboxylatomethoxy)benzoyl]amino]-2-methoxy-propyl]mercury | CN1CC(=O)N2[C@H](c3ccc4c(c3)OCO4)c3[nH]c4ccccc4c3C[C@@H]2C1=O | 2.21 | 4 | 1 | 74.87 | 389.41 | 389.41 |
| -6.6 | methadone | Oc1ccc2c(c1)[C@@]13CCCC[C@@]1(O)[C@@H](C2)N(CC1CCC1)CC3 | 3.37 | 3 | 2 | 43.7 | 327.47 | 327.47 |
| -6.6 | [1-(2-methoxyphenyl)propyl](methyl)amine | O=C(O)CN(CCN(CC(=O)O)CC(=O)O)CCN(CC(=O)O)CC(=O)O | -2.68 | 8 | 5 | 196.22 | 393.35 | 393.35 |
| -6.6 | 5,5'-Methylenedisalicylic acid | OC1(c2ccc(Cl)cc2)c2ccccc2C2=NCCN21 | 2.61 | 3 | 1 | 35.83 | 284.75 | 284.75 |
| -6.6 | methyprylon | Oc1c(Cl)cc(Cl)cc1Sc1cc(Cl)cc(Cl)c1O | 5.86 | 3 | 2 | 40.46 | 356.06 | 356.06 |
| -6.6 | Anethole trithione | CCCCNC(=O)NS(=O)(=O)c1ccc(C)cc1 | 1.78 | 3 | 2 | 75.27 | 270.35 | 270.35 |
| -6.6 | Nikethamide | CC(C)[C@H]1CC[C@H](C(=O)N[C@H](Cc2ccccc2)C(=O)O)CC1 | 3.26 | 2 | 2 | 66.4 | 317.43 | 317.43 |
| -6.6 | Nialamide | O=C(O)CCCCC1CCSS1 | 2.79 | 3 | 1 | 37.3 | 206.33 | 206.33 |
| -6.6 | neostigmine bromide | CC/C(=C(\c1ccccc1)c1ccc(OCCN(C)C)cc1)c1ccccc1.O=C(O)CC(O)(CC(=O)O)C(=O)O | 6 | 2 | 0 | 12.47 | 371.52 | 563.65 |
| -6.6 | 59-87-0 | CC(C)(O)c1ccccc1CC[C@@H](SCC1(CC(=O)O)CC1)c1cccc(/C=C/c2ccc3ccc(Cl)cc3n2)c1 | 8.95 | 4 | 2 | 70.42 | 586.2 | 586.2 |
| -6.6 | antazoline | O=c1[nH]c(=O)n([C@H]2C[C@H](O)[C@@H](CO)O2)cc1I | -1.22 | 6 | 3 | 104.55 | 354.1 | 354.1 |
| -6.6 | antipyrine | CO[C@@H]1[C@@H](OC(N)=O)[C@@H](O)[C@H](Oc2ccc3c(O)c(NC(=O)c4ccc(O)c(CC=C(C)C)c4)c(=O)oc3c2C)OC1(C)C | 3.63 | 11 | 5 | 200.01 | 612.63 | 612.63 |
| -6.6 | pentamidine | Nc1ccn(C[C@@H](CO)OCP(=O)(O)O)c(=O)n1 | -1.66 | 7 | 4 | 147.9 | 279.19 | 279.19 |
| -6.6 | 5-ethyl-2-hydroxy-5-pentan-2-yl-1H-pyrimidine-4,6-dione | N=C(NCCCCCCNC(=N)NC(=N)Nc1ccc(Cl)cc1)NC(=N)Nc1ccc(Cl)cc1 | 4.18 | 4 | 10 | 167.58 | 505.46 | 505.46 |
